# Comprehensive Human Transcription Factor Binding Site Map for Combinatory Binding Motifs Discovery

**DOI:** 10.1371/journal.pone.0049086

**Published:** 2012-11-28

**Authors:** Arnoldo J. Müller-Molina, Hans R. Schöler, Marcos J. Araúzo-Bravo

**Affiliations:** 1 Computational Biology and Bioinformatics Group, Max Planck Institute for Molecular Biomedicine, Münster, Germany; 2 Department of Cell and Developmental Biology, Max Planck Institute for Molecular Biomedicine, Münster, Germany; 3 Medical Faculty, University of Münster, Münster, Germany; University of Toronto, Canada

## Abstract

To know the map between transcription factors (TFs) and their binding sites is essential to reverse engineer the regulation process. Only about 10%–20% of the transcription factor binding motifs (TFBMs) have been reported. This lack of data hinders understanding gene regulation. To address this drawback, we propose a computational method that exploits never used TF properties to discover the missing TFBMs and their sites in all human gene promoters. The method starts by predicting a dictionary of regulatory “DNA words.” From this dictionary, it distills 4098 novel predictions. To disclose the crosstalk between motifs, an additional algorithm extracts TF combinatorial binding patterns creating a collection of TF regulatory syntactic rules. Using these rules, we narrowed down a list of 504 novel motifs that appear frequently in syntax patterns. We tested the predictions against 509 known motifs confirming that our system can reliably predict *ab initio* motifs with an accuracy of 81%—far higher than previous approaches. We found that on average, 90% of the discovered combinatorial binding patterns target at least 10 genes, suggesting that to control in an independent manner smaller gene sets, supplementary regulatory mechanisms are required. Additionally, we discovered that the new TFBMs and their combinatorial patterns convey biological meaning, targeting TFs and genes related to developmental functions. Thus, among all the possible available targets in the genome, the TFs tend to regulate other TFs and genes involved in developmental functions. We provide a comprehensive resource for regulation analysis that includes a dictionary of “DNA words,” newly predicted motifs and their corresponding combinatorial patterns. Combinatorial patterns are a useful filter to discover TFBMs that play a major role in orchestrating other factors and thus, are likely to lock/unlock cellular functional clusters.

## Introduction

Gene expression is regulated by the attachment of transcription factors (TFs) onto DNA binding sites located in promoter or enhancer gene regions. Each TF has a propensity to bind to a specific set of binding sites. This set can be represented by a binding motif [1]. Currently, only 10%–20% of the total human TF binding motifs (TFBMs) have been identified [2], [3]. The most widely used databases of experimentally validated TFBMs are Jaspar [4] and Transfac [5]. Considering only globally traceable TFBSs (those for which the collection of all the *loci* targets is documented in the database across the whole genome), 228 and 281 TFBMs exist in Jaspar and Transfac databases, respectively. It is estimated that the total number of human TFs ranges between 1400 [2] and 2600 [3], hence 80% to 90% of the TFBMs are unknown. Furthermore, there are TFs that use more than a TFBM, originating the so called secondary motifs [6]. Such motifs add major variation and complexity to the TFBM repertoire implying a much higher number of unknown motifs. Thus, to gain a comprehensive understanding of the gene regulation process, it is necessary to discover the unknown TFBMs set. Once this set of TFBMs is predicted, the crosstalk with their corresponding TFBSs can be analyzed in a more comprehensive way.

The average length of the known TFBMs is 11.53 base pairs (see section S1.1 in Supporting Information S1). This is shorter than the required length to achieve enough binding specificity (30 information bits as shown by Wunderlich *et al.* [7]). The TFBMs of eukaryotes are shorter than those of prokaryotes [7], and therefore their binding specificity is lower. To compensate for this effect, gene expression in eukaryotes is regulated by the orchestration of multiple TFs [8]. By clustering several short motifs together, the effective spanned length of the combined pattern achieves a greater level of specificity. Given all TFBMs and their corresponding binding sites, it is worthy to extract common syntactic structures that arise in the promoter binding topologies since such structures can constitute regulatory rules that provide additional insights in the transcriptional regulation process. Such rules can also be used to select TFBMs that frequently appear with other motifs and therefore control a larger amount of functionality.

Here, we provide algorithms and databases that answer the following questions: What do unknown TFBMs look like? Given all possible TFBMs, what is the motif combinatorial binding topology of each promoter? What genes have common TF combinatorial binding patterns and are therefore likely to be switched simultaneously? Do genes regulated by the same TFs have common functions?

In order to answer such questions, we have developed new computational methods that acquire knowledge from the already known TFBSs. The novelty of these methods includes an adaptive choice of the number of aligned sequences from different species using a permutation prefix method, clustering of the selected sequences using a new distance function (conglomerative distance) to gain granularity, and an adaptive choice of the number of features to be learn from each known TFBM thanks to a new filtering technique that we term dynamic dimension selection (DDS). These methods generate a database with the following major features. Firstly, the database provides a dictionary of “DNA words” that contains all the possible binding sites. This dictionary is not associated to any particular cell type but is a key element for inferring the transcription regulations on genomic scale. Such types of dictionaries are a key tool of the techniques that break cryptographic codes [9]. Secondly, our algorithm generates a list of *ab initio* TFBM predictions that cover the unknown 80%–90% of human TFBMs. Thirdly, our method distills a list of common TFBM combinatorial “syntax” rules that arise in the gene promoters region. Fourthly, a sublist of TFBMs, based on motifs that appear in the discovered combinatorial syntax rules, can have a greater ability to regulate larger modules of cellular functionality. Finally, we predict the potential biological functionality of the newly found TFBMs and their combinatorial patterns annotating them with the gene ontology enrichment analysis of their associated gene targets.

The task of finding *ab initio* TFBMs has been tackled in the past with a large number of algorithms. A recent survey [10] states that even after a considerable effort, DNA motif finding still remains an open problem as motif finding algorithms are not able to detect motifs in mammals. Here we describe the two main categories of motif finding algorithms.

### Constrained discovery algorithms

The first category is composed of algorithms [10] that work on small sequence fragments. Initially developed for predicting TFBMs from co-expressed gene clusters determined by transcriptomics experiments, and also used for searching TFBMS in the DNA fragments generated by wet-lab analyses such as DNAse footprinting assay, Electrophoretic Mobility Shift Assay (EMSA) and more recently ChIP-Chip and ChIP-Seq [11]. After sequence mapping, such experiments deliver *loci* of limited length and each of these *loci* is assume to hold a binding site with a certain probability. The algorithms operate on the sequences finding common motifs. Examples of these techniques include AlignAce [12], Gibbs Motif Sampler [13], MEME [14], PhyloGibbs [15] or Weeder [16]. We term them constrained algorithms since they are designed to find only one motif or a small set of motifs from an experiment. Their outcome is cell type specific and they do not extrapolate the knowledge from known motifs to perform *ab initio* predictions. Elemento and Tavazoie [17] argued that such techniques are not appropriate, primarily because binding site density is low. They also mentioned that those algorithms are relatively slow and can miss many regulatory elements as they are based on stochastic methods [18]–[21]. In practice, such approaches are reported to work only on small genomes like yeast [10]. The described problems render these algorithms ineffective as the genome becomes more complex [10]. Our objective is to discover from whole eukaryote genomes *ab initio* cell type independent motifs using computer learning techniques that take advantage of the intrinsic binding properties of the known motifs.

### Unconstrained discovery algorithms

The second category comprises algorithms that systematically discover *ab initio* motifs [17], [22]–[25]. These algorithms rely on the small number of known TFBMs compared to the number of TFs. They try to generalize the properties of the already known TFBMs to find new TFBMs. This is a challenging task since the number of binding sites targeted by TFBMs is very small compared to the number of all the possible subsequences of a genome. Table 1 shows a list of whole genome motif discovery methods and their performance characteristics. Success rates vary widely among different algorithms. In the yeast case, better results were achieved [26] due to the lower genome complexity in this organism. Since previous methods have been tested with different motif discovery conditions, the number of validations varies considerably. E.g., in the human, Elemento *et al.* [24] employed 309 validation motifs whereas Xie *et al.* [22] used only 123. Regarding the number of *ab initio* predictions, in human, FastCompare [24] produced 284 novel predictions whereas the method proposed by Xie *et al.* [22] provided 184. These numbers are still far from the final goal of 1400–2600 TFBMs. Furthermore, all methods besides the one presented in this publication, employ IUPAC (International Union of Pure and Applied Chemistry) [27] symbols to describe motifs. This is a drawback since the generated motifs are too coarse, and therefore their biological accuracy is reduced. None of the previous methods employs machine learning techniques to find properties intrinsic to known TFBMs. A common trend is to employ one-dimensional overrepresentation/conservation scores [22], but this is a very limiting approach to describe complex binding patterns. Finally, TF combinatorial co-occurrence that is a valuable filter to find TFs controlling large modules of functionality in the cell, is not performed by previous approaches.

**Table 1:** Comparison of unconstrained motif discovery algorithms.

Method	Year	Species	Succ.	Test	Novel	ML	Comb.	HR
Kellis *et al.* [26]	2003	*Sc*	65%	55	72	N	N	N
FastCompare[24]	2005	*Sc*; *Sb*	8%	309	398	N	N	N
FastCompare[24]	2005	*Ce*; *Cb*	4%	309	437	N	N	N
FastCompare[24]	2005	*Hs*; *Mm*	5%	309	284	N	N	N
Xie *et al.* [22]	2005	*Hs*	56%	123	174	N	N	N
Stark *et al.* [68]	2007	*Dm*	46%	87	145	N	N	N
MDOS[69]	2008	*Pf*	20%	30	26	N	N	N
Kumar *et al.* [23]	2010	*Fg*	21%	76	108	N	N	N
This publication	2012	*Hs*	81%	509	4098	Y	Y	Y

The method and the year of publication is displayed along the species. “Succ.” is the success rate of the algorithm or the percentage of known motifs rediscovered. “Test” is the number of known motifs tested in the validation step. “Novel” is the number of novel motifs predicted. “ML” indicates whether a machine learning approach is used. “Comb.” indicates whether a combinatorial filter is employed. “HR” indicates whether high resolution predictions are available. If IUPAC symbols are used during the enumeration phase, the prediction resolution is low. Species: *Cb* = *Caenorhabditis briggsae*; *Ce* = *Caenorhabditis elegans*; *Dm* = *Drosophila melanogaster*; *Fg* = *Fusarium graminearum*; *Hs* = *Homo sapiens*; *Mm* = *Mus musculus*; *Pf* = *Plasmodium falciparum*; *Sb* = *Saccharomyces bayanus*; *Sc* = *Saccharomyces cerevisiae*.

Our algorithm significantly improves the state of the art by fundamentally changing the way to tackle the problem. First, we add features intrinsic to known TFBMs (see Table 2 ) that are processed with a machine learning approach. This allows our algorithm to focus not only on overrepresented and overconserved elements but also on elements that match closely existing TFBMs in a hyperdimensional space. TFs are projected onto multidimensional vectors, and we attempt to find novel motifs that are in the vicinity of existing points (known TFs). Second, our system does not employ IUPAC symbolic simplification to generate motifs. Instead, it takes advantage of the richer information carried by the collection of binding site sequences targeted by each TF. Third, it uses similarity search to create more natural and biologically meaningful motifs with very high Pearson correlation with already known TFBMs. Fourth, our algorithm employs a combinatorial filter that finds motifs that are common in multiple gene promoters in a predictable topological form. This allowed us to obtain the best results with the largest validation set, the highest number of novel predictions, and the highest success rate reported so far in the literature for unconstrained whole genome motif discovery algorithms.

**Table 2:** Intrinsic properties of the TFBMs used by our algorithms.

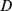	Feature	Description
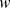	*Entropy curve*	Entropy-based fingerprint
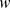	*Distance distribution*	Inter binding site Hamming distance distribution of the prediction
1	*Conservation score*	Conservation score from Xie *et al.* [22]
1	*Number of conserved sites*	Total number of conserved sites for each prediction
1	*Conservation per base*	Average number of conserved species per base for each prediction site
1	*Entropy per base*	Average motif entropy per base

For each dictionary of “DNA words” of length 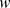, the algorithm works in a (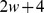) dimensional space defined by six multidimensional feature fingerprints. “
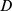
” is the number of feature dimensions.

## Results

### Prediction of 4089 new transcription factor binding motifs and validation against known motifs

**Figure 1:**
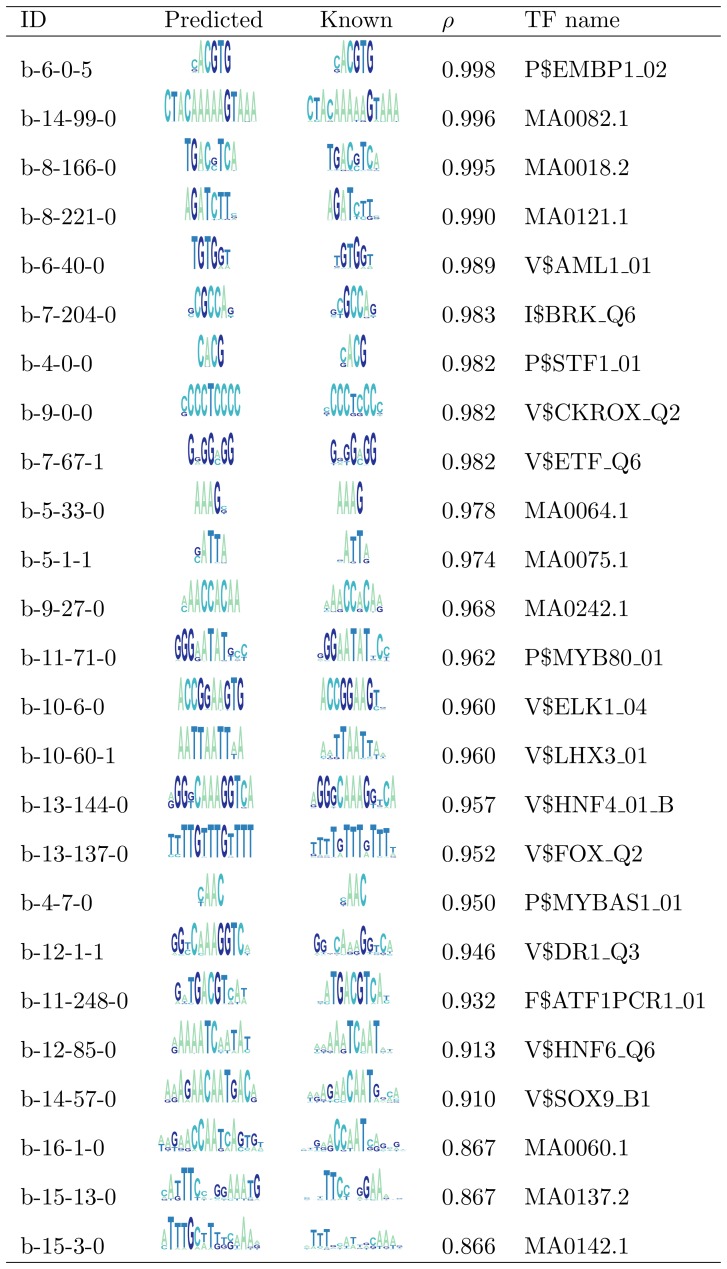
Comparison of predicted motifs against known regulatory motifs. “ID” is the TFBM identification string and 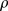 is the Pearson correlation of the similarity between the predicted and the known TFBMs. The TF names starting with MA correspond to Jaspar TFBM identifiers, the names with a $ symbol correspond to Transfac identifiers.

To disclose *ab initio* TFBMs on genomic scale, we designed a pipeline of algorithms that decrease progressively the amount of genomic data to be processed. First, we compiled “DNA word” dictionaries from all the gene promoters of 5000 base pairs upstream the transcription start site of the human genome. We designed three new filters to reduce the number of “DNA words” in the dictionaries. The reduction achieved by each filter is shown in Table S1 in Supporting Information S1. The parameters employed for the dictionaries generation are shown in Figure S1 in Supporting Information S1, and examples of how such filters work are shown in Tables S2 and S3 in Supporting Information S1. The “DNA word” reduction achieved by these filters facilitates at a second stage, to complete the TFBM map through clustering. To decrease the potential TFBM candidates to an amenable number, we created a novel Dynamic Dimension Selection (DDS) filter, that using the parameters depicted in Table S4 in Supporting Information S1 dramatically condenses the number of potential TFBM as shown in Table S5 in Supporting Information S1. A final merging step identifies 4598 motifs. This number is higher than the estimated upper bound of 2600 TFs [3], but one has to consider that a TF can use several binding modes which can generate the so called secondary motifs [6]. Using as a similarity criterion a Pearson correlation of at least 0.85, our predictions matched 83% known TFs from Jaspar [4] (228 TFBMs) and 81% of Transfac [5] (281 TFBMs) datasets. To achieve these results, for each motif of length 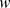, 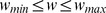 (where 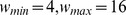) we trained our algorithms with a subset of known TFBMs with a length different from 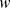. Then we predicted the existence of the trained TFBMs in the disjoint validation subset composed by the TFBMs of length 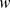. Once known motifs were removed, there remained 4089 novel predictions. To verify that our predictions do not occur by chance, we measured the percentage of predictions that match a random control TFBM dataset. Since each TFBM is characterized by a position weight matrix (PWM) [28], to generate a dataset without nucleotide compositional biases, the elements of each of the Jaspar and Transfac PWMs were shuffled for each column while keeping the column order. We calculated how many of our predictions had a similarity higher than a threshold against at least one of the random PWMs. We selected the similarity threshold following [22], considering that a prediction matches a known TFBM when the Pearson correlation of its respective PWMs is 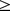
0.85. We found that only 1% of our predictions matched the random dataset, thus, the probability that our *ab initio* TFBMs have been generated by chance is low. Figure 1 presents the known motifs predicted by our method with the highest two matches obtained for each prediction of a specific length. The entire list of predictions is shown in the Table S6 in Supporting Information S1. Our algorithm is able to predict not only individual TFBMs but also possible combinations, such as the OCT4+SOX2 (MA0142.1) pair, which is well known in embryonic stem cells (ESCs) [8], [29]. In ESCs, OCT4 activates downstream genes by binding to enhancers carrying the octamer-sox motif (OCT-SOX enhancer) for synergistic activation with SOX2 [30], playing a key role in the maintenance of pluripotency [31]–[33]. The pair OCT4+SOX2 is also crucial for cellular reprogramming, since both OCT4 and SOX2 are members (together with KLF4 and MYC) of the Yamanaka reprogramming cocktail [34], and (together with LIN28 and NANOG) of the Thomson reprogramming cocktail [35]. The ability to discover OCT4+SOX2 shows possible follow up applications of our algorithm for understanding the reprogramming mechanisms. When comparing with other unconstrained discovery algorithms, we found that though our method was validated with the largest amount of known TFBMs (509), it still achieved the highest success rate (81%) reported in the literature (see Table 1 ).

### The TFBMs emerge forming combinatorial binding patterns

Since the TFBMs are short, they do not have enough specificity, and there are multiple evidences that TFs work together in combinatorial assemblies, such as the OCT4+SOX2 partnership [30]. We hypothesized that when a motif appears together with the same set of motifs in multiple promoters, the motif is more likely to play a relevant role in gene transcription regulation. We denote this arrangement of TFBM co-occurrence as combinatorial binding patterns (CBPs), i.e., CBPs are “motifs of motifs”. To discover such patterns, we developed a computational method that identifies combinations of motifs that bind to more than one promoter. The 17831 CBPs we found cover 73% of already known TF-TF binary interactions listed in the Transcompel [5] dataset. The best CBP match against each Transcompel entry is shown in the Table S7 in Supporting Information S1.

**Figure 2:**
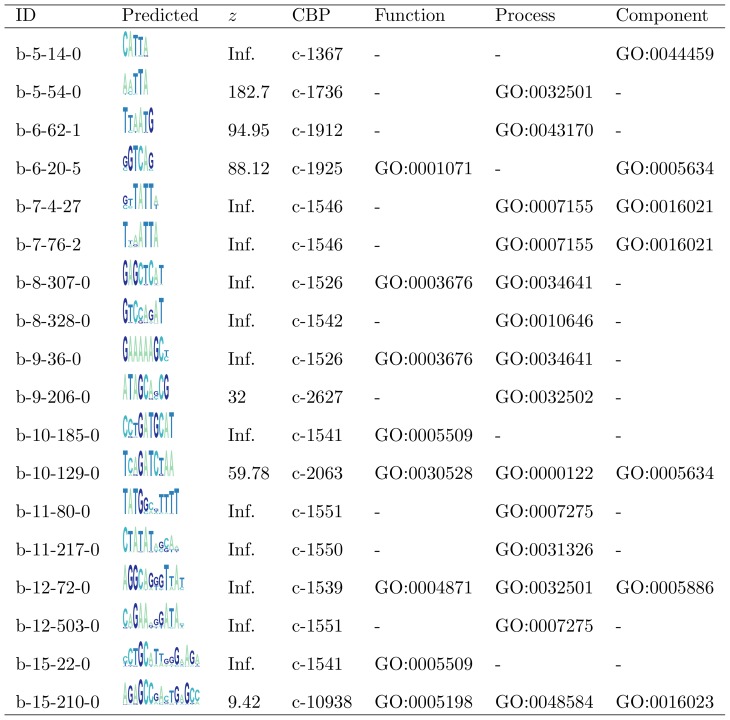
Novel predicted motifs that appear frequently in CBPs (STFBM). The position within this subset is included along the motif ID, the motif logo, the significance 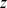 score, the corresponding CBP ID. Additionally, the most significant molecular function, biological process and cellular component ontologies are included for each motif.

From the initial set of TFBM predictions, we found a subset of 504 motifs that also co-occur with other motifs in multiple gene promoters. This set is denoted as STFBM (significant TFBM). Figure 2 shows the top two matches for each motif prediction set of length 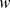 that include statistically significant gene ontology (GO) enrichment over the genes targeted by the CBPs. The complete STFBM is presented in the Table S8 in Supporting Information S1. By applying the GO enrichment analysis over the genes targeted by such motifs (section S1.5 in Supporting Information S1), we annotated successfully 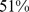 of STFBMs with GO terms with statistical significance (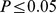), and found that this subset is constituted exceptionally by TFs and enriched with pattern formation and morphogenesis related GO terms (section S2 .1 in Supporting Information S1).

**Figure 3:**
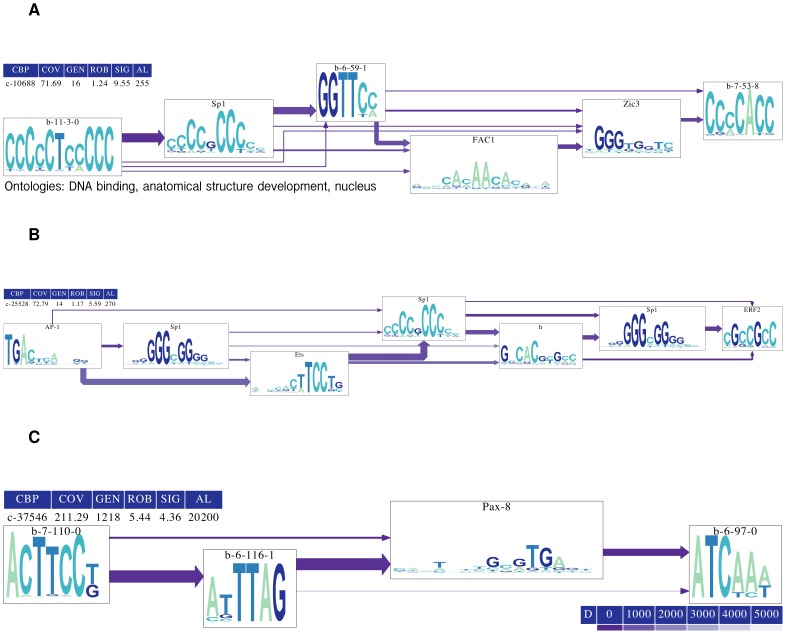
Visualization of discovered CBPs. Visualization of CBPs with (A) , (B) low and (C) high number of target genes. Each arrow connecting two motifs 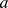 and 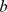 implies that the motif 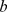 is to the “right” of the motif 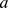. The arrow thickness reflects the percentage of promoters where the motif relationship occurs. The gradient “D” is the number of base pairs separating each binding site. “CBP” is the unique identifier for each discovered pattern. “COV” (coverage) is the average number of base pairs available for binding. “GEN” (generality) is the number of genes targeted by the pattern. “ROB” (robustness) is the average number of times a motif is repeated within the pattern. “SIG” (significance) is the 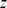-score of the actual GEN in the distribution of GEN that would be generated if the promoters were randomly shuffled. “AL” is the motif multiple alignment score. Higher values of “AL” represent better alignment.

To visualize the topology of the CBPs, we designed a technique (see section S1.5.1 in Supporting Information S1) based on performing multiple alignments of the predicted TFBS *loci* coordinates. Figure 3 gives three examples of this visualization. Figure 3A shows a very specific CBP that applies to 16 promoters. Our *ab initio* TFBM predictions “b-11-3-0” and “b-7-53-8” are mixed with the TFs Sp1, FAC1, and Zic3. Gene expression microarrays [36] have already shown that Sp1 and Zic3 are members of one gene regulation cluster in cardiac cells (cluster 7), whereas FAC1 and Zic3 belong to clusters 22, 63, and 101. Finally, Sp1 and FAC1 belong to cluster 72. Figure 3 B shows a CBP that involves AP-1/Sp1 interactions. This interaction has already been documented [37] and is related to 12-*O*-tetradecanoylphorbol-13-acetate (TPA) response in keratinocytes. This example shows the ability of our system to find co-occurrence of the same motif in different directions (see the multiple appearances of the Sp1 complement). Finally, Figure 3 C displays a comprehensive CBP that applies to 1218 genes. The TF Pax-8 is surrounded by three of our predictions. The CBP subset with the strongest connection is composed of “b-7-110-0”, “b-6-116-1”, and “Pax-8”. As PAX-8 is an excellent marker for primary tumor sites [38], our *ab initio* TFBMs associated with Pax-8 could provide insights into tumor formation regulation. We found 9 additional CBPs that apply to more than 1000 genes (see CBP gallery in Table S9 in Supporting Information S1), indicating that a small subset of CBPs has the potential to exert regulation over large sections of the genome.

### The intrinsic specificity of the CBPs is limited to subsets of ten or more genes

**Figure 4:**
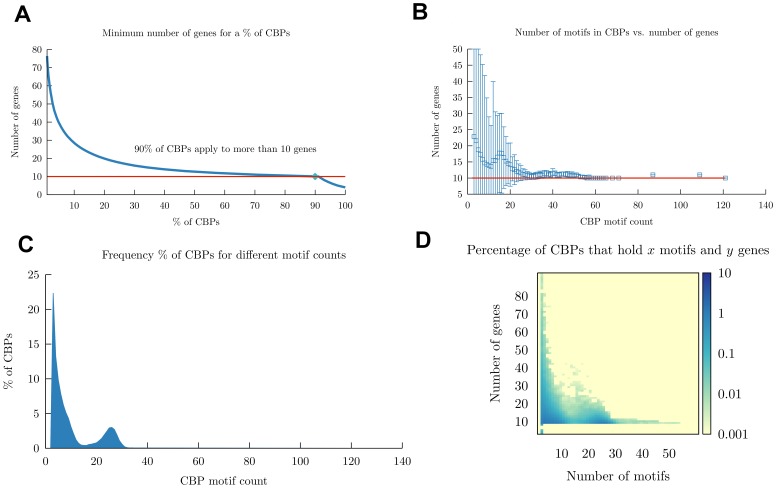
CBPs apply to 10 or more genes. (A) Cumulative percentage of CBPs (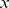 axis) that apply to a given number of genes (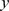 axis). (B) Relationship between CBP motif count and gene count. The 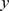 axis represents the average number of genes contained in the CBPs. One standard deviation surrounds each point. The 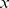 axis represents all the CBPs with a certain number of motifs. The horizontal red line marks the 10 gene boundary. (C) Frequency of CBPs for different motif counts. The 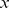 axis represents the motif count for all the CBPs. The 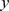 axis represents the percentage of CBPs that hold the given size. (D) Percentage of CBPs that apply to a given number of genes and motifs.

To study to which degree the CBP are specific to genome *loci*, we analyzed the distribution of the gene targets covered by each CBP. Figure 4 A depicts the cumulative percentage of CBPs that apply to a certain number of genes and shows an elbow at the level of 10 genes. Figure 4 B displays the number of genes versus the CBPs of a specific motif count showing that the average number of genes switched by CBPs is always equal to or greater than 10. These results indicate that, on average, 90% of those CBPs are shared among 10 different genes. The analysis of the average number of genes covered by CBPs with a specific amount of motifs shows that as the number of motif count increases (Figure 4 C), the gene count reduces and stabilizes at about 30 motifs. Few CBPs hold more than 30 motifs. Such observation is highlighted by the heat map in Figure 4 D, showing that the percentage of CBPs that holds 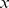 motifs and 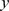 genes has a high density region in the area of 10 genes targeted by less than 30 motifs. These results reveal that the newly discovered arrangements of TFBMs into CBPs increase the binding specificity only to an extent that a CBP can switch 10 genes on average. Thus, the CBPs exert clusters with a granularity of more than 9 genes. This suggests that the simultaneous switching of fewer than 10 genes requires other mechanisms besides TF combinatorial binding. These computational results are in agreement with the findings of Lieberman-Aiden *et al.* [39], who coupled proximity-based ligation with massively parallel sequencing (Hi-C) and with the gene expression microarray results of Vogel *et al.* [36]. The former showed that chromatin segregates into two genome-wide compartments, where the open one is consistent with a knot-free fractal globule that preserves the ability to unfold any genomic locus. The latter showed that a large proportion of the human transcriptome is organized into gene clusters that are partially regulated by the same TFs.

### The *ab initio* predicted TFBS and CBPs are related with developmental functions and transcription regulation processes

**Figure 5:**
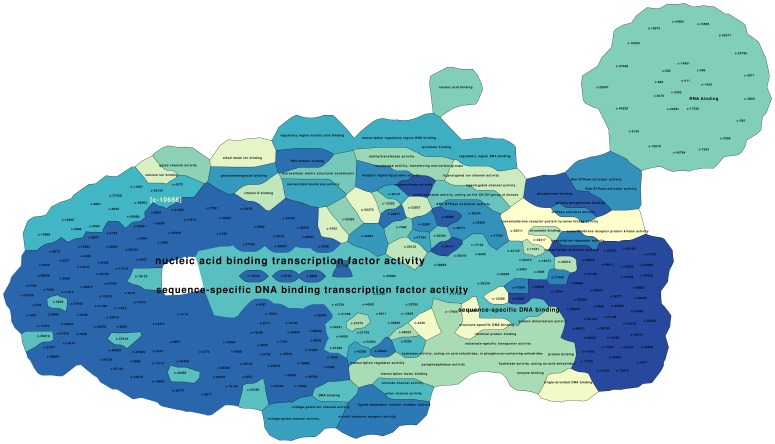
Map of significant CBP molecular function ontologies. Regions with the same color represent the same cluster of objects. Borders of the “countries” underline boundaries of CBP clusters. The ontology term font size is adjusted to reflect the frequency with which it is associated to the CBPs. The CBP c-10688 has been enhanced for readability in the upper left corner of the visualization map.

**Figure 6:**
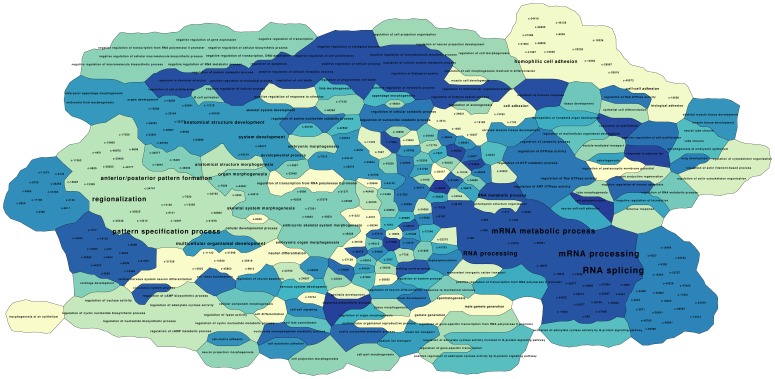
Map of the significant CBP biological process ontologies. Regions with the same color represent the same cluster of objects. Borders of the “countries” underline boundaries of CBP clusters. The ontology term font size is adjusted to reflect the frequency with which it is associated to the CBPs.

To reveal the possible biological meaning of the newly discovered TFBMs and CBPs, we generated a list of significantly enriched GO terms associated with the genes targeted by the motifs and CBPs. We were able to annotate with a statistical significant enrichment the GO terms of 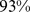 of the predicted CBPs and 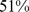 of the TFBMs, which shows that the newly discovered TFBMs and CBPs are likely to be biologically meaningful. We developed the concept of “ontology maps” to visualize the relationship between GO terms and TFBMs or CBPs (section S1.6.2 in Supporting Information S1) with “graphs as maps” (GMAP) [40]. Figure 5 presents the map of CBP molecular function ontologies. All elements enclosed in the same “country” of the map have links to similar sets of ontologies and therefore cluster together. The visualization shows that the surrounding CBPs also bind to genes exhibiting similar ontologies. The text size reflects the most common, significant GO terms. Interestingly, we found that such terms are related to molecular function ontologies of TFs revealing a cascade of TF binding events, i.e. the CBPs we discovered have a trend to bind to the regulatory region of other TFs. As an example of TF activity, the white highlight in the upper left region of Figure 5 (near the nucleic acid binding TF activity “country”) marks the position of the c-10688 CBP, whose CBP arrangement is depicted in Figure 3 A. Additionally, the upper right corner of Figure 5 depicts a cluster of CBPs connected strongly with the “RNA binding” ontology. The corresponding “RNA splicing” term is found to be a biological process significantly regulated by CBPs, as shown in Figure 6. This correspondence suggests that the modular nature of the protein blocks codified in sets of exons to generate different isoforms could require the concurrence of a large number of TFs. Figure S6 in Supporting Information S1 depicts the ontology map of CBPs cellular components. We also found a significant enrichment in the TFBMs GO analysis of transcription regulation related terms such as “transcription factor regulator activity” and “transcription factor binding”, and of developmental related terms such as “developmental process”, “anatomical structure development”, “tissue development” and “organ morphogenesis” (see Figures S7, S8 and S9 in Supporting Information S1).

## Discussion

We have created novel proximity algorithms that infer a catalog of human regulatory motifs and their combinatorial binding patterns from previously known TFs. Our methods predicted 81%–83% of the known TF binding motifs and discovered 4089 novel TFBMs. This is the highest success rate achieved so far in mammalian whole genome TFBM discovery and it is the most widely validated method with rediscovering 509 known motifs of the Jaspar and Transfac databases. This success rate shows that our machine learning approach is useful to improve the predictions quality. Since our algorithm is not based on IUPAC strings, it creates smoother motifs that closely match experimentally discovered TFBMs than the methods based on IUPAC representations.

A combinatorial pattern filter selected a subset of 504 motifs that co-occur frequently with other motifs in 17831 CBPs. These motifs hold co-occurrence patterns that span hundreds of genes. A high percentage of our CBP and TFBM predictions can be annotated with statistical significance with GO terms. Such annotation reveals that our CBP and TFBM predictions are strongly related to transcription activity and development. Thus, the TFs show a trend to target other TFs. This additional level of regulation has been discovered in several specific cases such as the Microphthalmia-associated transcription factor (MITF) regulation by SOX-10 and PAX-3 in the Waardenburg syndrome [41], the modulation of chondrogenesis onset by Runx [42], the specification of lymphoid cell fates [43], or the ESC regulatory circuitry [44], [45]. We found that such property seemed to be an intrinsic genomic sequence hallmark with expanding influence on genomic scale. In our study, genes involved in pattern formation, morphogenesis and development were also regulated by our *ab initio* TFBMs and CBPs with high significance. This identifies development as one of the most demanding regulatory processes, in agreement with the pivotal role of transcription regulation during development [46].

The discovered TFBMs and CBPs hold properties that appear to be intrinsic to the genome, as they have been revealed using a method that applies the same algorithm over all promoter regions. Potential features encoded in the genome confer additional regulatory capabilities onto the cell, the degree of which may depend on the particular cell type. Thus, gene regulation at the TF level appears to be focused on the regulation of other TFs influencing the development of the organism. Accordingly, the development of an organism could entail cellular functions whose regulation requires higher levels of complexity.

The predicted CBPs are generated greedily in an attempt to extract potential syntactic patterns that are as general as possible. We have created a computational approach to stretch general genomic syntactic rules as much as possible, with as many motifs as possible. Even though we accepted a correct CBP as a syntactic rule that applies to at least two genes, we discovered a consistent and surprising trend to generate sets of rules that apply to 10 or more genes.

Previous unconstrained motif discovery approaches [22], generate less specific motifs as more species are added, and their sensitivity analysis showed that their method predicted with less accuracy (69 hits out of 123 tested motifs). Instead, our method takes advantage of additional information provided by the growing number of aligned species in databases such as the UCSC genome browser [47]. It has the capability to improve its performance as more genomic data becomes available, because we employed a permutation technique whose key feature is to select adaptively the number of closer species to focus on relevant matches only (see Materials and methods section).

Our method is designed to learn from some additional TF intrinsic properties (Table 2 ) that profit from the extended sequence alignments. These properties do not require sequence overrepresentation and focus on the fingerprints of specific sequences. Thus, our algorithm searches for TFBMs that do not necessarily occur frequently in the genome. Our usage of TF intrinsic properties allows considerable reduction of the input noise (see section S1.4 in Supporting Information S1). Our “DNA word” dictionary compilation algorithm adapts locally to evolution changes. Besides evolution, the multiple alignment heuristic [48] employed may also introduce artifacts into the data. Since our method learns from the feature space, better results have been achieved.

We hypothesized that evolution occurs at different rates along the genome. Therefore, local genomic regions may have different conservation levels among species [49]. Such difference may not be considered in a general phylogenetic tree based on whole genome sequences. Other methods take such a generalistic approach [23], but the adaptive selection of aligned sequences provided by our permutation prefix approach for filtering “DNA words” makes our algorithm more flexible by adapting locally, depending on learned features. Permutation prefixes have been used recently as fingerprints in the field of similarity search [50]–[53].

The DDS algorithm introduced a novel way to handle high dimensional data by focusing on certain features of the space. This allowed us to project each prediction into a high dimensional space without the need to evaluate the relevance of different features. The important features are found at classification time and depend on the target object. By concentrating on a limited subset of components, the DDS algorithm removes noise and focuses on the important features. The dimension selection is dynamic and depends on each object, and unlike the Euclidean metric, other 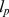 metrics, and some recent approaches [54], [55], the differences of the components are not mixed when the similarity is calculated. Since the DDS treats each single component independently, there is no need to normalize data with different magnitudes. The combination of these key properties allowed the DDS to remove vast amounts of spurious noise (see Table S5 in Supporting Information S1), thus, reducing the number of false positives.

With our algorithms we generated a catalog of predictions that contains the “DNA words” dictionary, the motif predictions, the binding site location for each known and novel motifs, the CBPs predictions, and the visualizations for motifs and CBPs which can be downloaded from http://computational-biology.mpi-muenster.mpg.de/publications/TFBM/.

## Materials and Methods

**Figure 7:**
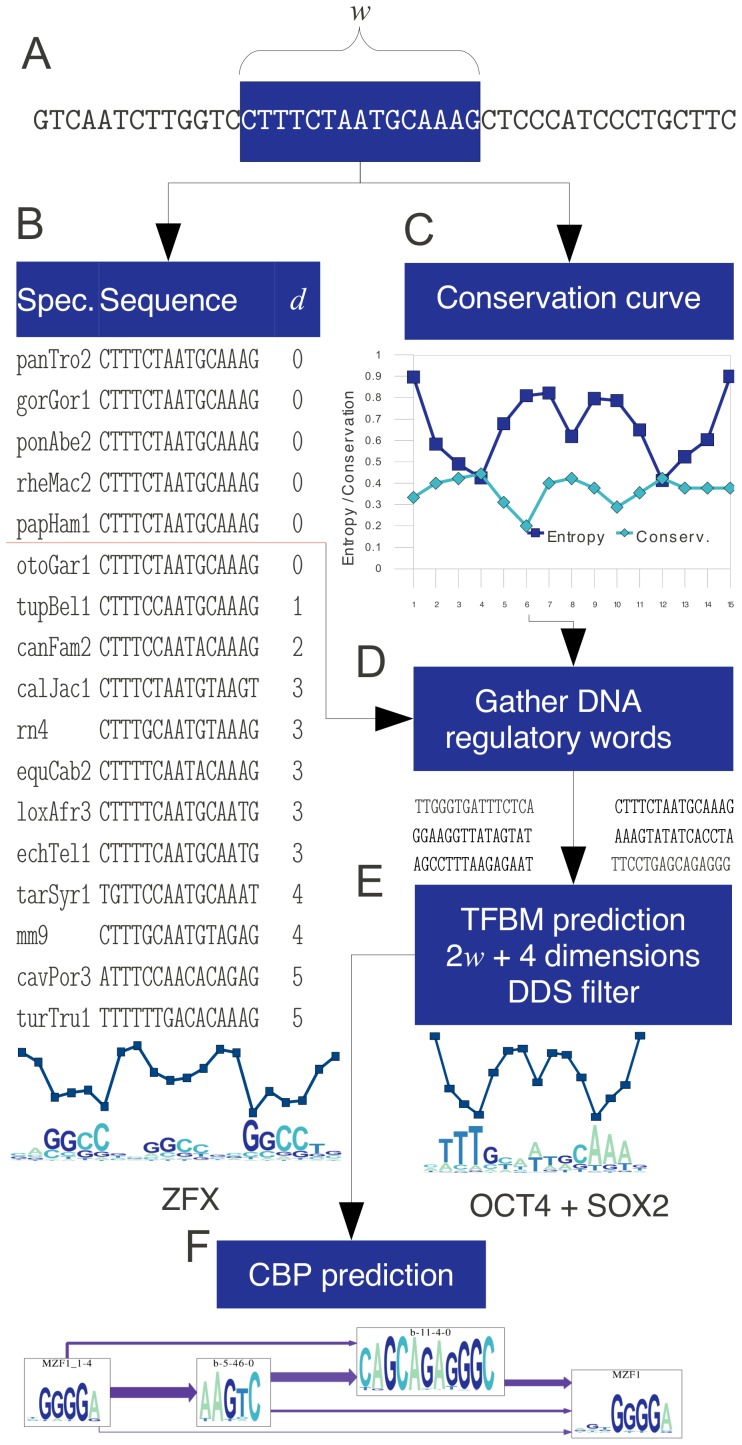
Method overview. (A) Extract “DNA words” of length 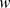. (B) Filter the “DNA words” with the permutation prefix method, by ordering the aligned sequences of different specie genomes (Spec.) by the closeness 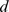 to the target sequence. The horizontal red line marks the end of the species permutation prefix. (C) Reduce the number of “DNA words” by the conservation curve. (D) Gather the intersection of “DNA words” generated in (B) and (C) with the merging method. (E) Predict the TFBM candidates using the DDS filter over a projection of (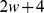) features (see Figure 8). (F) Take the predicted TFBMs to generate the catalog of combinatorial binding patterns of all the discovered motifs.

Our method for TFBM discovery first creates a dictionary of “DNA words” with high probability to be bound by TFs is employed to predict binding motifs using a new clustering method. Once binding motifs are predicted, the algorithm finds TF combinatorial binding patterns (CBPs). Figure 7 is an overview of our computational pipeline.

### Compilation of “DNA word” dictionaries

The first step is to create a dictionary of DNA sequences of length 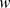, with 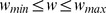 (where 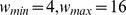), that includes potential binding sites for all the TFs. To compile this dictionary, our algorithm learns intrinsic properties (see Table 2 ) of known TFBSs and then generates a universal list of “DNA words” that are likely to be a TFBS. The input of the method is the multiple alignment format (MAF) dataset of 45 species provided by the UCSC genome browser [47], and known TFBMs from Jaspar [4] and Transfac [5] that are used as learning/validation data. To reduce the number of “DNA words”, and thus, to decrease the computational burden we designed three filtering techniques, the conservation curve, the permutation prefix, and a merging method.

The first filtering method is based on the conservation curve (Figure 7 C), is employed in order to reduce the number of “DNA word” candidates. Note how the entropy inversely matches the conservation curve in this example. This entropy/conservation relationship does not necessarily have always to match, but it does reveal that the shape of the conservation profile carries an important intrinsic property of the TFBS.

The second filtering method is based on similarity permutations. For each “DNA word” of length 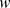 (Figure 7 A), we employ the order of similarity between the aligned sequences of 45 different species and the “DNA word” that is to be analyzed. This order creates a permutation. The similarity criteria used to create the permutations is based on the Hamming distance [56] (defined as the number of positions 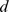 that differ between two sequences). This permutation of species sequences is a valuable fingerprint to prune the search space as shown in Figure 7 B. The compilation dictionary algorithm keeps track of the closest species of the known binding sites, and uses them as learning data. Since the species order changes depending on the similarity of the aligned sequences, such alignment creates a permutation. The permutations that belong to known TFBSs become the learning set. Then, it is possible to filter each site only when the permutation is present in the learned set. Since a permutation of 45 elements will likely be unique for each site in the genome, we take as a prefix the first 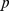 closest species, that is smaller than the total number of species. The prefix value 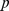 was set to 5 for DNA dictionaries greater than 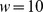 bases (for 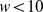 see Figure S1 B) in Supporting Information S1. In Figure 7 B, the red line represents the permutation prefix in the example.

The “DNA word” extraction process shown in Figures 7 B and 7 C creates two different sets of “DNA words”. We take the intersection of them to feed the TFBM prediction algorithm using the merging method (Figure 7 D). This set intersection of “DNA words” is analogous to a universal dictionary of potential TF binding targets. Table S1 in Supporting Information S1 shows the resulting sizes for each dictionary generated by the conservation curve method, the permutation prefix method and the merging method. This reduction is crucial for increasing the quality of the results and for making it computationally feasible to generate the motif clustering. Once this dictionary is created, we proceed to predict TFBMs using clustering techniques (Figure 7 E).

### Completion of the TFBM map through clustering

Once obtained a set 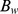 of “DNA words” of potential regulatory meaning, the next step is to extract biologically meaningful motifs. We expanded previous work on the subject of motif prediction [22]–[25] to integrate with a machine learning approach, not only comparative genomics, but also intrinsic TF properties. We assume that each “DNA word” in 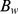 is the center of a cluster of size 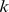. This cluster is generated by obtaining the 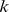 closest (measured with a conglomerate distance) elements in 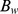. The algorithm find the subset of elements that create an entropy curve that matches better the entropy curves generated from the training set 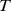. Each cluster is ranked according to a quality criteria based on phylogenetic conservation and on average entropy. Then the predictions that do not follow the general patterns of motifs are eliminated using the new developed DDS filter. Finally, the clusters that share similar motifs are removed and the similar clusters are grouped.

#### Binding site cluster creation

**Figure 8:**
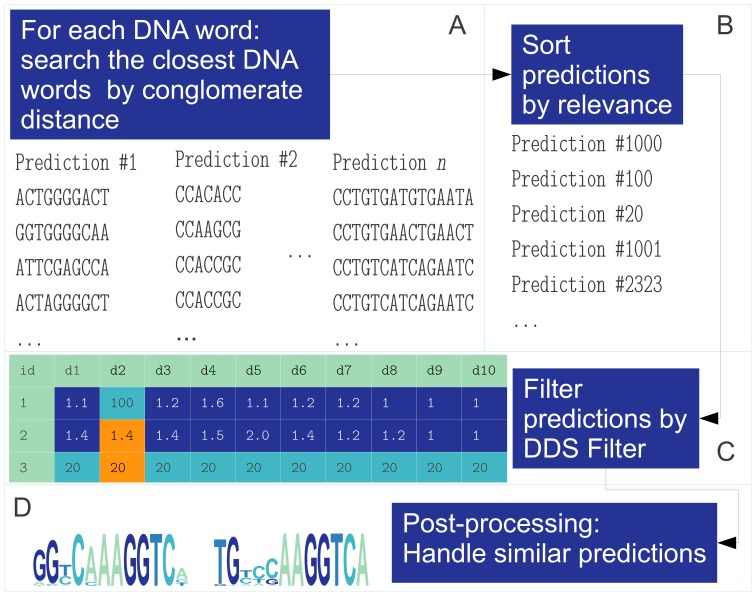
TFBM prediction method. (A) Cluster “DNA words” of length 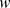 by the conglomerate distance. A greedy algorithm removes sites that do not keep the motif within a certain distance of the learning set. (B) Sort predictions by the phylogenetic score given by Eqs. 2 and 3. (C) Filter those predictions that are far away from the learning set in the projected space of (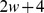) features. An example of how the DDS filter works is represented in the table with three vectors of 10 components each. (D) Post-process similar predictions by grouping them if they are close (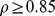), and an example of two similar grouped predictions.

Our TFBM discovery method is based on the search for signature “DNA words” using a similarity search strategy [57] using a novel sequence distance approach that we term “conglomerate distance”. The idea behind this metric is to consider in the distance computation, the groups of identical sub-sequences shared between two sequences, providing extra weight to clusters of fragmented sub-sequences. Given two sequences 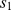 and 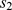 of length 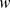 we define its “conglomerate distance” as
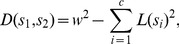
(1)where 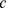 is the number of identical sub-sequences 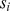 between 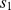 and 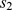, and 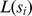 is the length of each of such sub-sequences. The conglomerate distance is smaller when a larger number of contiguous bases or “chunks” is equal. It provides higher granularity than the Hamming distance and is specially useful for large sequence lengths. Thus, we have obtained better results with the “conglomerate distance” than with the Hamming distance. For a detailed explanation of the “conglomerate distance” see section S1.4.2 in Supporting Information S1 and pseudo-code in Figure S2 in Supporting Information S1. For each “DNA word” we take the top 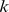 closest “DNA words” based on the conglomerate distance (Eq. 1) and create an initial TFBM prediction. We refine the “DNA words” with a greedy filtering algorithm (see section S1.4.2 and pseudo-code in Figure S3 in Supporting Information S1), so that the entropy profiles match better known entropy profiles of other motifs. Figure 8 A is an example of this clustering. Each “DNA word” cluster becomes a TFBM prediction. We employed recent similarity search techniques [58], [59] that allowed us to perform large scale searches at very high speed to obtain “DNA word” clustering. The algorithm projects each “DNA word” into a low dimensional space of 16 bits. This allows high speed similarity searches and the efficient construction of the nearest neighbors graph [60] of the “DNA word” dictionary.

#### Cluster prediction ranking

Once the clusters are fitted, they are ranked according tro two quality criteria. During the dictionary generation, we keep for each “DNA word” of length 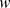, the number of times the word is conserved, 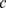, and the number of its appearances, 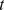. With the recorded 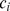 and 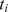 of each site 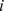 of the cluster, we calculate the phylogenetic conservation 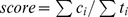 dividing the total number of conserved instances 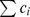 by the total number of instances 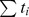. Additionally, to deal with the case of large 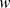 in which sequences appear only once, we define a 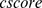, normalizing the total number of appearances divided by the maximum number of appearances 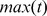. We add both scores to create a ranking value.
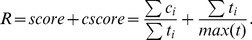
(2)Higher values of 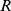 represent a better score. Finally, in the case of equal score, the order is decided by the average entropy:
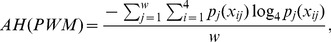
(3)where 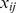 are the elements of the PWM with 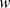 columns, and 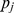 is the probability of occurrence of the base represented by 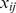 in the column 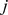. Figure 8 B shows an example of this ranking criterion.

#### Binding site cluster filtering by dynamic dimension selection (DDS)

Once the predictions are sorted by its relevance provided by the ranking criterion (Eqs. 2 and 3), the motifs that emerge naturally are filtered with a novel algorithm called DDS filter (Figure 7 E). This filter is necessary since the intrinsic properties describing the potential TFBM are of a different nature (see Table 2 ). Depending on each TFBM, the weight of their contribution to the cluster generation is different. The DDS adaptively searches which components of the feature space are most appropriate to cluster each potential motif. To determine which motif predictions are more likely to belong to TFs, we look at feature projections of the TFBMs. The filter is a machine learning algorithm that processes as learning data, known TFBMs. Then, it discriminates between predictions that follow intrinsic properties of TFBMs. For each motif of length of 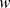, the filter works on the projection of the motif into a feature set of (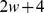) fingerprints. Thelist of feature fingerprints is given in Table 2 .

Since the projected space is of high dimension, the curse of dimensionality [61] makes it difficult to discriminate between motifs, e.g., for 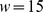, it generates 34-dimensional fingerprint vectors and standard methods like one-class support vector machines [62] do not prune the search space effectively. To solve this filtering problem we developed the DDS filter. Given a 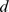-dimensional object query 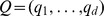, for each component 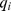 the DDS orders the objects in the database according to their distance to 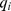. These sorted lists of results 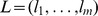 (where 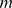 is the number of object in the database) are iterated one list 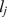 at a time. The DDS records incrementally the object IDs found in each component until it finds an object that appears a minimal number of times or until the search reaches a maximum number of iterations. An additional stage stores as a binary vector the components used to validate the training data. The algorithm also precomputes a binary vector 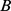 for all the objects in the database (that remains when the object in question is removed) and finds the closest mask in 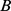 for each query 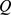. The query is accepted to belong to a cluster if the Hamming distance between the closest vector of the cluster and the query is less than a threshold. The DDS filter and its pseudo-code implementation (Figure S4 in Supporting Information S1) is described in the section S1.4.2 in Supporting Information S1. The key property that DDS filter exploits to remove spurious TFBM predictions is that only a subset of the feature set is relevant to perform the filtering and this subset is different depending on the target object. Figure 8 C shows an example of how the DDS filter works for three vectors of 10 components each. Vector 2 is compared against vectors 1 and 3. The components of vector 2 that are closer to vector 1, are shown in dark blue. and those closer to vector 3 in orange. The Euclidean distance between vectors 1 and 2 is 98.60, and between vectors 2 and 3, 58.98. 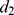 is the only component in which vectors 2 and 3 are closer. If we remove this component, the Euclidean distance between vectors 1 and 2 decreases to 1.01. The final clustering step purges similar predictions (those with more than 80% of shared sites) and clusters with low ranking score. Additionally, those clusters with Pearson correlation 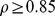 are merged. Figure 8 D depicts two predictions that are combined into a cluster since 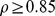. Once the prediction of TFBMs is complete, the output is a list of groups of “DNA word” clusters or TFBMs.

### CBP prediction algorithm

After discovering TFBMs, and their respective binding sites, we uncover the syntax of the crosstalk among them on the genomic scale, estimating the combinatorial binding patterns (CBPs) of the TFBMs. To achieve this, we developed a computational method that finds common “motifs of motifs” based on the similarity of their topological features. The algorithm extracts the promoters that closely resemble an initial “query” promoter. Then, an iterative process links the motifs so that their multiple alignments are as large as possible and target as many genes as possible. We define a “group” as a set of motifs, each separated by not more than 1000 base pairs. The first step of the CBP generation algorithm is to create these groups. For each extracted group, the algorithm starts by searching for groups that share at least 3 motifs. Once this candidate list is made, it is ordered according to the degree of similarity shared with the group. The similarity is the number of shared motifs, and higher similarity is preferred. The algorithm greedily adds candidates. Next, we perform a multiple alignment using the center star algorithm [63]. First, this algorithm finds a center group and uses it to compute pairwise alignment with the other sequences adding spaces as needed. The pairwise alignment is calculated with the Smith Waterman aligner [64]. We set the difference cost to 5, and the open and extended gap penalties to 0. Motifs must align in at least 3 groups to be considered. Thus, we generate CBPs with motifs that are topologically aligned. We explain the pseudo-code (Figure S5 in Supporting Information S1) of the CBP prediction algorithm in detail in the section S1.5 in Supporting Information S1. Those TFBM predictions that occur frequently with others are extracted into CBPs which help us to decipher the common elements required in gene regulation (Figure 7 F). The motifs that appear in the generated CBPs set are extracted as the subset STFBM.

### Ontology analysis and visualizations

We calculated the statistical significance of the ontologies of the list of gene targets associated with all the STFBM and the CBPs generated. To predict the gene targets of each STFBM, we used as in [65] the Berg-von Hippel method [66]. The GO terms were obtained from the AmiGO web server [67]. The statistical significance of the GO terms of each list of genes was analyzed using an enrichment approach based on the hyper-geometric distribution. The GO terms were backpropagated from the final term appearing in the gene annotation to the root term of each ontology. As a background set, we used the list of all the genes in the human genome with annotation on AmiGO. The multitest effect influence was corrected by controlling the false discovery rate, using the Benjamini-Hochberg correction. We developed the “ontology maps” concept to visualize the relationship between TFBMs or CBPs and ontology terms. For two sets of ontologies 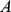 and 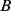, associated to TFBMs or CBPs, we calculated the ontology similarity with the following distance:
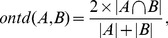
(4)where 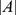 is the cardinality of set 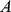. We take the top 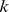 TFBMs or CBPs based on Eq. 4, to establish a topological graph linking those top closest objects. Finally, we create a link between each TFBM or CBP and their correspondent GO terms whose enrichment 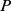-values satisfy a significance level 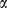. We chose 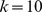 and 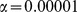 (for cellular component GOs, 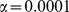). We use the GMAP algorithm [40] to visualize the topological graph.

## Supporting Information

Supporting Information S1 Section S1 of this document contains a more detailed description of the materials and methods; section S2, additional ontology maps, validation results of TFBMs and CBPs, and lists of newly found STFBMs and CBPs; and section S3, a detailed description of the generated database files and their formats. The predicted catalog of regulatory elements can be downloaded from: http://computational-biology.mpi-muenster.mpg.de/publications/TFBM/
(PDF)
