# Supplementary material for: Comprehensive Human Transcription Factor Binding Site Map for Combinatory Binding Motifs Discovery
Source: PLoS One. 2012 Nov 28;7(11):e49086. doi: 10.1371/journal.pone.0049086 (PMC3509107; doi:10.1371/journal.pone.0049086)
Supplement: Supporting Information S1 — Section S1 of this document contains a more detailed description of the materials and methods; section S2, additional ontology maps, validation results of TFBMs and CBPs, and lists of newly found STFBMs and CBPs; and section S3, a detailed description of the generated database files and their formats. The predicted catalog of regulatory elements can be downloaded from: http://computational-biology.mpi-muenster.mpg.de/publications/TFBM/ (PDF) [file pone.0049086.s001.pdf]

Supporting Online Information for the Paper: Comprehensive Human Transcription Factor Binding Site Map for Combinatory Binding Motifs Discovery

Arnoldo J. Müller-Molina,[1] Hans R. Schöler ,[2,3] Marcos J. Araúzo-Bravo[1*]

jn [1]Computational Biology and Bioinformatics Group, Max Planck Institute for Molecular Biomedicine
Röntgenstraße 20, 48149 Münster, Germany
[2]Department of Cell and Developmental Biology, Max Planck Institute for Molecular Biomedicine
Röntgenstraße 20, 48149 Münster, Germany
[3]University of Münster, Medical Faculty, Domagkstraße 3, 48149 Münster, Germany.

[*]To whom correspondence should be addressed; E-mail: marcos.arauzo@mpi-muenster.mpg.de

S1 Supporting materials and methods

S1.1 Data input

We focused on all promoters of the human genome version hg19. We employed the UCSC Genome Browser database upstream5000.maf[1] alignments (*1*). The upstream5000.maf dataset includes multiple alignments of 46 vertebrate species (multiz46way). For training and validation we used already known transcription factor binding sites (TFBSs) from the Jaspar (*2*) and Transfac (*3*) databases. We included only those TFs that have 95% of their sites included in the upstream5000 dataset. Thus, the TF dataset includes:

- From Jaspar (*2*): 228 TFs (each TF has its own version) that comprise 29794 sequences.
- From Transfac (*3*): 281 TFs (version: 2010.1) that comprise 18732 sequences.

The average number of bases per site for Jaspar and Transfac is 11.53. To summarize, our method's inputs are different sets of multiple-aligned sequences SEQ and a set of TFBSs that are used for training and validation. We denote the training set with T and validation one with V. These sets are mutually exclusive.

S1.2 Similarity search

The similarity search, also known as nearest neighbor, proximity search, or the post office problem (*4, 5, 6, 7*), is defined as the search of closest points based on a similarity criteria or a distance function. Let $\mathcal{M} = (\mathcal{D}, d)$ be a metric space for a domain of objects $\mathcal{D}$ and $d : \mathcal{D} \times \mathcal{D} \rightarrow \mathbb{R}$, a total distance function. Given a collection $S \subseteq \mathcal{D}$, we define three types of similarity queries Q:

- k-nearest neighbor (k-NN) query returns the k closest elements to the query object Q. Formally, the set $R \subseteq S$, such that $|R| = k$ and for any $X \in R$ and any $Y \in S - R : d(Q, X) \leq d(Q, Y)$.

[1]Multiple sequence alignments of promoters of 5000 base pairs

- Range query: Given $Q \in \mathcal{D}$ and a range $r \in \mathbb{R}$, it retrieves all the objects within a distance r. Formally, the set $\{X \in S | d(X, Q) \leq r\}$.
- Range k-NN query: the intersection of the previous two types of queries.

With these three types of queries it is possible to obtain a set or cluster of similar objects given an input query object. The k nearest neighbor is a computing burden operation and therefore an indexing data structure is required to reduce the computational burden. We employed a sketch-based index (*8*) implemented in the open source engine OBSearch (*9*). The method transforms each object into a compact bit string representation that is easier to search. Since the index is compressed and it allows also to solve multiple queries simultaneously, the algorithm runs at a considerable higher speed than competing methods (*8*). When we use similarity search in this text, we "search for the closest" item (range query), or we perform the "nearest neighbor" search (k-NN query).

S1.3 TFBM similarity criterion

The similarity of two TF binding motifs (TFBMs) is calculated by applying the Pearson correlation (*10, 11*) over the position weight matrices (PWM) (*12*) of the motifs. The matrix is transformed into a vector X, giving each element a position in the feature set. Thus, for two vectorized PWMs $X = (x_1, \ldots, x_n)$ and $Y = (y_1, \ldots, y_n)$, the Pearson correlation is defined as:

$$\rho(X, Y) = \frac{\sum_{i=1}^{n}(x_i - \bar{x})(y_i - \bar{y})}{\sqrt{\sum_{i=1}^{n}(x_i - \bar{x})^2}\sqrt{\sum_{i=1}^{n}(y_i - \bar{y})^2}} \tag{1}$$

S1.4 TFBM prediction

Here, we generate a catalog of TFBM predictions. To do so, we extract sequences with intrinsic properties that TFBSs hold (Figure 7D, main text). From these sequences, we generate TFBM predictions (Figure 7E, main text). In the following subsections we explain these steps in detail.

S1.4.1 Compilation of "DNA words" dictionaries

The TFBS motif generation relies on the fragmentation of the genome in sub-sequences or windows of w bases with a potential regulatory meaning. First, we extract a list of DNA sub-sequences of w nucleotide base pairs that hold features intrinsic to TFBS. We call this list B_w. We create lists with sequence lengths in the range $w_{min} \leq w \leq w_{max}$, where $w_{min} = 4$ and $w_{max} = 16$. To generate a list B_w, we employ the techniques already outlined in Figure 7 of the main text. The objective of this step is to extract "DNA words" that are likely to be binding sites of TFs. This step does not focus on the word's location, but only on the fact that the word exists.

S1.4.1.1 Conservation curve method The base pairs shared among multiple aligned species create a pattern useful to filter out spurious sequence noise. We denote such pattern as the conservation curve. To illustrate the procedure of obtaining the conservation curve, we focus on a particular window of $w = 15$ base pairs (Figure 7A, main text). Figure 7C of the main text, shows the entropy curve for the motif MA0142[2] of Jaspar (dark blue line). The entropy curve H is calculated for each cell x_i of each column X_j of a PWM with the following formula:

$$H(X_j) = -\sum_{i=1}^{4} p_j(x_i) \log_4 p_j(x_i), \tag{2}$$

where p_j is the probability of occurrence for the base represented by x_i in the corresponding X_j of the PWM. Additionally, we calculate the conservation curve (light blue in Figure 7C) for the position we are analyzing. For

[2] This is a Oct4 and Sox2 UTF1-like binding

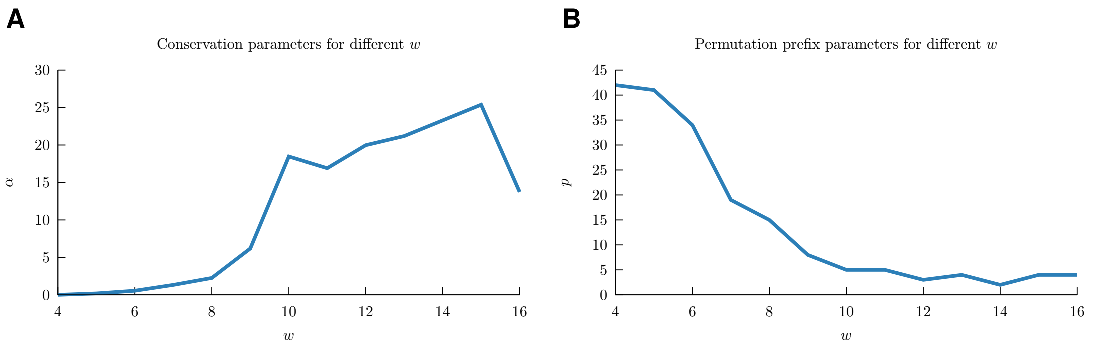

Figure S1: Parameters employed in the generation of the dictionary B_w associated each sequence length w. (A) Distance to the training conservation curves α for each sequence length w. (B) Permutation prefix size p used as a cut off value for each sequence length w.

each position of the sub-sequence, we count how many times the position matches the rest of the species. Then, we normalize this value by dividing by the total number of the species of the alignment. Note the low conservation of the high entropy areas. The entropy is inverse to the conservation level. This relation does not imply that conservation must follow the same shape as the entropy but it gave us the notion that the conservation curve is a useful fingerprint of a TFBS. To generate the list of "DNA words" B_w, we divide the set of TFs into two sets. The validation set V holds only TFs with sites of w base pairs. The training set T contains all the other TFs. For each experimentally validated binding site of each TF in T, we find all the positions in the set of multiple aligned sequences SEQ where the site appears. Then, we calculate the conservation curve for each of them. Since the TFs from SEQ have different lengths w, we normalize these vectors using polynomial splines (*13*) so that they match w. For each sub-sequence of length w in SEQ we compute its conservation curve. We find the closest conservation curve from the list of training conservation curves using a 1-NN query similarity search S1.2. The collection S is a set of w-dimensional vectors. The similarity criteria is the L_2 distance (*10*). For a pair of conservation vectors $X = (x_1, \ldots, x_w)$ and $Y = (y_1, \ldots, y_w)$, it is defined as:

$$L_2(X, Y) = \sqrt{\sum_{i=1}^{w} (x_i - y_i)^2} \quad (3)$$

If their L_2 distance is less than a threshold α, then we consider the site a possible candidate "DNA word". This process creates a list sub-sequences of length w that hold conservation curves similar to those found for experimentally validated TFBSs. The parameter α was tuned to minimize the size of B_w while keeping the sites of each TF in V. Figure S1A displays the α values used for each B_w.

S1.4.1.2 Permutation prefix method The order in which multiple-aligned sequences of different species can be reorganized with respect to a "DNA word" of a specific location is useful to eliminate irrelevant "DNA words". To exploit this ordering we designed the Permutation Prefix Method. A simple way to compare two sequences is to count the number of their different nucleotide bases. This calculation is called the Hamming distance (*10*). For each subsequence in SEQ of length w, the corresponding aligned sequences are ordered by their Hamming distance. Sorting species by closeness in the alignment generates a permutation that is used as a fingerprint to further reduce the size of B_w. We calculate the Hamming distance of the window we want to evaluate against all the corresponding windows in other species. Then, we sort the subsequences by distance. In the case where two distances are equal, a consistent way to order the species is applied for all "DNA words". A permutation of 45 species[3] will

[3]Human is not included in this set

Table S1: Sizes of the unique "DNA words" in each B_w after applying the conservation curve, permutation prefix and merging filters. The size of each B_w is reduced considerably by applying the merge of conservation curve and permutation prefixes.

w	Conservation	Permutation	Merge
4	54	43	36
5	309	156	134
6	1138	709	521
7	1613	2214	1030
8	1122	14051	934
9	7234	127679	5586
10	44863	937187	42348
11	404439	3347589	382116
12	1471333	8420702	1409832
13	1562648	11711210	1207732
14	1972928	15154759	1839176
15	2166061	11894451	1520210
16	164233	8426901	67532
Total	7797975	60037651	6477187

create a unique fingerprint per window in SEQ. This represents a problem because we want to have common permutations in order to filter appropriately the windows. To address this issue, different researchers (*14*, *15*, *16*) have proposed to take only the top p elements of the fingerprint. We hypothesize that evolution occurs at different rates along the genome and therefore a general phylogenetic tree might not consider this. Evolution does not have necessarily to work uniformly at global scale, and local genomic regions may have different conservation levels among species (*17*). Furthermore, the multiple alignment heuristic employed may also introduce artifacts. Our method attempts to identify these artifacts so that if they are common, they will be handled appropriately. The permutation filter tries to compensate these changes by looking locally at interesting targets. We take the training set T and extract the permutation fingerprint but we only keep the top p closest species (see section S1.2). In the example of Figure 7B (main text), the first $p = 8$ species are selected creating the following permutation prefix:

[panTro2, gorGor1, ponAbe2, rheMac2, papHam1, otoGar1, tupBel1, camFam2]

The next step is to find the largest p such that the sites of the validation set V are preserved. Following the conservation curve method of the previous section, we obtain the permutation fingerprint for every sub-sequence of length w in SEQ. We output each "DNA word" only if there is a permutation fingerprint in the training set that matches exactly their first p species. In Figure S1B the p values used for each B_w are displayed. As w grows, the probability of finding equal prefixes diminishes, and therefore p must be reduced.

S1.4.1.3 Merge method The final step of this phase (Figure 7D, main text) is to merge the result of the conservation curve method and the permutation prefix method to include only common elements to both sets of "DNA words". Table S1 shows the resulting sizes for each B_w generated by the conservation curve method, the permutation prefix method and the merging method. Each reduced B_w allowed us to predict novel TFBMs. This reduction is crucial for increasing the quality of the results and for making it computationally feasible to generate the predictions.

S1.4.2 Completion of the TFBM map through clustering

Once we have a set B_w of "DNA words" of potential regulatory meaning, the next step is to extract biologically meaningful motifs. In this step we employ the entropy curve extracted from the motif's PWM (equation 2). For each column of the PWM we calculate its entropy. The outline of the procedure is as follows:

1. Assume that each "DNA word" in B_w is the center of a cluster of "DNA words" of size k. This cluster is generated by obtaining the k closest (measured with a conglomerate distance) elements in B_w to the word we are currently evaluating.
2. Find the subset of elements that create an entropy curve that matches better the entropy curves generated from the training set T.
3. Rank each cluster according to a quality criterion based on a phylogenetic conservation and on the average entropy.
4. Filter predictions that do not follow the general patterns of motifs using the dynamic dimension selection filter.
5. Remove clusters that share similar motifs and group similar clusters.

S1.4.2.1 Cluster computation based on conglomerate distance For each "DNA word" in B_w we obtain the k closest elements in B_w. To find them, we employ the k-NN query of the similarity search techniques described earlier in S1.2. The collection space are the B_w "DNA words" and the distance function employed is described as follows. To find the closest subsequences, a similarity criterion is required. The Hamming distance described in section S1.4.1 could be employed. Nevertheless, we obtained better results with a customized distance function. This function is specially useful for large w. The distance function gives an extra weight to clusters of similar bases. In Table S2 we show six examples of our novel conglomerate distance. The conglomerate distance function is smaller when a larger number of contiguous bases or "chunks" is equal. E.g., the first two cases have a Hamming distance value of 5. On the other hand, conglomerate distance returns a smaller value for the first example. This is because the common elements are next to each other. The same holds true for examples 3 and 4. Since there is a larger contiguous segment in example 4, the conglomerate distance returns a smaller value than in example 3. Figure S2 displays the conglomerate distance algorithm. In line 10, we increment "counter" that holds the number of consecutive equal bases we have seen so far. In this way we can keep track of equal "chunks". In Line 16, we reset the counter and store in "score" the score calculated by the function "cc" (Line 27). We measure in this line how many other bases accompany the current base in the chunk. Finally, in Line 24 we return the difference of the calculated score and the score of a perfect match. Given the conglomerate distance function and a set of sequences B_w, we extract a set of the k closest sequences for each sequence in B_w. We used $k = 20$ in all the experiments.

S1.4.2.2 Sequence cluster fitting heuristic based on entropy curve After obtaining "DNA word" clusters of size k, we must take a subset of these sequences that matches better the entropy curves (eq. 2) of known TFs. We employ the training set T of TFs to remove elements of each cluster that do not contribute to generate an entropy curve that follows the pattern of known TFs. Figures 7C and E (main text) show natural patterns that occur in known TFBMs. The algorithm tries to choose sequences that match already known entropy patterns. Figure S3 shows the cluster fitting heuristic. The heuristic attempts to find the best possible fit for each training TF (line 4). In line 12, we measure the variation of the Euclidean distance of the "fitted" cluster if we incrementally add each sequence in "input". We keep adding sequences as long as the score keeps decreasing. As soon as this is not the case, we stop the loop and store a partial solution (line 20). Finally, in line 22 we return the fitted cluster with the best score.

```
// Conglomerate function definition
// s1 and s2 are of equal size
int conglomerate(String s1, String s2){
  int i = 0;
  // keep track of the size of the current chunk
  int counter = 0;
  int score = 0;
  int windowLength = s1.length();
  while{ (i < windowLength) {
      if (s1.charAt(i) == s2.charAt(i)) {
        counter++;
        if(i + 1 == windowLength){ // last iteration
          // record the last stored counter
          score += cc(counter);
        }
      }else{
        // calculate the previous chunk score
        score += cc(counter);
        counter = 0;
      }
      i++;
    }
  // maximum score - score
  return cc(windowLength) - score;
}
// scoring function
int cc(int size){
  return size * size;
}
```

Figure S2: Pseudo-code of the conglomerate distance function algorithm.

```
// Cluster Fitting
Cluster clusterFit(Cluster input){
 fittedClusters = ∅
 for (Cluster t : T) { // T is formed by clusters of sequences
  double score = Double.MAX_VALUE; // max double value
  // initialize a potential fitted cluster
  Cluster fitted = new Cluster(center(input));
  // iterate through all the sequences or sites in order
  // of closeness to the "center" (the center was added above)
  for(Sequence s : input) {
   // calculate L2 over the entropy curves of the clusters
   newScore = L2(entropyCurve(t), entropyCurve(fitted + s))
   if(newScore < score){
    fitted.add(s);
   }else{
    break; // stop adding members if the score does not improve
   }
  }
  // store the cluster and its score
  fittedClusters.add(score, fitted);
 }
 return the cluster with the lowest score from fittedClusters
}
```

Figure S3: Pseudo-code of the sequence cluster fitting heuristic.

Table S2: Comparison of Hamming and conglomerate distances. The conglomerate distance is smaller when a larger number of contiguous bases is equal in both sequences. Matches based on the Hamming distance are shown in blue.

ID	Sequences	Hamming	Conglomerate
1	”ACTGGGGACT” ”GGTGGGGCAA”	5	$75 = 10^2 - 5^2$
2	”ACTGGGGACT” ”ATTCGAGCCA”	5	$95 = 10^2 - 1^2 - 1^2 - 1^2 - 1^2 - 1^2$
3	”ACTGGGGACT” ”ACTAGGGGCT”	2	$78 = 10^2 - 3^2 - 3^2 - 2^2$
4	”ACTGGGGACT” ”ACTGGGAGCT”	2	$60 = 10^2 - 6^2 - 2^2$
5	”ACTGGGGACT” ”ACTGGGGACA”	1	$19 = 10^2 - 9^2$
6	”ACTGGGGACT” ”ACTGGGGACT”	0	$0 = 10^2 - 10^2$

S1.4.2.3 Cluster prediction ranking Once the clusters are fitted, they are ranked with a quality criteria. In section S1.4.1, we keep for each “DNA word” of length w, the number of times the “DNA word” is conserved c and the total number of appearances of the “DNA word” t. With the recorded c_i and t_i of each site i of the cluster, we calculate the phylogenetic conservation score dividing the total number of conserved instances by the total number of instances, $score = \sum c_i / \sum t_i$. This score is similar to the one implemented by Xie *et al.* (*11*). Additionally, to deal with large w in which sequences appear only once we defined a “cscore” normalizing, the total number of appearances by the maximum number of appearances $max(t)$ registered. We add the phylogenetic conservation score and the cscore to create a ranking value.

$$R = score + cscore = \frac{\sum c_i}{\sum t_i} + \frac{\sum t_i}{max(t)} \tag{4}$$

Higher values of the phylogenetic conservation score represent a better score. Finally, in the case the score is the same, the order is decided by the average entropy:

$$AH(X) = \frac{\sum_{j=1}^{w} H(X_j)}{w} \tag{5}$$

where H is the entropy (Eq. 2) and X is the PWM with w columns.

S1.4.2.4 Sequence cluster filtering based on dynamic dimension selection (DDS) The ranking step of the previous section generates a large number of potential TFBMs. We create a feature set for each potential cluster and then we employ a filter to remove clusters that do not possess certain properties intrinsic to TFs. The feature set consists of the following properties:

1. Phylogenetic score (see previous section and (*11*)).

Table S3: Example of a large magnitude differences dataset.

ID	d_1	d_2	d_3	d_4	d_5	d_6	d_7	d_8	d_9	d_{10}
1	1.1	100	1.2	1.6	1.1	1.2	1.2	1	1	1
2	1.4	1.4	1.4	1.5	100	1.4	1.2	1.2	1	1
3	1	1	1	1	1	1	2	100	2	2
4	20	20	20	20	20	20	20	20	20	20

2. Average number of conserved α values (section S1.4.1)

3. Total number of conserved sites.

4. Average entropy per base (Eq. 5).

5. Entropy curve (normalized to w components).

6. Distance distribution (the histogram of distances between all the sites in the cluster including multiple appearances of the same site (normalized to w components).

This ensemble generates a feature vector of $(2w + 4)$ components per cluster (items 5 and 6 contain w features each, plus the first four items). Since the training set T for sequence length w is formed of the sequences with lengths $w_T \neq w$ (with $w_{min} \leq w_T \leq w_{max}$), we need to rescale the lengths of features 5 and 6 of the training set to the same length w, using a polynomial spline (*13*) approach. Next, we train the dynamic dimension selection (DDS) method. This method filters out clusters that do not follow intrinsic patterns of TFs. One approach is to employ an L_p metric (*10*) to find similar features based on a distance threshold. Another approach is to employ a one class support vector machine. These two approaches did not filter out enough number of clusters, therefore we had to implement a novel filtering technique. The proposed DDS filtering technique is loosely based on the ideas of Tung *et al.* (*18*) and Aggarwal *et al.* (*19*). These authors recognized the need to take into account a subset of the feature vector to compensate for large differences in magnitudes between components (even when the magnitudes have been normalized).

An illustrative example was presented in Tung *et al.* (*18*). Consider the following 10-dimensional query object:

$$Q\text{=(1,1,1,1,1,1,1,1,1,1)}$$

The search of the nearest neighbor in Table S3 based on the Euclidean distance will return object 4. Nevertheless, if we consider the other three objects, each component is closer except for those with a value of 100. Tung *et al.* (*18*) and Aggarwal and Yu (*19*) tackled this problem by only focusing on components with high similarity but at the end their algorithms merge the distances into a single final. The six different types of features (encoded in a $(2w + 4)$ dimensional vector, Table 2 of main text) correspond to properties of different nature, and thus, are not directly comparable. We propose a filter that takes into account the insights of Tung *et al.* (*18*) and Aggarwal and Yu (*19*) while avoiding different dimension mixing. Thus, normalization is not required and the notion of distance function is not applicable anymore.

DDS receives a feature vector, a parameter $dimThreshold$ that indicates the minimum number of components to output a match, a parameter $iterThreshold$ that indicates the maximum number of iterations the algorithm must perform before it reaches the minimum set of components, and a parameter $hamThreshold$ used to perform a final filtering step. Given a d-dimensional object query $Q = (q_1, \ldots, q_d)$, for each dimension q_i DDS orders the objects in the database according to the distance to q_i. In the Table S3 example, for $q_1 = 1$, the object IDs list would be: ((3), (1), (2), (4))[4]. There could be cases where a component value is the same for a number of objects.

[4]The parentheses enclose a list of elements

Table S4: Parameters of the DDS filter for each "DNA word" length w.

w	$iterThreshold$	$dimThreshold$	$hamThreshold$
4	18	4	1
5	347	15	3
6	104	10	1
7	204	17	2
8	215	17	2
9	87	13	1
10	80	17	0
11	95	20	0
12	53	19	0
13	123	24	0
14	108	26	0
15	291	31	0
16	70	20	0

In those cases the object is returned in a list. I.e., in the case of q_7, the returned value would be: ((1, 2), (3), (4)). These sorted results $L = (l_1, \ldots, l_m)$ (where m is the number of object in the dataset) are iterated one list l_j at a time. Figure S4 shows the complete pseudo-code of the DDS algorithm. In line 11 we perform this first matching operation. Next, we record incrementally the object IDs found in each individual component (line 21). As soon as we find a certain object ID that appears at least $dimThreshold$ times, we stop the search (line 25). If this break condition occurs, then $j \leq iterThreshold$ holds (line 30). If we exceed the number of iterations $iterThreshold$ the condition in line 30 returns false. We have added an additional filter in this line. We store the components used to validate the training data. We consider the set of components a binary vector. Those positions used to accept the feature vector are set to 1. Given $dimThreshold$ and $iterThreshold$, we have precomputed a binary vector B for all the objects in the database (when the object in question is removed). The function $hammingFilter$ finds the closest mask in B for each query Q. We accept the result only if the Hamming distance of the closest vector and the query is $\leq hamThreshold$. The process of finding masks also helps to find suitable $iterThreshold$ and $hamThreshold$ parameters. It is only required to choose an appropriate $dimThreshold$. Table S4 shows the parameters used to filter our predictions for different w.

DDS is a powerful filter that reduces the cluster predictions to a fraction. Normalization steps are required for different items if one wants to employ techniques like the one class support vector machines (*20*). Nevertheless, our method treats each single dimension independently and therefore normalization is not required. Instead of dealing with a range of values that mix different component values, we only use $iterThreshold$ as a substitute. Due to this ability of reducing the number of predictions (Table S5), and the fact that B_w was already reduced substantially by the filters designed to compile the "DNA words" (Table S1), the method was able to produce a manageable number of TFBM predictions.

S1.4.2.5 Similar motif removal and cluster merging The final step in the TFBM prediction is the removal of clusters where the 80% of sites overlap. We remove the cluster that has a lower ranking score. Finally, we create groups of clusters that share Pearson similarities above 0.85. With this step the prediction of TFBMs is complete. The output is a list of groups of "DNA words" clusters that are potential TFBMs.

S1.4.3 Predicted motif validation and significance analysis

Given the list of TFs extracted from Jaspar and Transfac databases (Section S1.1), we calculate how many of these motifs are actually predicted by our algorithm. We employ the Pearson correlation (section S1.3) with a threshold

```
// Accepts a feature vector and returns true
// if the vector is a strong match
Boolean dds(double[] feature, int dimThreshold,
                int hamThreshold, int iterThreshold){

 // L is a n+1 tuple (value_i,[id_1,id_2,...id_n]) that holds the
 // value at dimension i and the n training object ids
 // that hold this value.
 // Find the closest tuples L per dimension i of the feature vector
 // ordered closeness to feature[i].
 List<L> ls = match(feat);
 // the object id values that we have found so far
 MultiSet<ID> foundIds;
 int j = 0; // current level
 // Iterate ls until founding an object appearing in at least
 // dimThreshold dimensions.
 while(j <= iterThreshold){
  for(L l : ls){
   // obtain current ids of the list and remove this element
   List<ID> ids = removeFirstAndReturnTuple(l);
   foundIds.addAll(ids); // add current ids.
   //maxIdAppereances counts in how many dimensions each id appears,
   //and returns the largest count.
   if(maxIdAppereances(foundIds) >= dimThreshold){
     break;
   }
  }
  j++;
 }
 return j <= iterThreshold &&
        hammingFilter(maxIdList(foundIds), hamThreshold);
}
// make sure that the matching dimensions are close to the matching
// dimensions of the learned data.
Boolean hammingFilter(List<ID> maxId, hamTheshold){
// Each ID structure holds the dimension it corresponds to.
// We have stored a set of masks holding the dimensions that match
// the training data. The function returns true if the hamming
// distance to the closest of these components is ≤ hamThreshold
}
```

Figure S4: Pseudo-code of the Dynamic Dimension Selection (DDS) filter algorithm.

Table S5: DDS filter reduction effect for each B_w with the number of "DNA words" before and after applying the filter for each word length w.

w	Before	After
4	36	31
5	134	132
6	521	504
7	1030	913
8	934	908
9	5586	312
10	42348	237
11	382116	309
12	1409832	549
13	1207732	354
14	1839176	253
15	1520210	291
16	67532	211
Total	6477187	5004

of 0.85 to accept a prediction as successful. We performed a total of 509 validations against experimental data and 416 had a Pearson correlation greater than 0.85. We generated also a set of random control PWMs to estimate the probability that our motif predictions occur by chance. For Jaspar and Transfac PWMs we shuffled the elements of each column while preserving the column order. This is to account for nucleotide compositional biases (*11*). For each of our predictions, we calculated how many had a similarity threshold of 0.85 or more against this random dataset. Only 1% of our predictions matched this control dataset.

S1.4.4 TFBS prediction

Given each B_w previously generated (section S1.4.1), we search the upstream5000 dataset by only looking at windows belonging to B_w. Since each sequence holds features intrinsic to TFBSs, we only concentrate on this "distilled"version of promoter regions. Based on the Berg-von Hippel method (*21*) we proceed as in Sarkar *et al.* (*22*), the binding energy m of the affinity of a motif with a PWM σ to a sequence $X = (x_1, \ldots, x_w)$ is calculated by:

$$m(X,\sigma) = \frac{\sum_i^w log\left(\frac{P(x_i|\sigma_i)}{P(x_i|\sigma_i')}\right)}{w} \quad (6)$$

where σ' is the background frequency of the four nucleotides across B_w. $P(x_i|\sigma_i)$ denotes the normalized frequency of a nucleotide x_i in the column σ_i of the position weight matrix. By obtaining the binding energy with m for each sequence in B_w, we calculate a binding energy z-score for each motif. To achieve this, we compute the distribution of binding energies of a motif against the entire B_w. We only accept motifs that have an energy z-score of 2.0. Additionally, we stored the average number of matching species per base for each sequence.

S1.5 Combinatorial binding pattern generation

Once the catalog of individual TFBMs is obtained, we find the way how they group with each other forming combinatorial binding patterns (CBPs). The CBP algorithm searches for similar motif sets and then tries to align them taking into account the following parameters:

- $minSize$: Minimum number of motifs to appear in the CBP

```
1  // CBP generation
2  // g: base group used to generate a new CBP
3  // gs: complete list of groups
4  Group generateCBP(Group g, List<Group> gs,
5                         int minSize, int minInstances){
6      // get groups that share at least minSize motifs.
7      Group result = g; // result set
8      for(Group sg : similarGroups(g, gs, minSize)){
9          // find common elements
10         Group result_t = sg ∩ result;
11         // make sure result >= minInstances
12         if(calculateInstances(result_t) < minInstances){
13             break;
14         }
15         result = result_t;
16     }
17     return result;
18 }
19
20 // find similar groups that share at least minSize motifs
21 // order the result by number of hits.
22 List<Group> similarGroups(Group g, List<Group> gs, minSize);
```

Figure S5: Pseudo-code of the CBP generation algorithm.

- $minInstances$: Minimum number of promoters where the CBP applies
- $minThickness$: Minimum number of aligned motifs (motif-level conservation)
- t: Maximum number of base pairs between motifs

We define a "group" as a set of motifs each separated by no more than t base pairs. The first step of the CBP generation algorithm is to create these groups. Figure S5 shows the pseudo-code of the CBP generation algorithm. For each extracted group g, the algorithm starts by searching for groups that share at least $minSize$ motifs (line 8). Once this candidate list is found, it is ordered by the amount of similarity shared with g using the $similarGroups$ function. In this case, similarity is the number of shared motifs and higher similarity is better (we break away from the traditional definition of distance). The algorithm greedily adds candidates while $minInstances$ holds true (line 12). At each step we gather common motifs into the "result" variable (line 10). Next, we perform a multiple alignment using the center star algorithm (*23*). This algorithm finds a center group and then uses it to compute pairwise alignment with the other sequences adding spaces as needed. The pairwise alignment is calculated with the Smith Waterman (*24*) aligner. We set the difference cost to 5, and the open and extended gap penalties to 0. Motifs must align in at least $minThickness$ groups to be considered. After the alignment, we check if the generated CBP still satisfies $minSize$ and $minThickness$. Thus, we generate CBPs with topologically aligned motifs. We employed the following parameters: $t = 1000$, $minSize = 3$, $minInstances = 3$, $minThickness = 3$.

S1.5.1 CBP visualization

We created a CBP visualization computer program that shows an abstract representation of the motif alignment in different promoters. We employed graphviz (*25*) and Web Logo (*26*) to represent our alignments.

Each CBP figure contains a list of metrics that summarize relevant characteristics of the CBP:

- COV (coverage): The average number of base pairs per group that are available for binding.
- GEN (generality): Number of genes where the pattern holds true.
- ROB (robustness): The average number of times a motif is repeated within the pattern.
- SIG (significance): The z-score of the actual GEN in the distribution of GEN that would be generated if the promoters are randomly shuffled (section S1.5.2).
- AL: The multiple alignment score, higher values represent better alignment. The cost matrix holds the same value, 5, in each cell.

In our visualizations, each arrow connecting two motifs a and b means that a motif b is to the "right" of motif a. The thickness of the arrow measures the percentage of promoters where this relationship occurs. The gradient "D" represents the number of base pairs that separate each binding site.

S1.5.2 CBP significance analysis

To measure if our CBPs could occur at random we apply iteratively the following procedure:

1. Shuffle all the motif groups.
2. For each CBP, count how many promoters still hold the motifs of the CBP in the randomized group set. We store this value so that we can generate a z-score during the final stage.

For each CBP we generate a z value based on the number of promoters where the CBP is present in upstream5000 with respect to the appearance in a random promoter set.

S1.5.3 Predictions with CBP significance

We take a subset of the motif predictions generated in section S1.4 based on the CBP significance score previously calculated. We take only those predictions that appear in CBPs with a SIG z value of 3.0 or greater. The set of motif predictions with CBP significance is denoted by STFBM.

S1.6 Computer program implementation

All the algorithms of sections S1.4 and S1.5 were implemented in Java 1.6. The visualizations of section S1.5 were implemented in Clojure 1.2. Gene ontology enrichment statistical significance analysis was developed in Matlab (R2009b).

A number of libraries were employed:

- Graphviz (2.29.20110517.0445) (*27*)
- OBSearch 0.9.9g (*9*)
- Tokyo Cabinet 1.17 (*28*)
- JAligner 1.0 (*29*)
- Colt 1.2 (*30*)
- Apache Commons 1.0 (*31*)
- XStream 1.3.1 (*32*)

- DSIUtils 1.0.7 (*33*)
- Fastutil 5.1.5 (*34*)
- Freehep 2.0.1 (*35*)
- GSon 1.4 (*36*)
- Guava R07 (*37*)
- Berkeley DB 3.3.75 (*38*)
- Apache Commons Math 2.1 (*13*)

For reference only, the approximated execution time of the process in CPU hours was recorded:

- B_w generation: 210 hours.
- Motif prediction: 13 days
- CBP generation: 240 days.
- Ontology calculation: 1 day.

To reduce the long computing times, we used a wide range of machines. E.g., a 120 core, AMD Opteron 2.6 GHz cluster with a total of 160 GB RAM to a 512 core Itanium 2 1.6 GHz cluster with a total of 1.4 TB RAM.

S2 Supporting results

S2.1 TFBM and CBP significant ontologies representation in ontology maps

The designed ontology maps are projections of the high dimensional space of STFBMs and CBPs and their respective statistically significantly enriched ontologies. Figure S6 shows cellular component ontologies for the CBPs. Figures S7, S8 and S9 show the molecular function, biological process and cellular component ontologies respectively, for the set of TFBMs that are embedded in the CBP set (denoted as STFBMs). Each CBP or each STFBMs is linked to its corresponding set of significant molecular function ontology terms. CBPs or STFBMs that share common ontology terms are also linked together. Regions with the same color represent the same cluster of objects. Borders of the "countries" underline boundaries of CBP or STFBMs clusters. The ontology term font size is adjusted to reflect the frequency with which it is associated to the CBPs or STFBMs.

As in the case of the CBPs, for the STFBMs there is a strong transcription-related component in the lower left region of Figure S7. Similarly, in Figure S8, development-related ontologies are strong in the center-left region of the figure.

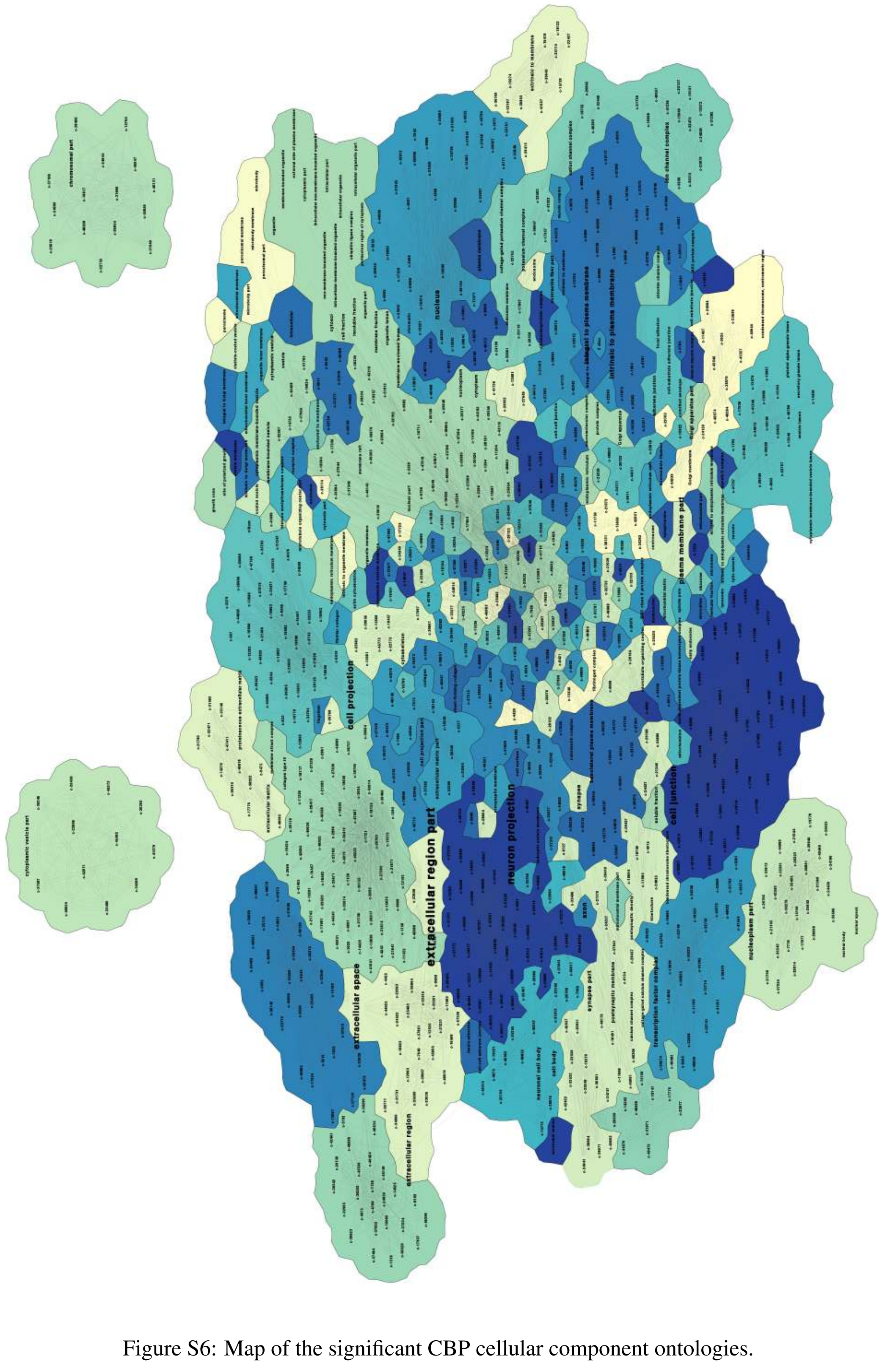

Figure S6: Map of the significant CBP cellular component ontologies.

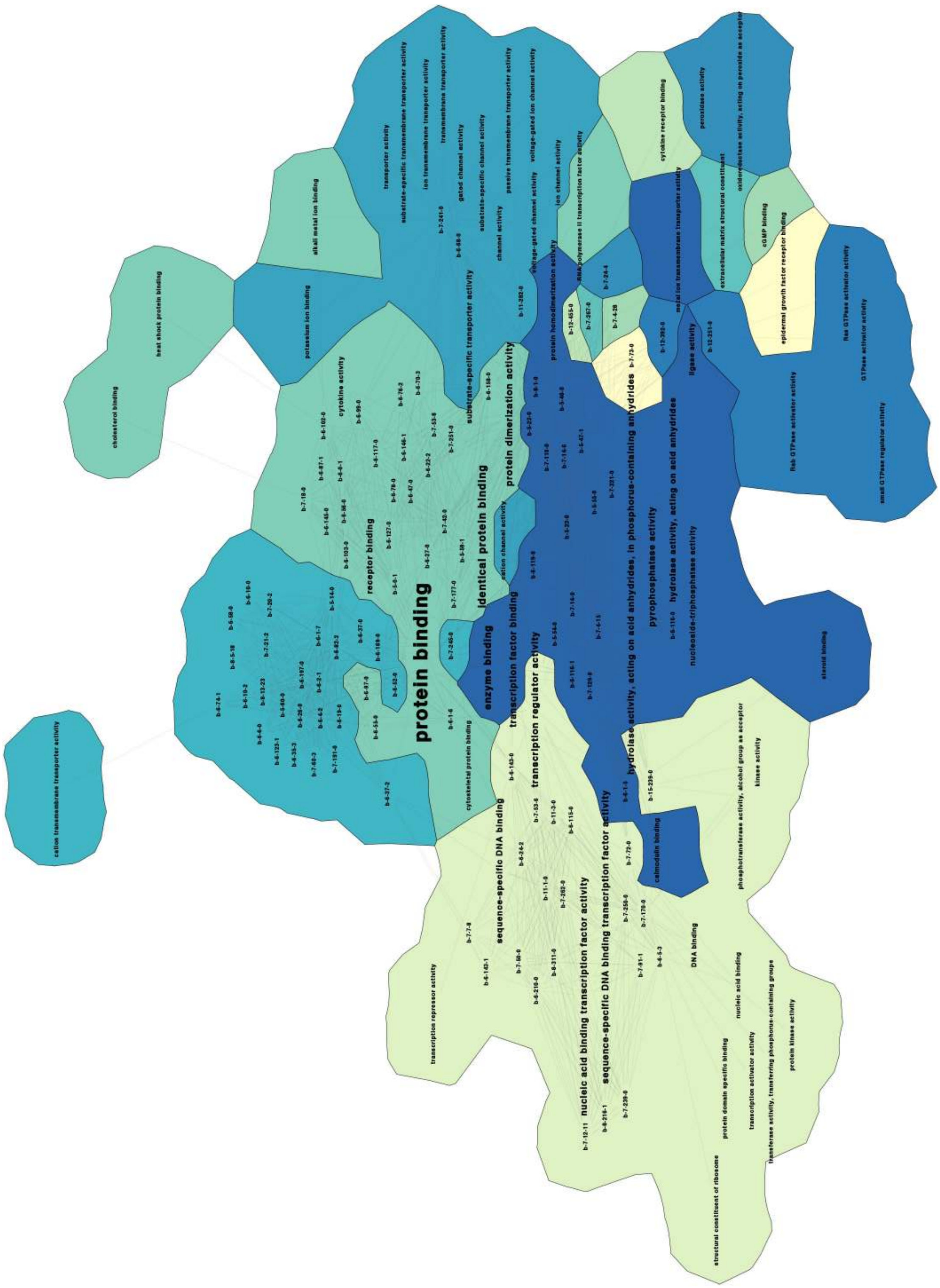

Figure S7: Map of the significant motif (STFBM) molecular function ontologies.

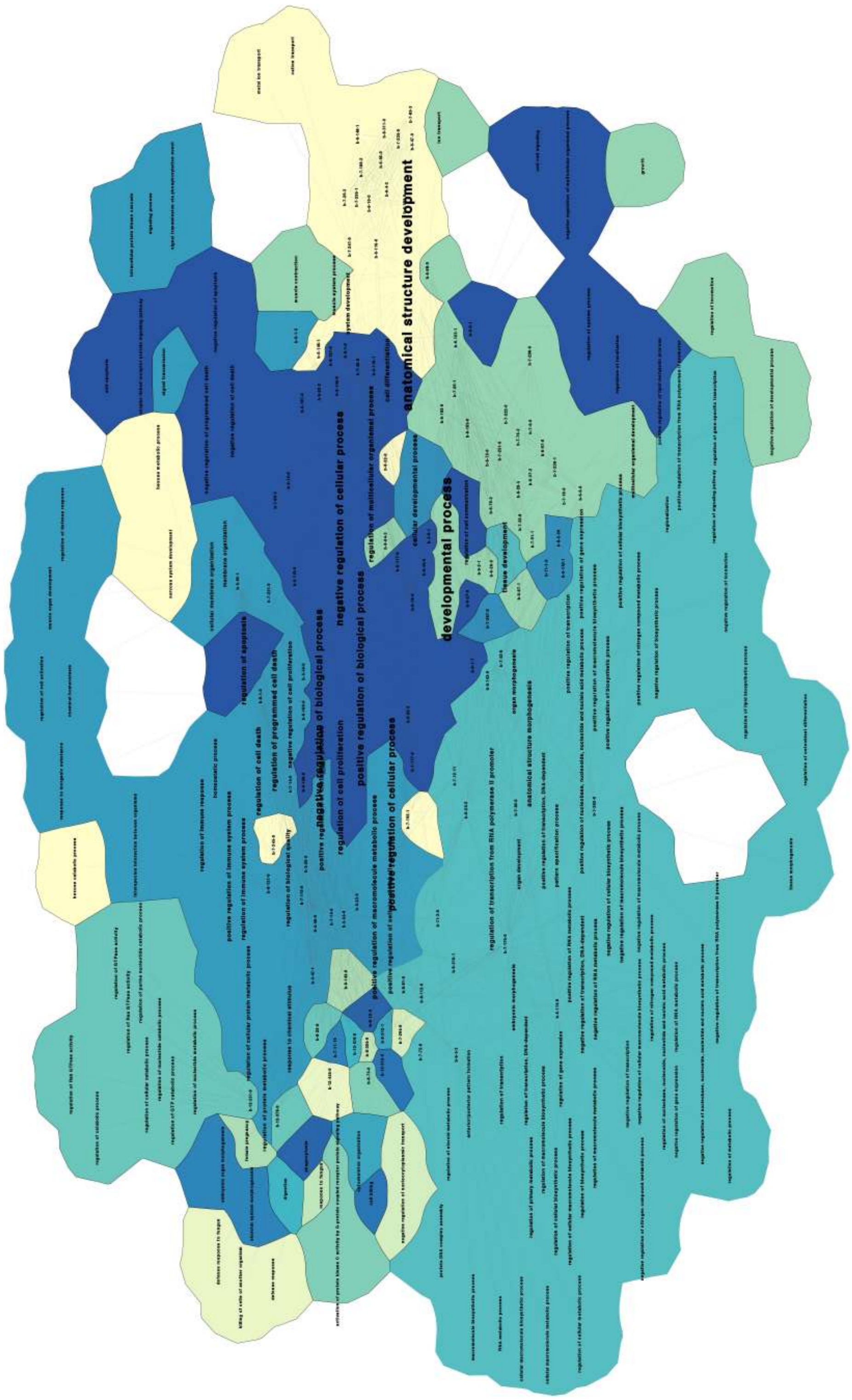

Figure S8: Map of the significant motif (STFBM) biological process ontologies.

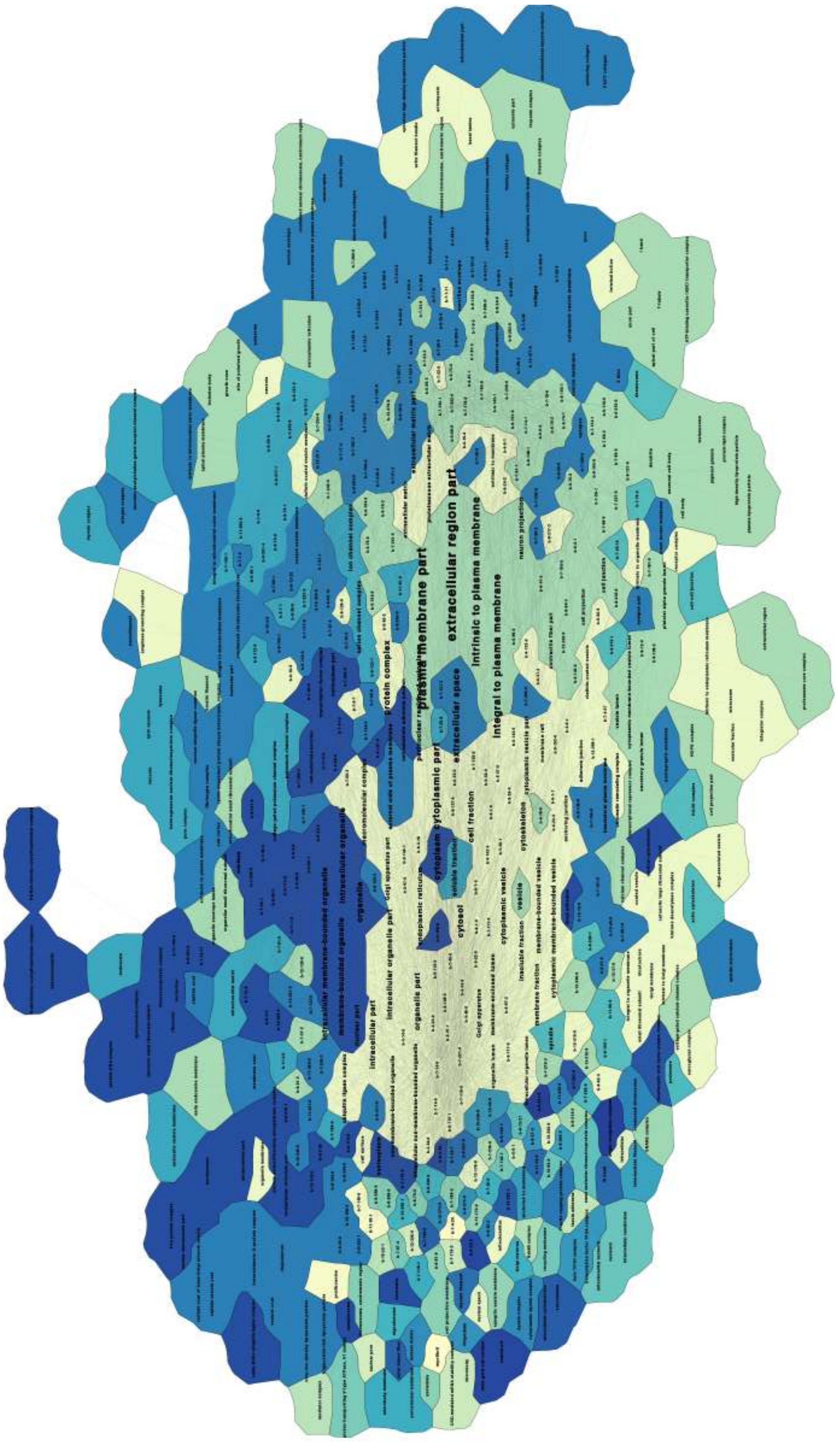

Figure S9: Map of the significant motif (STFBM) cellular component ontologies.

S2.2 TFBMs validation against the Jaspar and Transfac databases

Table S6: Predicted TFBMs and the corresponding validations that matched a known binding motif in the Transfac or Jaspar databases. The values inside parenthesis represent the correlation for the case in which the match is performed with a motif inside the cluster that is not the top element of the cluster. The ID of the motif is shown alongside with the binding motif logo for our prediction and the known motif. We denote as ρ the Pearson correlation between our prediction and the known motif. The Jaspar and Transfac motifs are plotted using the original sites. The Pearson correlation is calculated over the motifs that can be created over the original "DNA word" dictionary. The motifs are sorted by motif length.

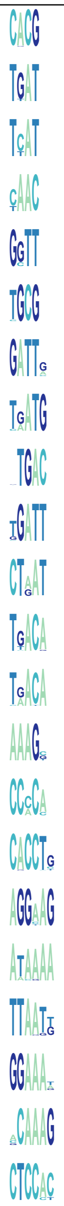

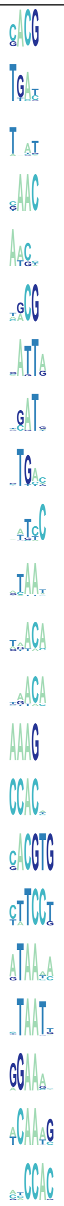

ID	Predicted motif	Known motif	ρ	Known TF
b-4-0-0			0.982	P$STF1_01
b-4-1-0			0.923	V$ATF3_Q6
b-4-2-0			0.867	MA0094.1
b-4-7-0			0.95	P$MYBAS1_01
b-4-8-0			0.852	P$C1_Q2
b-4-20-0			0.869	V$HTF_01
b-5-1-0			0.853	MA0075.1
b-5-2-0			0.856	MA0036.1
b-5-4-0			0.957	V$MAFB_01
b-5-9-0			(0.893)	(P$ANT_01)
b-5-13-0			(0.925)	(V$OCT1_03)
b-5-21-0			0.881	MA0233.1
b-5-26-0			0.935	MA0217.1
b-5-33-0			0.978	MA0064.1
b-5-39-0			(0.908)	(V$KID3_01)
b-6-0-0			(0.998)	(P$EMBP1_02)
b-6-1-0			0.958	V$PEA3_Q6
b-6-3-0			0.921	V$HOXA13_01
b-6-4-0			0.91	MA0132.1
b-6-15-0			0.961	V$NFAT1_Q6
b-6-16-0			0.928	V$LEF1_Q2
b-6-17-0			(0.93)	(MA0130.1)

ID	Predicted motif	Known motif	ρ	Known TF
b-6-24-0			0.97	MA0056.1
b-6-25-0			(0.891)	(V$NKX32_01)
b-6-28-0			(0.888)	(F$NIT2_01)
b-6-29-0			0.878	V$HNF4_Q6_02
b-6-31-0			0.931	MA0231.1
b-6-32-0			0.936	P$PBF_Q2
b-6-35-0			(0.951)	(MA0151.1)
b-6-40-0			0.989	V$AML1_01
b-6-49-0			0.902	MA0261.1
b-6-61-0			0.859	MA0089.1
b-6-63-0			0.914	MA0204.1
b-6-65-0			(0.852)	(MA0212.1)
b-6-66-0			(0.889)	(MA0254.1)
b-6-69-0			0.931	MA0021.1
b-6-83-0			0.89	MA0234.1
b-6-86-0			0.952	MA0260.1
b-6-89-0			0.946	MA0006.1
b-6-98-0			0.939	V$RBPJK_01
b-6-115-0			0.955	V$E2F_Q2
b-6-125-0			0.921	MA0095.1
b-6-139-0			(0.916)	(V$CREB_Q3)
b-6-152-0			(0.872)	(MA0037.1)
b-6-192-0			0.875	P$BZR1_01
b-6-208-0			0.853	MA0185.1
b-7-0-0			(0.923)	(MA0093.1)
b-7-1-0			(0.942)	(P$PEND_01)
b-7-3-0			(0.938)	(MA0256.1)
b-7-4-0			(0.943)	(V$IRF1_Q6)

ID	Predicted motif	Known motif	ρ	Known TF
b-7-5-0	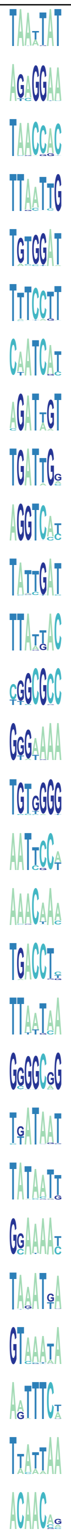	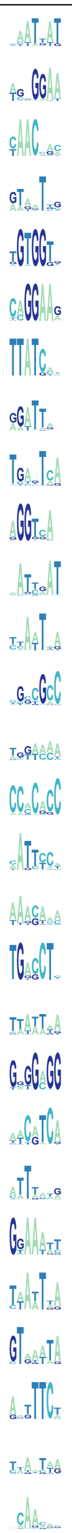	(0.946)	(P$ATHB5_01)
b-7-7-0			0.906	MA0081.1
b-7-8-0			(0.89)	(P$GAMYB_01)
b-7-11-0			(0.864)	(V$EN1_01)
b-7-13-0			(0.955)	(V$AML1_Q4)
b-7-14-0			(0.893)	(MA0026.1)
b-7-16-0			(0.853)	(I$MTTFA_01)
b-7-20-0			(0.894)	(V$GTF2IRD1_01)
b-7-34-0			(0.911)	(MA0099.2)
b-7-35-0			0.938	V$PPARG_Q6
b-7-36-0			(0.912)	(V$CDPCR1_01)
b-7-43-0			(0.867)	(MA0221.1)
b-7-44-0			0.909	P$ERF2_01
b-7-47-0			(0.854)	(MA0152.1)
b-7-53-0			(0.959)	(V$CACD_01)
b-7-59-0			0.877	I$SD_Q6
b-7-60-0			0.934	V$SRY_01
b-7-61-0			0.97	V$PPARA_Q6
b-7-65-0			0.892	MA0448.1
b-7-67-0			0.864	V$ETF_Q6
b-7-71-0			(0.885)	(MA0129.1)
b-7-73-0			(0.857)	(V$CDXA_01)
b-7-87-0			0.855	V$HMGIY_Q6
b-7-95-0			(0.879)	(MA0208.1)
b-7-106-0			0.927	P$GT1_Q6
b-7-107-0			0.952	V$IRF8_Q6
b-7-159-0			0.867	MA0194.1
b-7-161-0			0.883	MA0133.1

ID	Predicted motif	Known motif	ρ	Known TF
b-7-166-0			0.923	V$SOX10_Q6
b-7-197-0			0.936	MA0124.1
b-7-204-0			0.983	I$BRK_Q6
b-7-253-0			0.917	V$DBP_Q6
b-7-271-0			0.866	I$DEAF_01
b-7-283-0			0.873	MA0173.1
b-8-0-0			0.952	V$MAZ_Q6
b-8-1-0			(0.868)	(N$TRA1_01)
b-8-2-0			0.919	V$ETS_Q6
b-8-5-0			(0.944)	(MA0229.1)
b-8-6-0			(0.861)	(MA0125.1)
b-8-12-0			(0.957)	(V$PU1_Q6)
b-8-13-0			0.987	MA0008.1
b-8-21-0			(0.884)	(V$TBP_01)
b-8-22-0			0.869	V$MZF1_01
b-8-30-0			0.953	V$E2A_Q6
b-8-36-0			(0.871)	(MA0222.1)
b-8-37-0			(0.864)	(V$CDPCR3HD_01)
b-8-40-0			0.961	V$P300_01
b-8-41-0			0.891	N$TRA1_02
b-8-52-0			0.885	V$GR_Q6_01
b-8-60-0			0.93	V$OTX_Q1
b-8-62-0			(0.883)	(V$FRA1_Q5)
b-8-63-0			0.987	V$RBPJK_Q4
b-8-69-0			0.948	MA0213.1
b-8-70-0			(0.874)	(MA0237.1)
b-8-72-0			(0.888)	(MA0011.1)
b-8-79-0			(0.923)	(V$AP1_Q4_01)

ID	Predicted motif	Known motif	ρ	Known TF
b-8-90-0	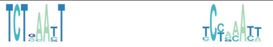		0.876	V$AP3_Q6
b-8-98-0	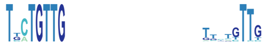		0.922	V$BRCA_01
b-8-99-0	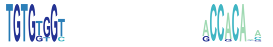		(0.975)	(V$OSF2_Q6)
b-8-102-0	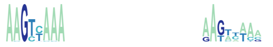		0.878	I$TLL_Q6
b-8-126-0	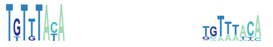		0.901	MA0157.1
b-8-147-0	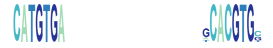		(0.907)	(P$BHLH66_01)
b-8-158-0	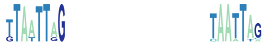		0.927	V$CHX10_01
b-8-159-0	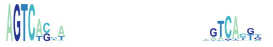		(0.872)	(MA0067.1)
b-8-165-0	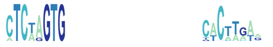		0.86	MA0247.1
b-8-166-0	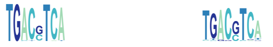		0.995	MA0018.2
b-8-169-0	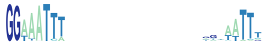		0.868	V$HMGIY_01
b-8-170-0	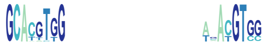		(0.852)	(MA0128.1)
b-8-179-0	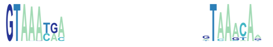		(0.944)	(MA0031.1)
b-8-208-0	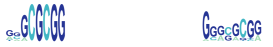		0.87	V$ZF5_01
b-8-216-0	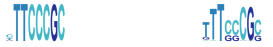		0.907	V$E2F1DP2_01
b-8-221-0	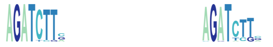		0.99	MA0121.1
b-8-226-0	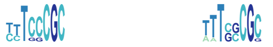		0.884	V$E2F4DP1_01
b-8-231-0	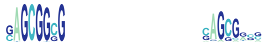		0.882	V$BEN_01
b-8-345-0	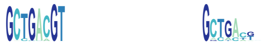		0.852	MA0117.1
b-9-0-0	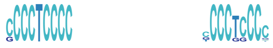		0.982	V$CKROX_Q2
b-9-3-0	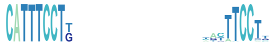		(0.878)	(MA0136.1)
b-9-4-0	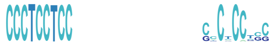		0.895	V$WT1_Q6
b-9-5-0	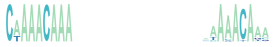		0.921	V$FOXO1_Q5
b-9-7-0	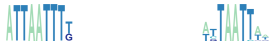		0.87	MA0134.1
b-9-8-0	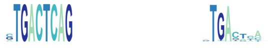		(0.856)	(V$AP1_Q6_01)
b-9-11-0	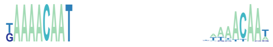		0.912	MA0084.1
b-9-12-0	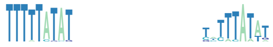		0.911	F$TBP_Q6
b-9-13-0	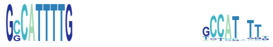		0.879	V$YY1_Q6

ID	Predicted motif	Known motif	ρ	Known TF
b-9-14-0			0.887	V$SPZ1_01
b-9-15-0			(0.894)	(V$ZNF515_01)
b-9-19-0			0.952	V$FOXO1_02
b-9-22-0			0.889	MA0110.1
b-9-23-0			0.891	V$CREBATF_Q6
b-9-25-0			0.871	MA0450.1
b-9-26-0			0.856	V$CIZ_01
b-9-27-0			0.968	MA0242.1
b-9-30-0			0.866	V$STAT_01
b-9-33-0			0.875	MA0460.1
b-9-34-0			0.934	V$NKX3A_01
b-9-37-0			0.965	V$SAP1A_01
b-9-38-0			0.898	V$HNF4_Q6
b-9-43-0			0.906	V$SF1_Q6_01
b-9-47-0			0.92	V$LYF1_01
b-9-48-0			0.935	MA0077.1
b-9-50-0			0.867	V$PBX1_01
b-9-51-0			0.898	V$OCT1_01
b-9-52-0			0.861	V$AREB6_04
b-9-53-0			0.852	MA0078.1
b-9-55-0			0.921	I$CF1_02
b-9-57-0			0.855	MA0126.1
b-9-58-0			(0.894)	(MA0180.1)
b-9-59-0			0.906	P$P_01
b-9-62-0			(0.928)	(V$ZIC3_01)
b-9-69-0			0.894	F$ROX1_Q6
b-9-71-0			0.859	MA0122.1
b-9-85-0			0.854	P$HAHB4_01

ID	Predicted motif	Known motif	ρ	Known TF
b-9-86-0			0.851	MA0253.1
b-9-88-0			0.863	V$GLI3_Q5_01
b-9-94-0			0.9	V$EVI1_06
b-10-1-0			0.902	V$MECP2_02
b-10-2-0			0.881	MA0058.1
b-10-6-0			0.96	V$ELK1_04
b-10-9-0			0.887	V$PBX1_02
b-10-10-0			0.853	V$HLF_01
b-10-12-0			0.859	V$STAT5A_01
b-10-13-0			(0.85)	(V$EBOX_Q6_01)
b-10-14-0			0.897	V$EVI1_05
b-10-16-0			(0.925)	(MA0052.1)
b-10-18-0			0.9	V$KAISO_01
b-10-19-0			0.868	MA0057.1
b-10-20-0			0.862	V$GATA6_01
b-10-22-0			(0.873)	(MA0049.1)
b-10-23-0			0.912	MA0443.1
b-10-24-0			0.857	MA0023.1
b-10-30-0			0.929	V$E2F1_Q6_01
b-10-37-0			0.882	MA0131.1
b-10-38-0			0.864	MA0147.1
b-10-40-0			0.851	P$ZAP1_01
b-10-41-0			0.906	V$NKX25_Q5
b-10-43-0			0.884	F$MATA1_01
b-10-45-0			0.935	V$SP1_Q6_01
b-10-49-0			0.871	MA0154.1
b-10-51-0			0.863	MA0038.1
b-10-52-0			0.885	MA0034.1

ID	Predicted motif	Known motif	ρ	Known TF
b-10-59-0			0.888	V$IK_Q5
b-10-60-0			0.889	V$LHX3_01
b-10-64-0			(0.867)	(MA0123.1)
b-10-66-0			0.893	MA0127.1
b-10-68-0			0.92	MA0016.1
b-10-70-0			(0.931)	(V$SP1_Q2_01)
b-10-71-0			(0.868)	(V$IPF1_01)
b-10-73-0			0.854	MA0015.1
b-10-77-0			0.925	V$POU1F1_Q6
b-10-78-0			0.891	V$MMEF2_Q6
b-10-84-0			0.85	MA0071.1
b-10-85-0			0.896	F$STUAP_01
b-10-87-0			0.918	MA0107.1
b-10-89-0			0.857	F$STE11_02
b-10-91-0			0.866	V$NFKAPPAB50_01
b-10-92-0			0.89	V$FOXO4_01
b-10-94-0			0.869	MA0449.1
b-10-96-0			0.877	MA0459.1
b-10-97-0			0.89	F$CAT8_Q6
b-10-100-0			0.882	F$RGT1_Q5
b-10-106-0			0.853	MA0447.1
b-10-129-0			0.881	P$AGP1_01
b-11-0-0			0.9	I$GAGAFACTOR_Q6
b-11-1-0			0.928	V$SP4_Q5
b-11-2-0			0.877	MA0458.1
b-11-3-0			0.884	V$SP1SP3_Q4
b-11-5-0			0.904	MA0150.1
b-11-7-0			0.892	V$PROP1_01

ID	Predicted motif	Known motif	ρ	Known TF
b-11-8-0			0.88	V$IRF7_01
b-11-9-0			0.909	V$OCT_Q6
b-11-11-0			0.895	P$ID1_01
b-11-14-0			0.866	I$ZESTE_Q2_01
b-11-17-0			0.878	V$SMAD_Q6_01
b-11-19-0			0.856	V$HNF3ALPHA_Q6
b-11-20-0			0.865	MA0445.1
b-11-23-0			0.866	MA0009.1
b-11-24-0			(0.88)	(P$PBF_01)
b-11-26-0			0.866	V$FLI1_Q6
b-11-27-0			0.917	V$ATF5_01
b-11-28-0			0.871	V$LEF1TCF1_Q4
b-11-39-0			0.881	P$ABF_Q2
b-11-40-0			0.863	V$CDX2_Q5_01
b-11-41-0			0.862	V$AHR_Q5
b-11-43-0			0.875	MA0040.1
b-11-45-0			0.881	V$ER_Q6_02
b-11-57-0			0.851	MA0062.2
b-11-64-0			0.858	V$E12_Q6
b-11-71-0			0.962	P$MYB80_01
b-11-82-0			0.87	MA0012.1
b-11-90-0			0.918	V$RORA_Q4
b-11-111-0			0.919	MA0105.1
b-11-123-0			0.861	MA0005.1
b-11-124-0			0.89	MA0025.1
b-11-126-0			0.858	MA0002.1
b-11-147-0			0.879	V$CREB_Q4_01
b-11-149-0			0.896	MA0454.1

ID	Predicted motif	Known motif	ρ	Known TF
b-11-159-0			0.894	P$E2F_Q2
b-11-179-0			0.914	MA0162.1
b-11-190-0			0.892	V$PAX_Q6
b-11-193-0			0.871	V$MRF2_01
b-11-216-0			0.854	V$SOX9_Q4
b-11-248-0			0.932	F$ATF1PCR1_01
b-11-265-0			0.851	V$E2F_Q4_01
b-12-0-0			0.88	V$RORA1_01
b-12-1-0			(0.946)	(V$DR1_Q3)
b-12-25-0			0.851	I$KR_Q6
b-12-62-0			0.882	MA0041.1
b-12-64-0			0.853	MA0048.1
b-12-71-0			0.855	MA0070.1
b-12-79-0			0.856	MA0453.1
b-12-81-0			0.911	V$SATB1_01
b-12-85-0			0.913	V$HNF6_Q6
b-12-115-0			0.859	V$AREB6_02
b-12-116-0			0.854	V$STAT5B_01
b-12-130-0			0.857	V$SRY_02
b-12-152-0			0.85	V$ETS_Q4
b-12-172-0			0.864	MA0083.1
b-12-221-0			0.851	MA0091.1
b-12-265-0			(0.876)	(I$ANTP_Q6_01)
b-12-362-0			0.871	V$AP1_Q2_01
b-12-415-0			0.858	V$CEBP_Q2_01
b-12-462-0			0.853	V$P50RELAP65_Q5_01
b-13-47-0			0.867	MA0135.1
b-13-58-0			0.911	V$HSF2_02

ID	Predicted motif	Known motif	ρ	Known TF
b-13-64-0	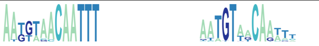		0.875	V$DMRT4_01
b-13-69-0	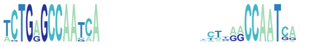		0.857	V$NFY_Q6_01
b-13-86-0	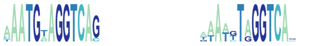		0.867	V$RORA2_01
b-13-123-0	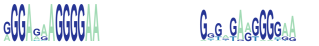		0.879	V$MZF1_02
b-13-136-0	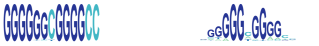		0.906	V$SP1_Q4_01
b-13-137-0	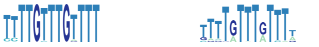		0.952	V$FOX_Q2
b-13-144-0	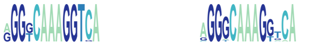		0.957	V$HNF4_01_B
b-13-175-0	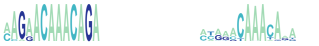		0.851	V$HNF3_Q6
b-13-202-0	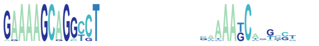		0.851	V$GFI1_Q6
b-13-232-0	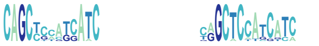		0.888	V$ZID_01
b-13-244-0	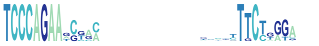		0.863	V$STAT_Q6
b-14-17-0	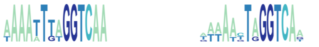		0.853	MA0072.1
b-14-34-0	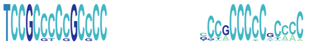		0.884	V$KROX_Q6
b-14-57-0	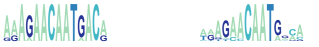		0.91	V$SOX9_B1
b-14-65-0	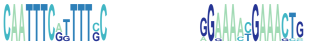		0.857	MA0137.1
b-14-99-0	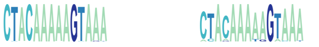		0.996	MA0082.1
b-14-127-0	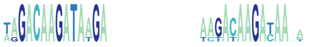		0.882	MA0029.1
b-14-184-0	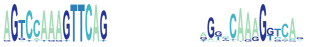		0.855	V$HNF4_Q6_01
b-14-205-0	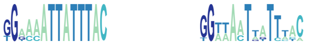		0.88	MA0046.1
b-15-3-0	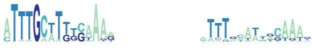		0.866	MA0142.1
b-15-13-0	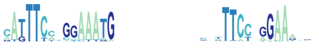		0.867	MA0137.2
b-16-1-0	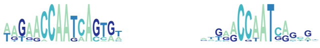		0.867	MA0060.1

S2.3 CBPs validation against Transcompel database

Table S7: CBPs that hold Transcompel rules. The entries from Transcompel (3) are matched against the predicted CBPs. The match is based on the set intersection between the TFBMs of the CBP and the TFs associated with each Transcompel entry. For each CBP rule, GEN is the generality, Sig the significance z-score, Cov the coverage and Rob the robustness. The Transcompel column shows the respective Transcompel rule identifier that matches the CBP.

CBP	Gen	Sig	Cov	Rob	Transcompel
c-23808	13	5.84	174.54	1.69	HLH$C4_001
c-38693	17	4.27	1210.35	4.82	NF1$C4_002
c-19920	17	6.51	894.82	4.81	HLH$C2H2$MADS_001
c-64429	17	2.8	1219.53	5.33	ZIP$C2H2_005
c-64429	17	2.8	1219.53	5.33	ZIP$C2H2_004
c-64429	17	2.8	1219.53	5.33	ZIP$C2H2_003
c-64429	17	2.8	1219.53	5.33	ZIP$C2H2_002
c-25528	14	5.59	72.79	1.17	ZIP$C2H2$ETS_001
c-38693	17	4.27	1210.35	4.82	HLH$C4_002
c-70877	18	2.52	201.28	1.92	ZIP$C4_013
c-38693	17	4.27	1210.35	4.82	HOM$C4_011
c-64429	17	2.8	1219.53	5.33	ZIP$C2H2$REL_001
c-38693	17	4.27	1210.35	4.82	C4$GATA_002
c-38693	17	4.27	1210.35	4.82	C4$C4_009
c-38693	17	4.27	1210.35	4.82	C4$C4_008
c-38693	17	4.27	1210.35	4.82	C4HOMHOM_001
c-38693	17	4.27	1210.35	4.82	C4$C4_007
c-38693	17	4.27	1210.35	4.82	C4$NFY_001
c-38693	17	4.27	1210.35	4.82	HOM$C4_006
c-70877	18	2.52	201.28	1.92	ZIP$C4_010
c-38693	17	4.27	1210.35	4.82	WH$C4_002
c-61477	11	2.93	64.18	1.11	ZIP$C4_009
c-38693	17	4.27	1210.35	4.82	WH$C4_001
c-38693	17	4.27	1210.35	4.82	HOM$C4_002
c-19920	17	6.51	894.82	4.81	HOM$C2H2_002
c-19920	17	6.51	894.82	4.81	HLH$C2H2_002
c-19920	17	6.51	894.82	4.81	HLH$C2H2_001
c-86897	14	1.89	54	1.04	ZIP$NFY_003
c-145680	13	-1.75	776.15	4.45	HOM$HOM_008
c-100532	11	1.38	91.27	1.51	HOM$C2H2_006
c-100532	11	1.38	91.27	1.51	C2H2$NFY_006
c-122391	36	0.46	43.06	1.2	ZIP$REL_044
c-100532	11	1.38	91.27	1.51	C2H2$ETS_011
c-100532	11	1.38	91.27	1.51	C4$C2H2_010
c-100532	11	1.38	91.27	1.51	C4$C2H2_009
c-100532	11	1.38	91.27	1.51	C4$C2H2$C2H2_001
c-100532	11	1.38	91.27	1.51	C4$C2H2_008
c-100532	11	1.38	91.27	1.51	C4$C2H2_007
c-14487	32	7.92	79.53	1.58	C2H2$SMAD_005
c-14487	32	7.92	79.53	1.58	C2H2$SMAD_004
c-1107	11		200.91	1.59	ZIPMYBSTAT_002

CBP	Gen	Sig	Cov	Rob	Transcompel
c-1107	11		200.91	1.59	ZIPMYBSTAT_001
c-100532	11	1.38	91.27	1.51	HLH$C2H2_009
c-86897	14	1.89	54	1.04	ZIP$HOM_005
c-100532	11	1.38	91.27	1.51	REL$C2H2_007
c-93727	9	1.64	40.33	1.07	REL$C2H2_005
c-86897	14	1.89	54	1.04	ZIP$STAT_007
c-145424	59	-1.68	51.31	1.18	ZIP$STAT_006
c-100532	11	1.38	91.27	1.51	C2H2$STAT_003
c-100532	11	1.38	91.27	1.51	C2H2$P53_001
c-86897	14	1.89	54	1.04	ZIP$STAT_004
c-80111	21	2.16	51.67	1.13	ZIP$C4$WH_001
c-100532	11	1.38	91.27	1.51	HOM$C2H2_004
c-106943	41	1.13	39.39	1.2	C2H2$ETS_009
c-106943	41	1.13	39.39	1.2	C2H2$ETS_008
c-106943	41	1.13	39.39	1.2	C2H2$ETS_007
c-106943	41	1.13	39.39	1.2	C2H2ETSETS_001
c-106943	41	1.13	39.39	1.2	C2H2$ETS_006
c-45740	10	3.78	100.9	1.77	C2H2$ETS_005
c-45740	10	3.78	100.9	1.77	C2H2$ETS_004
c-45740	10	3.78	100.9	1.77	C2H2$ETS_003
c-87327	13	1.88	42	1.02	ZIPETSMYB_001
c-14487	32	7.92	79.53	1.58	C2H2$SMAD_003
c-14487	32	7.92	79.53	1.58	C2H2$SMAD_002
c-86897	14	1.89	54	1.04	ZIP$ETS_015
c-24520	12	5.73	67.67	1.21	HLH$ETS_004
c-100532	11	1.38	91.27	1.51	C2H2$NFY_004
c-13072	11	8.4	55.18	1.25	WH$C2H2_002
c-100532	11	1.38	91.27	1.51	C4$C2H2_004
c-100532	11	1.38	91.27	1.51	C4$C2H2_003
c-100532	11	1.38	91.27	1.51	HLH$C2H2_008
c-100532	11	1.38	91.27	1.51	HLH$C2H2_007
c-86897	14	1.89	54	1.04	ZIP$REL_032
c-86897	14	1.89	54	1.04	ZIP$REL_031
c-86897	14	1.89	54	1.04	ZIP$ETS_014
c-100532	11	1.38	91.27	1.51	C2H2$NFY_002
c-100532	11	1.38	91.27	1.51	C2H2$STAT_001
c-86897	14	1.89	54	1.04	ZIP$ETS_013
c-65793	17	2.74	63.18	1.13	ZIP$MYB_002
c-86897	14	1.89	54	1.04	ZIP$RUNT_002
c-86897	14	1.89	54	1.04	ZIP$REL_021
c-86897	14	1.89	54	1.04	ZIP$RUNT_001
c-40867	17	4.11	52.71	1.33	ZIP$ETS_012
c-106943	41	1.13	39.39	1.2	C2H2$ETS_002
c-13072	11	8.4	55.18	1.25	WH$C2H2_001
c-86897	14	1.89	54	1.04	ZIP$HLH_001
c-40867	17	4.11	52.71	1.33	ZIP$ETS_003
c-100532	11	1.38	91.27	1.51	ZIP$C2H2_001
c-86897	14	1.89	54	1.04	ZIP$NFY_002
c-100532	11	1.38	91.27	1.51	REL$C2H2_002

CBP	Gen	Sig	Cov	Rob	Transcompel
c-35991	58	4.49	29.72	1.13	HLH$ETS_002
c-35991	58	4.49	29.72	1.13	HLH$ETS_001
c-106943	41	1.13	39.39	1.2	C2H2$ETS_001
c-100532	11	1.38	91.27	1.51	C2H2$NFY_001
c-76697	24	2.29	49.17	1.22	HLH$C4_003
c-11960	5	8.89	27.2	1	ETS$MADS_007
c-51528	7	3.44	26	1	MADS$HMG_001
c-11960	5	8.89	27.2	1	ETS$MADS_006
c-11960	5	8.89	27.2	1	ETS$MADS_005
c-11960	5	8.89	27.2	1	ETS$MADS_004
c-9902	4	10	24.25	1	HLH$WH_003
c-87796	67	1.86	701.7	6.89	HOM$STAT_002
c-52985	6	3.36	23.67	1	ZIP$HMG_005
c-52985	6	3.36	23.67	1	ZIP$HMG_004
c-29300	45	5.13	30.13	1.11	HLH$ETS_008
c-29300	45	5.13	30.13	1.11	HLH$ETS_007
c-122772	21	0.44	45.81	1.06	ZIP$REL_043
c-29300	45	5.13	30.13	1.11	C4$ETS_003
c-82116	28	2.08	38.43	1.1	ZIP$C4_014
c-29300	45	5.13	30.13	1.11	ETS$SMAD_001
c-95537	25	1.57	35.48	1.01	ZIP$SMAD_005
c-52575	12	3.38	34.42	1.06	ZIP$ETS_025
c-91114	11	1.73	33.18	1.07	ETS$MYB_010
c-103625	6	1.26	27.83	1	ZIPHLHGATA_01
c-35991	58	4.49	29.72	1.13	HLH$HLH_007
c-35991	58	4.49	29.72	1.13	HLH$HLH_006
c-35991	58	4.49	29.72	1.13	HLH$HLH_005
c-29300	45	5.13	30.13	1.11	ZIP$ETS_024
c-44182	4	3.88	33.5	1	HLH$SMAD_003
c-81311	12	2.11	811.92	5.36	HOM$C4_010
c-87796	67	1.86	701.7	6.89	ZIP$HOM_006
c-81311	12	2.11	811.92	5.36	HOM$C4_007
c-29300	45	5.13	30.13	1.11	C4$ETS_002
c-132888	25	-0.16	50.88	1.16	ZIP$REL_042
c-137969	168	-0.57	29.54	1.18	ZIP$REL_041
c-132888	25	-0.16	50.88	1.16	ZIP$REL_039
c-52985	6	3.36	23.67	1	REL$HMG_009
c-52985	6	3.36	23.67	1	REL$HMG_008
c-92988	8	1.66	33	1	REL$MYB_007
c-92988	8	1.66	33	1	REL$MYB_006
c-92988	8	1.66	33	1	REL$MYB_005
c-92988	8	1.66	33	1	REL$MYB_004
c-118950	11	0.62	33.45	1.03	REL$MYB_003
c-92988	8	1.66	33	1	RELMYBMYB_001
c-92988	8	1.66	33	1	RELRELMYB_001
c-122772	21	0.44	45.81	1.06	ZIP$REL_038
c-122772	21	0.44	45.81	1.06	ZIP$REL_037
c-52985	6	3.36	23.67	1	REL$HMG_007
c-122772	21	0.44	45.81	1.06	ZIPRELUNCL_001

CBP	Gen	Sig	Cov	Rob	Transcompel
c-122772	21	0.44	45.81	1.06	ZIPZIPREL_001
c-122772	21	0.44	45.81	1.06	ZIP$REL_036
c-122772	21	0.44	45.81	1.06	ZIP$REL_035
c-132888	25	-0.16	50.88	1.16	ZIP$REL_034
c-25771	74	5.55	30.05	1.15	ETS$STAT$STAT_001
c-76129	4	2.31	29	1	REL$P53_001
c-11960	5	8.89	27.2	1	ETS$STAT$SRF_001
c-122455	9	0.46	26	1.04	ZIP$STAT_003
c-122455	9	0.46	26	1.04	ZIP$STAT_002
c-42414	5	4	28.2	1	HLH$WH_002
c-66091	5	2.72	27.6	1	WH$HOM_002
c-29300	45	5.13	30.13	1.11	ETS$ETS_003
c-11960	5	8.89	27.2	1	ZIPETSMADS_001
c-71498	4	2.5	23	1	ETS$HMG_002
c-35361	4	4.54	18	1	ZIPETSHMG_001
c-29300	45	5.13	30.13	1.11	ZIP$ETS_021
c-29300	45	5.13	30.13	1.11	ETS$ETS_002
c-29300	45	5.13	30.13	1.11	ZIP$ETS_020
c-29300	45	5.13	30.13	1.11	ZIP$ETS_019
c-29300	45	5.13	30.13	1.11	ZIPETSREL_001
c-35991	58	4.49	29.72	1.13	HLH$ETS_006
c-91114	11	1.73	33.18	1.07	ETS$MYB_009
c-64852	8	2.78	32	1	ETS$REL_004
c-64852	8	2.78	32	1	ETS$REL_003
c-35991	58	4.49	29.72	1.13	HLH$ETS_005
c-29300	45	5.13	30.13	1.11	ETS$ETS_001
c-35361	4	4.54	18	1	ZIP$ETS_018
c-18999	6	6.71	30.67	1.28	ETS$MYB_006
c-35991	58	4.49	29.72	1.13	HLH$MYB_001
c-46243	13	3.75	53.31	1.23	ZIPETSMYB_002
c-49609	123	3.55	28.33	1.2	HLH$SMAD_002
c-95537	25	1.57	35.48	1.01	ZIP$SMAD_004
c-29300	45	5.13	30.13	1.11	ETS$MYB_005
c-29300	45	5.13	30.13	1.11	HOM$ETS_003
c-29300	45	5.13	30.13	1.11	ZIP$ETS_017
c-29300	45	5.13	30.13	1.11	ETS$REL_002
c-25771	74	5.55	30.05	1.15	ETS$REL_001
c-29300	45	5.13	30.13	1.11	ETS$RUNT_007
c-87417	5	1.87	25.8	1	MYB$RUNT_004
c-87417	5	1.87	25.8	1	MYB$RUNT_003
c-1843	13	119.02	73.38	1.25	ZIP$C4_012
c-69707	13	2.57	27.46	1	C4$GATA_001
c-114682	30	0.81	32.87	1.08	C4$HMG_001
c-83332	5	2.03	34.4	1	HLH$NFY_005
c-118499	44	0.65	36.27	1.13	ZIP$HLH_002
c-83332	5	2.03	34.4	1	HLH$NFY_003
c-83332	5	2.03	34.4	1	HLH$NFY_002
c-54415	29	3.28	36.79	1.07	HLH$NFY_001
c-49609	123	3.55	28.33	1.2	HLH$SMAD_001

CBP	Gen	Sig	Cov	Rob	Transcompel
c-91426	37	1.72	22.81	1.05	ZIP$SMAD_002
c-95537	25	1.57	35.48	1.01	ZIP$SMAD_001
c-132816	8	-0.15	33.5	1.08	SMAD$HMG_001
c-9902	4	10	24.25	1	WH$WH_003
c-111895	71	0.93	31.08	1.14	ZIP$GATA_003
c-29300	45	5.13	30.13	1.11	ETS$RUNT_005
c-29300	45	5.13	30.13	1.11	ETS$RUNT_004
c-76697	24	2.29	49.17	1.22	HLH$STAT_001
c-87417	5	1.87	25.8	1	MYB$RUNT_002
c-34783	30	4.59	42.67	1.09	ZIP$ZIP_007
c-29300	45	5.13	30.13	1.11	ETS$RUNT_003
c-29300	45	5.13	30.13	1.11	ETS$RUNT_002
c-42826	14	3.97	26.43	1	ZIP$MYB_001
c-35991	58	4.49	29.72	1.13	HLH$HLH_004
c-29300	45	5.13	30.13	1.11	HLH$ETS_003
c-87796	67	1.86	701.7	6.89	HOM$REL_001
c-132888	25	-0.16	50.88	1.16	ZIP$REL_028
c-137969	168	-0.57	29.54	1.18	ZIP$REL_027
c-31586	47	4.89	34.62	1.14	ZIP$REL_026
c-137969	168	-0.57	29.54	1.18	ZIP$REL_024
c-137969	168	-0.57	29.54	1.18	ZIP$REL_023
c-71771	22	2.49	109.41	1.82	ZIP$HOM_004
c-137969	168	-0.57	29.54	1.18	ZIP$REL_022
c-122772	21	0.44	45.81	1.06	ZIP$REL_020
c-122772	21	0.44	45.81	1.06	ZIP$REL_019
c-122772	21	0.44	45.81	1.06	ZIP$REL_018
c-137969	168	-0.57	29.54	1.18	ZIP$REL_017
c-137969	168	-0.57	29.54	1.18	ZIP$REL_016
c-137969	168	-0.57	29.54	1.18	ZIP$REL_015
c-52985	6	3.36	23.67	1	HOM$HMG_001
c-137969	168	-0.57	29.54	1.18	ZIP$REL_013
c-49099	12	3.58	35.92	1.15	ZIP$REL_012
c-49099	12	3.58	35.92	1.15	ZIP$REL_011
c-49099	12	3.58	35.92	1.15	ZIP$REL_010
c-52985	6	3.36	23.67	1	REL$HMG_005
c-29300	45	5.13	30.13	1.11	HOM$ETS_002
c-29300	45	5.13	30.13	1.11	ETS$MYB_001
c-29300	45	5.13	30.13	1.11	ZIP$ETS_011
c-25771	74	5.55	30.05	1.15	ZIP$ETS_010
c-29300	45	5.13	30.13	1.11	HOM$ETS_001
c-42414	5	4	28.2	1	WH$C4_003
c-11960	5	8.89	27.2	1	ETS$MADS_003
c-11960	5	8.89	27.2	1	ETS$MADS_002
c-9902	4	10	24.25	1	WH$WH_002
c-35991	58	4.49	29.72	1.13	HLH$MADS_001
c-9902	4	10	24.25	1	HLH$WH_001
c-25771	74	5.55	30.05	1.15	ETS$HMG_001
c-34783	30	4.59	42.67	1.09	ZIP$ZIP_005
c-34783	30	4.59	42.67	1.09	ZIP$ZIP_001

CBP	Gen	Sig	Cov	Rob	Transcompel
c-137969	168	-0.57	29.54	1.18	ZIP$REL_009
c-137969	168	-0.57	29.54	1.18	ZIP$REL_008
c-64095	28	2.81	44.5	1.13	ZIP$WH_001
c-122772	21	0.44	45.81	1.06	ZIP$REL_005
c-77041	6	2.28	40.5	1.14	ZIP$REL_004
c-122772	21	0.44	45.81	1.06	ZIP$REL_002
c-52985	6	3.36	23.67	1	ZIP$HMG_003
c-52985	6	3.36	23.67	1	ZIP$HMG_002
c-52985	6	3.36	23.67	1	ZIP$HMG_001
c-40270	32	4.15	29.12	1.12	ZIP$GATA_001
c-29300	45	5.13	30.13	1.11	ZIP$ETS_009
c-29300	45	5.13	30.13	1.11	ZIP$ETS_008
c-29300	45	5.13	30.13	1.11	ZIP$ETS_007
c-29300	45	5.13	30.13	1.11	ZIP$ETS_006
c-29300	45	5.13	30.13	1.11	ZIP$ETS_005
c-29300	45	5.13	30.13	1.11	ZIP$ETS_004
c-29300	45	5.13	30.13	1.11	ZIP$ETS_002
c-29300	45	5.13	30.13	1.11	ZIP$ETS_001
c-37782	254	4.35	29.76	1.2	ZIP$C4_008
c-40809	61	4.11	48.54	1.35	ZIP$C4_004
c-42414	5	4	28.2	1	WH$WH_001
c-42414	5	4	28.2	1	WH$HOM_001
c-42414	5	4	28.2	1	WH$NF1_001
c-58234	4	3.08	24.25	1	REL$MYB_002
c-58234	4	3.08	24.25	1	REL$MYB_001
c-41334	5	4.08	18.4	1	REL$HMG_004
c-41334	5	4.08	18.4	1	REL$HMG_003
c-41334	5	4.08	18.4	1	REL$HMG_002
c-41334	5	4.08	18.4	1	REL$HMG_001
c-87796	67	1.86	701.7	6.89	HOM$HOM_005
c-87796	67	1.86	701.7	6.89	HOM$HOM_004
c-87796	67	1.86	701.7	6.89	HOM$HOM_003
c-131974	10	-0.09	113.9	2.66	HOM$C4_004
c-81854	12	2.09	41.17	1.06	HLH$WH_004
c-35991	58	4.49	29.72	1.13	HLH$HOM_001
c-35991	58	4.49	29.72	1.13	HLH$HLH_002
c-35991	58	4.49	29.72	1.13	HLH$HLH_001
c-91114	11	1.73	33.18	1.07	ETS$MYB_004
c-11960	5	8.89	27.2	1	ETS$MADS_001
c-29300	45	5.13	30.13	1.11	C4$ETS_001
c-29300	45	5.13	30.13	1.11	ETS$RUNT_001
c-87417	5	1.87	25.8	1	MYB$RUNT_001
c-106366	4	1.15	21	1	ZIP$HOM_007
c-133358	4	-0.19	26	1	TEA$MADS_001
c-83956	4	2	18	1	GATA$MADS_002
c-83956	4	2	18	1	GATA$MADS_001
c-85158	4	1.96	19	1	STAT$STAT_005
c-85158	4	1.96	19	1	REL$STAT$STAT_01
c-128319	4	0.14	17	1	REL$MYB_009

CBP	Gen	Sig	Cov	Rob	Transcompel
c-85158	4	1.96	19	1	STAT$STAT_004
c-122373	4	0.46	14	1	ZIP$RUNT_004
c-85158	4	1.96	19	1	ZIP$STAT_008
c-88456	4	1.84	15	1	ETS$ETS_004
c-27040	4	5.39	19	1	MYB$STAT_002
c-128319	4	0.14	17	1	REL$MYB_008
c-124261	4	0.36	23	1	REL$C2H2_009
c-112487	4	0.91	22	1	ZIPWHNFY_02
c-112487	4	0.91	22	1	ZIPWHNFY_01
c-112487	4	0.91	22	1	ZIP$HLH_003
c-118824	4	0.63	21	1	WH$NFY_003
c-134723	4	-0.29	21	1	HLH$UNCL_002
c-85158	4	1.96	19	1	STAT$STAT_003
c-16199	4	7.39	18.75	1	HOM$C2H2_005
c-16199	4	7.39	18.75	1	HOM$C4_009
c-94761	4	1.6	18	1	HOM$C4_008
c-83956	4	2	18	1	GATA$HOM_001
c-124261	4	0.36	23	1	REL$C2H2_008
c-107023	4	1.13	27	1	REL$REL_007
c-85158	4	1.96	19	1	REL$STAT_006
c-107023	4	1.13	27	1	ZIP$REL_040
c-107023	4	1.13	27	1	REL$REL_005
c-107023	4	1.13	27	1	REL$REL_004
c-107023	4	1.13	27	1	REL$REL_003
c-107023	4	1.13	27	1	REL$UNCL_001
c-107023	4	1.13	27	1	REL$C2H2_006
c-107023	4	1.13	27	1	ETS$REL_005
c-85158	4	1.96	19	1	STAT$STAT_002
c-85158	4	1.96	19	1	STAT$STAT_001
c-85158	4	1.96	19	1	RELRELSTAT_003
c-85158	4	1.96	19	1	HOM$STAT_001
c-107023	4	1.13	27	1	ZIP$REL_033
c-85158	4	1.96	19	1	RELRELSTAT_001
c-27040	4	5.39	19	1	MYB$STAT_001
c-84553	4	1.98	17	1	C4$C4$STAT_001
c-85158	4	1.96	19	1	ZIP$STAT_005
c-83956	4	2	18	1	GATA$GATA$HOM_001
c-66077	4	2.73	22	1	HOMHOMHOM_001
c-122373	4	0.46	14	1	ZIP$ETS_022
c-88456	4	1.84	15	1	ETS$MYB_008
c-88456	4	1.84	15	1	ETS$MYB_007
c-121413	4	0.51	16	1	ZIP$SMAD_003
c-122373	4	0.46	14	1	ZIP$ETS_016
c-122373	4	0.46	14	1	ZIP$RUNT_003
c-5200	4	15.72	34	1	HOM$RUNT_001
c-88456	4	1.84	15	1	ETS$RUNT_006
c-102079	4	1.32	17	1	C4$C2H2_005
c-102079	4	1.32	17	1	ZIP$C4_011
c-102079	4	1.32	17	1	C4$C4_006

CBP	Gen	Sig	Cov	Rob	Transcompel
c-124261	4	0.36	23	1	HOM$C2H2_003
c-102079	4	1.32	17	1	HOM$C4_005
c-102079	4	1.32	17	1	C4$C2H2_002
c-102079	4	1.32	17	1	C4$C2H2_001
c-118824	4	0.63	21	1	HLH$NFY_004
c-121413	4	0.51	16	1	C2H2$SMAD_001
c-121413	4	0.51	16	1	WH$SMAD_002
c-121413	4	0.51	16	1	WH$SMAD_001
c-121413	4	0.51	16	1	SMAD$SMAD_001
c-121413	4	0.51	16	1	RUNT$SMAD_001
c-85158	4	1.96	19	1	REL$STAT_002
c-118824	4	0.63	21	1	WH$NFY_001
c-85158	4	1.96	19	1	ZIP$STAT_001
c-85158	4	1.96	19	1	REL$STAT_001
c-26381	4	5.47	28	1	ZIP$REL_029
c-122373	4	0.46	14	1	ZIP$REL_025
c-107023	4	1.13	27	1	ZIP$REL_030
c-107023	4	1.13	27	1	ZIP$REL_014
c-83956	4	2	18	1	ZIP$GATA_002
c-16199	4	7.39	18.75	1	ZIP$HOM_003
c-84553	4	1.98	17	1	C4$C4_005
c-122373	4	0.46	14	1	ZIP$ZIP_003
c-106366	4	1.15	21	1	ZIP$ZIP_002
c-106366	4	1.15	21	1	ZIP$UNCL_005
c-122373	4	0.46	14	1	ZIP$UNCL_002
c-106366	4	1.15	21	1	ZIP$UNCL_001
c-127276	4	0.2	18	1	ZIP$REL_007
c-127276	4	0.2	18	1	ZIP$REL_006
c-127276	4	0.2	18	1	ZIP$REL_003
c-127276	4	0.2	18	1	ZIP$REL_001
c-112487	4	0.91	22	1	ZIPZIPHOM_001
c-106366	4	1.15	21	1	ZIP$HOM_001
c-122373	4	0.46	14	1	ZIP$NFY_001
c-127276	4	0.2	18	1	REL$REL_001
c-107813	21	1.1	72.9	2.13	HOM$UNCL_001
c-16199	4	7.39	18.75	1	HOM$HOM_002
c-84553	4	1.98	17	1	HOM$C4_003
c-83956	4	2	18	1	GATA$UNCL_001
c-88456	4	1.84	15	1	ETS$MYB_003
c-84553	4	1.98	17	1	NF1$C4_001
c-21956	4	6.13	19	1	HOM$UNCL_002
c-21956	4	6.13	19	1	HOM$HOM_001
c-21956	4	6.13	19	1	WH$NFY_002

S2.4 *Ab initio* predicted TFBMs that appear frequently inside CBPs (STFBM)

Table S8: *Ab initio* predicted motifs that appear frequently in CBPs (STFBM). The position within this subset is included alongside the prediction ID, the motif logo, the significance z score, the corresponding CBP ID. Additionally, the most significant molecular function, biological process and cellular component ontologies are included for each motif.

ID	Predicted	z	CBP	Function	Process	Component
b-12-72-0	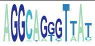	Inf.	c-1539	GO:0004871	GO:0032501	GO:0005886
b-9-74-0	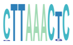	Inf.	c-5	-	-	-
b-7-4-27	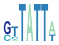	Inf.	c-1546	-	GO:0007155	GO:0016021
b-8-72-1	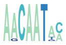	Inf.	c-3	-	-	-
b-7-70-2	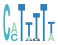	Inf.	c-3	-	-	-
b-7-50-0	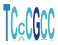	Inf.	c-4	-	-	-
b-7-4-15	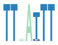	Inf.	c-0	-	-	-
b-8-209-0	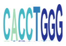	Inf.	c-0	-	-	-
b-11-80-0	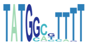	Inf.	c-1551	-	GO:0007275	-
b-7-91-1	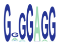	Inf.	c-0	-	-	-
b-7-1-11	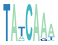	Inf.	c-0	-	-	-
b-8-307-0	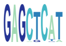	Inf.	c-1526	GO:0003676	GO:0034641	-
b-7-7-4	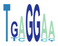	Inf.	c-0	-	-	-
b-12-92-0	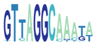	Inf.	c-1543	-	-	-
b-12-516-0	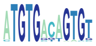	Inf.	c-0	-	-	-
b-8-328-0	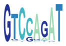	Inf.	c-1542	-	GO:0010646	-
b-9-118-0	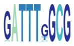	Inf.	c-3	-	-	-
b-12-206-1	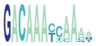	Inf.	c-0	-	-	-
b-8-91-0	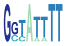	Inf.	c-59	-	-	-
b-8-138-2	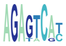	Inf.	c-3	-	-	-
b-7-118-1	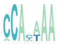	Inf.	c-59	-	-	-
b-8-139-1	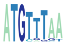	Inf.	c-1553	GO:0004871	GO:0051239	GO:0044459
b-12-426-0	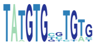	Inf.	c-0	-	-	-
b-8-90-0	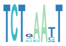	Inf.	c-3	-	-	-

ID	Predicted	z	CBP	Function	Process	Component
b-12-503-0	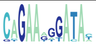	Inf.	c-1551	-	GO:0007275	-
b-6-119-0	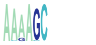	Inf.	c-0	-	-	-
b-7-117-1	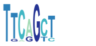	Inf.	c-0	-	-	-
b-8-5-2	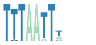	Inf.	c-0	-	-	-
b-12-326-0	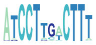	Inf.	c-0	-	-	-
b-7-117-0	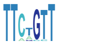	Inf.	c-112	-	-	-
b-8-149-0	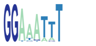	Inf.	c-3	-	-	-
b-8-127-0	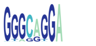	Inf.	c-3	-	-	-
b-6-5-3	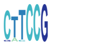	Inf.	c-38	-	-	-
b-11-217-0	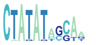	Inf.	c-1550	-	GO:0031326	-
b-6-116-1	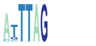	Inf.	c-0	-	-	-
b-6-127-0	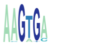	Inf.	c-3	-	-	-
b-8-6-11	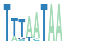	Inf.	c-3	-	-	-
b-7-200-3	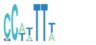	Inf.	c-0	-	-	-
b-6-105-0	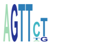	Inf.	c-0	-	-	-
b-8-113-1	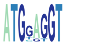	Inf.	c-1542	-	GO:0010646	-
b-6-1-5	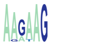	Inf.	c-0	-	-	-
b-6-6-0	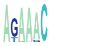	Inf.	c-3	-	-	-
b-12-379-0	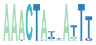	Inf.	c-1555	-	GO:0044260	-
b-12-368-0	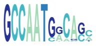	Inf.	c-1558	-	-	-
b-12-214-0	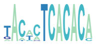	Inf.	c-1535	-	-	-
b-6-148-1	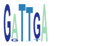	Inf.	c-0	-	-	-
b-8-6-10	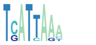	Inf.	c-0	-	-	-
b-8-289-0	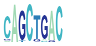	Inf.	c-0	-	-	-
b-7-301-0	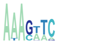	Inf.	c-0	-	-	-
b-12-267-1	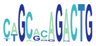	Inf.	c-1543	-	-	-
b-12-246-0	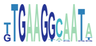	Inf.	c-1526	GO:0003676	GO:0034641	-
b-12-125-0	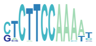	Inf.	c-1551	-	GO:0007275	-

ID	Predicted	z	CBP	Function	Process	Component
b-11-203-0	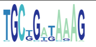	Inf.	c-1562	-	-	GO:0016021
b-12-477-0	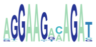	Inf.	c-1539	GO:0004871	GO:0032501	GO:0005886
b-12-345-0	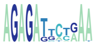	Inf.	c-1555	-	GO:0044260	-
b-12-444-0	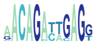	Inf.	c-1553	GO:0004871	GO:0051239	GO:0044459
b-12-312-0	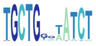	Inf.	c-1545	-	GO:0009653	GO:0005634
b-12-400-0	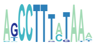	Inf.	c-1541	GO:0005509	-	-
b-7-267-0	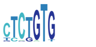	Inf.	c-0	-	-	-
b-7-124-0	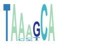	Inf.	c-3	-	-	-
b-11-4-0	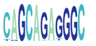	Inf.	c-3	-	-	-
b-6-146-1	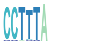	Inf.	c-0	-	-	-
b-6-158-0	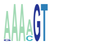	Inf.	c-0	-	-	-
b-7-200-0	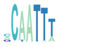	Inf.	c-0	-	-	-
b-8-221-0	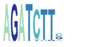	Inf.	c-3	-	-	-
b-11-256-0	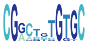	Inf.	c-1535	-	-	-
b-11-124-0	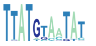	Inf.	c-3	-	-	-
b-12-376-0	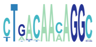	Inf.	c-1341	-	-	-
b-12-464-0	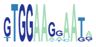	Inf.	c-1548	-	GO:0032502	GO:0005886
b-8-1-0	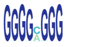	Inf.	c-0	-	-	-
b-6-102-0	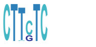	Inf.	c-0	-	-	-
b-7-221-0	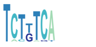	Inf.	c-0	-	-	-
b-12-166-0	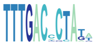	Inf.	c-1557	GO:0001071	GO:0009653	GO:0005634
b-12-452-0	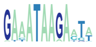	Inf.	c-1550	-	GO:0031326	-
b-6-121-2	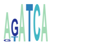	Inf.	c-0	-	-	-
b-7-230-2	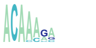	Inf.	c-0	-	-	-
b-6-0-1	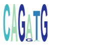	Inf.	c-0	-	-	-
b-7-0-0	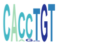	Inf.	c-6	-	-	-
b-7-28-8	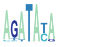	Inf.	c-0	-	-	-
b-6-143-1	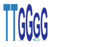	Inf.	c-57	-	-	-

ID	Predicted	z	CBP	Function	Process	Component
b-7-142-1	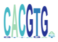	Inf.	c-0	-	-	-
b-7-143-0	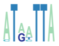	Inf.	c-0	-	-	-
b-8-296-0	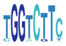	Inf.	c-1536	-	-	-
b-7-230-1	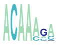	Inf.	c-0	-	-	-
b-7-110-0	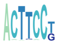	Inf.	c-0	-	-	-
b-5-0-1	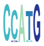	Inf.	c-0	-	-	-
b-12-263-0	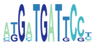	Inf.	c-1536	-	-	-
b-7-241-0	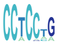	Inf.	c-0	-	-	-
b-11-252-0	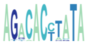	Inf.	c-1341	-	-	-
b-12-471-0	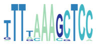	Inf.	c-1543	-	-	-
b-7-182-3	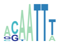	Inf.	c-0	-	-	-
b-6-197-0	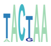	Inf.	c-56	-	-	-
b-8-184-0	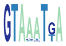	Inf.	c-0	-	-	-
b-7-262-0	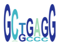	Inf.	c-0	-	-	-
b-8-294-0	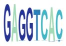	Inf.	c-113	-	-	-
b-11-130-0	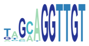	Inf.	c-1341	-	-	-
b-12-140-0	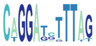	Inf.	c-1543	-	-	-
b-7-282-0	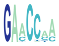	Inf.	c-3	-	-	-
b-10-185-0	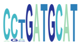	Inf.	c-1541	GO:0005509	-	-
b-7-14-6	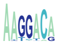	Inf.	c-0	-	-	-
b-6-19-0	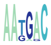	Inf.	c-0	-	-	-
b-7-181-0	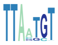	Inf.	c-0	-	-	-
b-8-290-0	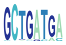	Inf.	c-1562	-	-	GO:0016021
b-8-13-23	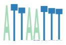	Inf.	c-1546	-	GO:0007155	GO:0016021
b-11-28-0	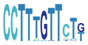	Inf.	c-1545	-	GO:0009653	GO:0005634
b-7-47-4	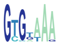	Inf.	c-3	-	-	-
b-6-37-2	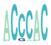	Inf.	c-3	-	-	-
b-11-170-0	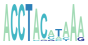	Inf.	c-1549	GO:0001071	GO:0006357	GO:0005634

ID	Predicted	z	CBP	Function	Process	Component
b-6-191-0	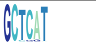	Inf.	c-0	-	-	-
b-7-190-0	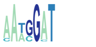	Inf.	c-39	-	-	-
b-5-47-1	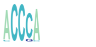	Inf.	c-3	-	-	-
b-7-53-8	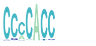	Inf.	c-0	-	-	-
b-12-28-0	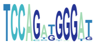	Inf.	c-1341	-	-	-
b-11-49-0	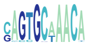	Inf.	c-1545	-	GO:0009653	GO:0005634
b-12-17-0	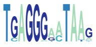	Inf.	c-0	-	-	-
b-7-26-1	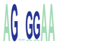	Inf.	c-0	-	-	-
b-6-24-2	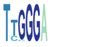	Inf.	c-3	-	-	-
b-6-37-0	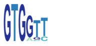	Inf.	c-3	-	-	-
b-5-14-0	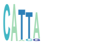	Inf.	c-1367	-	-	GO:0044459
b-11-36-1	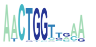	Inf.	c-1341	-	-	-
b-5-46-0	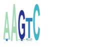	Inf.	c-0	-	-	-
b-11-58-0	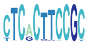	Inf.	c-1542	-	GO:0010646	-
b-6-68-0	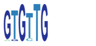	Inf.	c-0	-	-	-
b-7-14-0	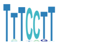	Inf.	c-0	-	-	-
b-12-69-0	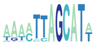	Inf.	c-1546	-	GO:0007155	GO:0016021
b-8-52-7	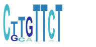	Inf.	c-0	-	-	-
b-11-79-0	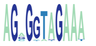	Inf.	c-1539	GO:0004871	GO:0032501	GO:0005886
b-11-24-0	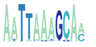	Inf.	c-1539	GO:0004871	GO:0032501	GO:0005886
b-8-13-1	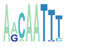	Inf.	c-3	-	-	-
b-7-68-0	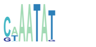	Inf.	c-57	-	-	-
b-6-78-0	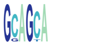	Inf.	c-0	-	-	-
b-5-55-0	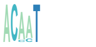	Inf.	c-3	-	-	-
b-5-22-0	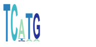	Inf.	c-0	-	-	-
b-8-99-3	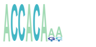	Inf.	c-0	-	-	-
b-8-21-4	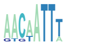	Inf.	c-0	-	-	-
b-6-53-2	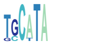	Inf.	c-5	-	-	-

ID	Predicted	z	CBP	Function	Process	Component
b-6-99-0	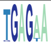	Inf.	c-38	-	-	-
b-12-78-0	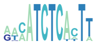	Inf.	c-1341	-	-	-
b-11-99-0	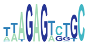	Inf.	c-1562	-	-	GO:0016021
b-11-66-0	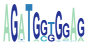	Inf.	c-1535	-	-	-
b-8-52-5	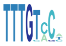	Inf.	c-1526	GO:0003676	GO:0034641	-
b-7-76-2	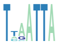	Inf.	c-1546	-	GO:0007155	GO:0016021
b-8-33-2	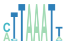	Inf.	c-3	-	-	-
b-9-36-0	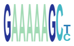	Inf.	c-1526	GO:0003676	GO:0034641	-
b-6-76-0	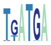	Inf.	c-39	-	-	-
b-6-10-0	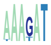	Inf.	c-0	-	-	-
b-7-20-2	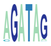	Inf.	c-0	-	-	-
b-11-75-0	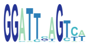	Inf.	c-1341	-	-	-
b-11-53-0	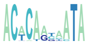	Inf.	c-1341	-	-	-
b-8-52-3	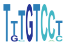	Inf.	c-0	-	-	-
b-6-97-0	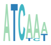	Inf.	c-0	-	-	-
b-12-75-0	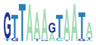	Inf.	c-1539	GO:0004871	GO:0032501	GO:0005886
b-11-96-0	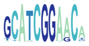	Inf.	c-1535	-	-	-
b-7-62-2	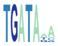	Inf.	c-1556	-	-	-
b-7-42-0	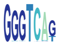	Inf.	c-0	-	-	-
b-15-22-0	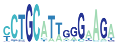	Inf.	c-1541	GO:0005509	-	-
b-11-51-0	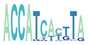	Inf.	c-1536	-	-	-
b-7-4-29	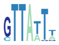	Inf.	c-3	-	-	-
b-8-2-39	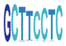	Inf.	c-1542	-	GO:0010646	-
b-7-60-3	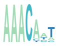	Inf.	c-6	-	-	-
b-8-75-0	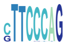	Inf.	c-1548	-	GO:0032502	GO:0005886
b-6-70-3	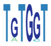	Inf.	c-0	-	-	-
b-6-82-2	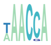	Inf.	c-0	-	-	-
b-5-60-0	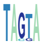	Inf.	c-0	-	-	-

ID	Predicted	z	CBP	Function	Process	Component
b-8-5-18	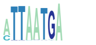	200.98	c-1718	-	-	-
b-7-86-0	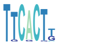	200.98	c-1718	-	-	-
b-8-158-7	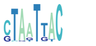	189.71	c-1731	-	-	-
b-12-333-0	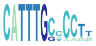	189.71	c-1730	-	-	-
b-7-292-0	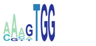	189.71	c-1731	-	-	-
b-5-54-0	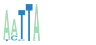	182.7	c-1736	-	GO:0032501	-
b-7-236-1	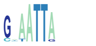	158.29	c-1767	-	-	-
b-7-136-1	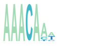	158.29	c-1766	-	-	-
b-8-175-1	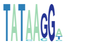	158.08	c-1771	-	-	-
b-7-129-0	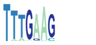	129.35	c-1825	-	GO:0023033	GO:0005575
b-12-419-0	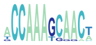	126.46	c-1832	-	-	-
b-12-512-0	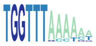	126.46	c-1832	-	-	-
b-12-342-0	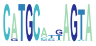	126.46	c-1832	-	-	-
b-6-143-0	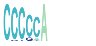	119.8	c-1838	-	-	-
b-7-192-1	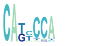	119.8	c-1838	-	-	-
b-6-27-0	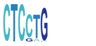	119.8	c-1838	-	-	-
b-7-53-6	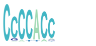	119.8	c-1838	-	-	-
b-7-170-0	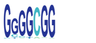	100.35	c-1892	-	-	-
b-5-59-1	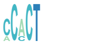	95.72	c-1905	-	-	-
b-6-62-1	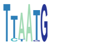	94.95	c-1912	-	GO:0043170	-
b-11-3-0	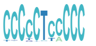	91.68	c-1916	-	GO:0032502	-
b-7-17-4	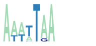	88.12	c-1928	-	-	GO:0005737
b-6-20-5	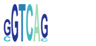	88.12	c-1925	GO:0001071	-	GO:0005634
b-7-18-0	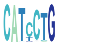	84.95	c-1935	GO:0005509	GO:0007155	GO:0005886
b-6-4-2	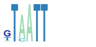	82.1	c-1945	-	-	-
b-6-66-1	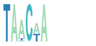	82.1	c-1947	-	GO:0031323	GO:0005576
b-11-1-0	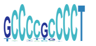	77.19	c-1962	-	GO:0051704	GO:0043234
b-11-73-0	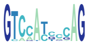	70.22	c-1993	GO:0004871	-	-

ID	Predicted	z	CBP	Function	Process	Component
b-7-182-0	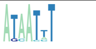	67.99	c-2003	-	GO:0007166	GO:0044459
b-6-20-0	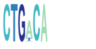	67.99	c-2001	GO:0003677	GO:0006355	-
b-6-10-2	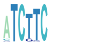	63.86	c-2031	-	-	-
b-7-239-0	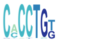	61.39	c-2053	-	-	-
b-10-129-0	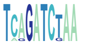	59.78	c-2063	GO:0030528	GO:0000122	GO:0005634
b-11-286-0	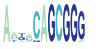	59.78	c-2063	GO:0030528	GO:0000122	GO:0005634
b-7-177-0	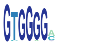	58.42	c-2077	-	-	-
b-8-311-0	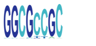	53.46	c-2120	-	-	-
b-7-59-0	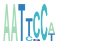	53.46	c-2122	-	GO:0031323	-
b-12-332-0	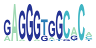	51.69	c-2144	-	GO:0008284	-
b-8-31-2	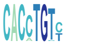	51.69	c-2144	-	GO:0008284	-
b-7-191-0	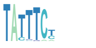	47.32	c-2196	-	-	-
b-6-2-1	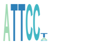	44.33	c-2244	-	-	-
b-7-146-0	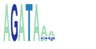	43.32	c-2265	-	GO:0044267	-
b-5-23-0	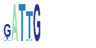	40.13	c-2330	-	-	-
b-7-92-0	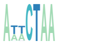	37.76	c-2402	GO:0003677	GO:0009653	-
b-7-43-6	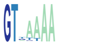	37.36	c-2417	-	-	-
b-6-75-0	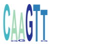	36.85	c-2434	-	-	GO:0005886
b-6-59-1	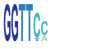	34.77	c-2499	-	-	-
b-10-190-0	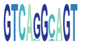	33.91	c-2531	GO:0003676	GO:0007275	-
b-6-76-2	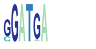	33.84	c-2536	-	-	-
b-7-165-6	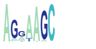	33.54	c-2547	-	-	-
b-8-236-0	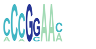	33.47	c-2548	-	-	-
b-6-26-0	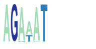	32.1	c-2620	GO:0003674	-	-
b-9-206-0	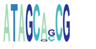	32	c-2627	-	GO:0032502	-
b-12-251-0	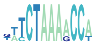	31.74	c-2641	-	GO:0009653	GO:0005634
b-7-182-5	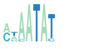	29.45	c-2771	GO:0016787	GO:0042221	GO:0032991
b-6-145-0	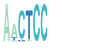	28.01	c-2862	-	GO:0007275	-

ID	Predicted	z	CBP	Function	Process	Component
b-9-42-0	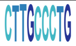	27.42	c-2908	-	GO:0048856	GO:0005737
b-6-87-1	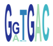	27.38	c-2910	GO:0016787	-	-
b-7-5-10	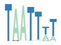	27.03	c-2936	GO:0001071	GO:0006355	GO:0005634
b-7-92-1	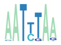	27.01	c-2940	GO:0003677	-	-
b-8-6-3	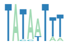	26.3	c-3013	-	-	-
b-7-39-2	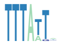	26.02	c-3042	GO:0001071	GO:0006355	-
b-6-115-0	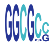	25.18	c-3111	-	-	GO:0005886
b-7-130-1	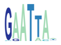	24.81	c-3151	-	GO:0048523	-
b-6-58-0	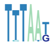	23.83	c-3282	-	GO:0006810	-
b-6-123-1	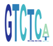	23.72	c-3292	-	-	GO:0005634
b-7-300-0	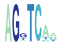	22.95	c-3393	-	-	-
b-6-22-2	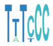	22.17	c-3500	-	-	-
b-6-1-6	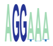	21.91	c-3550	-	-	-
b-12-519-0	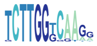	21.68	c-3587	GO:0000166	-	GO:0043234
b-8-95-0	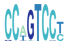	21.24	c-3661	GO:0001071	GO:0032502	GO:0005634
b-8-165-0	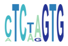	21.24	c-3661	GO:0001071	GO:0032502	GO:0005634
b-12-24-0	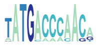	21.24	c-3661	GO:0001071	GO:0032502	GO:0005634
b-7-21-2	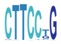	19.62	c-3974	-	-	-
b-6-56-0	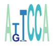	19.44	c-4011	GO:0004871	-	-
b-7-35-2	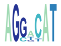	19.16	c-4082	-	GO:0007275	-
b-7-1-2	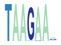	18.9	c-4149	GO:0003677	GO:0048519	-
b-8-13-9	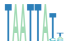	18.79	c-4179	GO:0001071	GO:0006357	GO:0005634
b-12-37-0	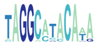	18.42	c-4264	-	GO:0007275	-
b-7-302-0	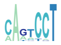	18.21	c-4329	-	-	-
b-6-55-0	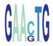	18.2	c-4332	GO:0016788	GO:0007165	GO:0044424
b-8-95-1	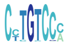	18.14	c-4343	-	-	GO:0044421
b-8-83-4	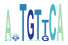	18.1	c-4354	-	-	-
b-6-117-0	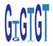	17.38	c-4591	-	-	-

ID	Predicted	z	CBP	Function	Process	Component
b-6-169-0	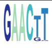	17.17	c-4671	-	-	-
b-12-285-0	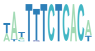	17.12	c-4681	-	-	-
b-6-35-3	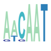	16.98	c-4726	-	-	-
b-7-198-0	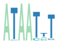	16.76	c-4803	-	-	-
b-7-134-1	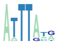	16.63	c-4850	-	-	GO:0005737
b-11-161-0	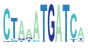	16.35	c-4944	-	-	-
b-6-52-0	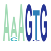	16.34	c-4956	-	GO:0019219	-
b-8-160-0	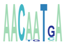	16.32	c-4968	-	-	-
b-6-126-0	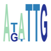	15.45	c-5322	-	GO:0048856	-
b-7-227-0	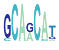	15.01	c-5525	GO:0001071	GO:0009653	GO:0005634
b-12-33-0	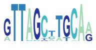	14.57	c-5735	-	-	-
b-6-74-1	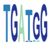	14.44	c-5807	GO:0005509	-	-
b-7-205-0	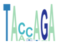	14.37	c-5853	GO:0008092	GO:0007389	GO:0005737
b-8-288-0	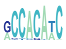	14.33	c-5879	-	-	-
b-8-184-1	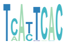	14.33	c-5879	-	-	-
b-7-164-0	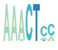	14.33	c-5879	-	-	-
b-12-262-0	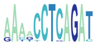	14.15	c-5996	GO:0005488	-	-
b-6-47-0	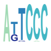	14.09	c-6026	-	-	-
b-15-239-0	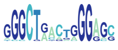	13.65	c-6292	-	-	-
b-7-113-2	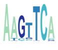	13.61	c-6308	-	GO:0048584	GO:0044459
b-7-189-0	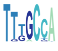	13.61	c-6308	-	GO:0048584	GO:0044459
b-12-184-0	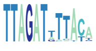	13.61	c-6308	-	GO:0048584	GO:0044459
b-7-201-0	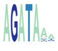	13.55	c-6350	-	GO:0045449	-
b-7-60-2	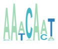	13.44	c-6441	-	GO:0048856	GO:0005576
b-7-61-2	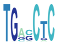	13.4	c-6469	-	GO:0048523	-
b-8-121-0	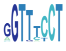	13.38	c-6476	GO:0001071	-	GO:0005634
b-6-6-1	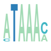	13.26	c-6578	GO:0003677	-	-
b-12-19-0	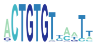	13.16	c-6644	GO:0004872	GO:0071842	GO:0044421

ID	Predicted	z	CBP	Function	Process	Component
b-7-106-0	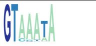	13.15	c-6656	GO:0005524	GO:0044260	-
b-7-43-0	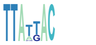	12.96	c-6780	-	GO:0007165	-
b-7-12-11	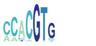	12.81	c-6892	-	GO:0022414	-
b-6-1-7	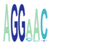	12.77	c-6919	-	-	-
b-7-210-3	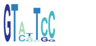	12.41	c-7193	GO:0016788	GO:0042592	-
b-12-283-0	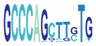	12.41	c-7193	GO:0016788	GO:0042592	-
b-8-194-0	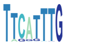	12.32	c-7282	GO:0008270	-	-
b-7-121-0	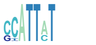	12.3	c-7298	GO:0001071	GO:0048856	GO:0005615
b-6-87-2	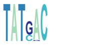	12.05	c-7498	-	GO:0010604	-
b-7-290-0	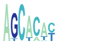	12.05	c-7501	GO:0003676	-	-
b-8-84-3	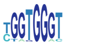	11.98	c-7563	-	-	-
b-7-202-1	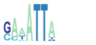	11.67	c-7870	GO:0001071	GO:0032502	GO:0005634
b-8-201-4	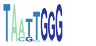	11.66	c-7883	-	-	-
b-7-245-0	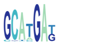	11.66	c-7883	-	-	-
b-7-190-1	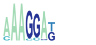	11.64	c-7904	-	GO:0009056	-
b-8-13-18	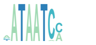	11.61	c-7934	GO:0005509	GO:0048869	GO:0000267
b-7-37-2	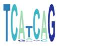	11.51	c-8036	-	GO:0048856	-
b-8-33-1	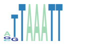	11.5	c-8048	-	GO:0007275	-
b-7-99-0	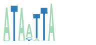	11.38	c-8157	-	-	-
b-11-104-0	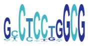	11.35	c-8192	-	GO:0006950	GO:0005576
b-7-149-0	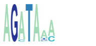	11.24	c-8315	-	GO:0009056	-
b-10-174-0	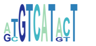	11.24	c-8315	-	GO:0009056	-
b-7-189-2	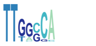	11.12	c-8428	GO:0008270	-	-
b-8-112-0	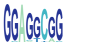	11.05	c-8500	GO:0003676	-	-
b-7-178-0	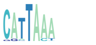	10.97	c-8589	-	-	GO:0016021
b-8-32-0	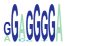	10.96	c-8599	-	-	-
b-7-14-1	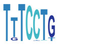	10.89	c-8686	-	-	-
b-6-103-0	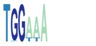	10.73	c-8863	GO:0001071	GO:0010604	GO:0005794

ID	Predicted	z	CBP	Function	Process	Component
b-7-35-6	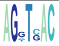	10.72	c-8873	-	-	-
b-12-21-1	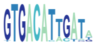	10.68	c-8933	GO:0001071	GO:0051716	GO:0005634
b-6-132-0	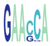	10.58	c-9056	-	-	-
b-12-455-0	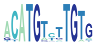	10.53	c-9138	-	GO:0006355	GO:0005634
b-7-7-8	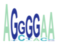	10.52	c-9144	GO:0001071	GO:0003002	GO:0005634
b-8-105-1	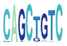	10.48	c-9182	-	-	-
b-8-215-1	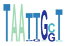	10.4	c-9280	-	GO:0050789	GO:0005739
b-7-242-0	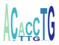	10.36	c-9341	-	GO:0044281	-
b-12-178-0	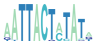	10.34	c-9379	-	-	-
b-8-106-0	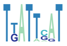	10.33	c-9405	-	GO:0048869	GO:0005794
b-7-123-0	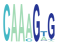	10.33	c-9405	-	GO:0048869	GO:0005794
b-8-22-6	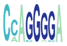	10.17	c-9623	GO:0003677	GO:0048856	GO:0005634
b-8-62-13	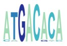	10.14	c-9667	-	-	-
b-6-76-1	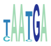	10.13	c-9687	-	-	-
b-8-13-27	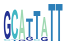	10.09	c-9750	-	-	-
b-12-130-0	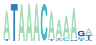	10.04	c-9821	-	-	-
b-10-53-1	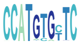	10.03	c-9849	-	GO:0009611	GO:0019898
b-8-217-2	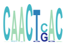	10.02	c-9872	-	-	-
b-8-298-0	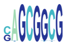	9.95	c-9987	GO:0001071	GO:0006355	-
b-8-24-0	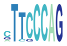	9.95	c-9987	GO:0001071	GO:0006355	-
b-7-229-0	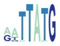	9.91	c-10044	GO:0005102	GO:0006461	GO:0044459
b-7-156-0	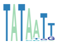	9.91	c-10044	GO:0005102	GO:0006461	GO:0044459
b-7-162-0	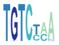	9.91	c-10047	-	GO:0022414	GO:0044428
b-7-39-3	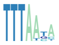	9.91	c-10047	-	GO:0022414	GO:0044428
b-8-39-0	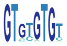	9.84	c-10160	-	-	-
b-7-11-19	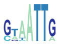	9.84	c-10171	-	-	-
b-8-49-2	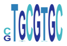	9.77	c-10282	-	GO:0009653	-
b-7-295-0	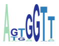	9.77	c-10286	-	-	-

ID	Predicted	z	CBP	Function	Process	Component
b-8-7-1	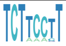	9.71	c-10393	GO:0001071	GO:0007275	GO:0044428
b-8-217-1	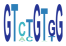	9.7	c-10410	-	-	-
b-7-270-0	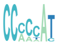	9.7	c-10410	-	-	-
b-7-236-3	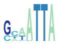	9.63	c-10535	-	GO:0009987	GO:0016021
b-7-73-0	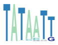	9.63	c-10535	-	GO:0009987	GO:0016021
b-8-168-0	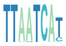	9.6	c-10585	GO:0005515	GO:0009888	-
b-7-236-0	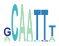	9.6	c-10598	GO:0001071	-	GO:0005634
b-7-291-0	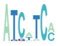	9.59	c-10619	-	-	-
b-7-289-0	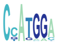	9.56	c-10678	GO:0001071	GO:0006355	GO:0005634
b-8-105-0	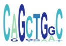	9.55	c-10694	-	GO:0044237	-
b-11-173-0	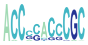	9.5	c-10778	-	-	-
b-7-0-1	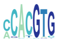	9.46	c-10847	-	-	-
b-15-210-0	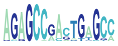	9.42	c-10938	GO:0005198	GO:0048584	GO:0016023
b-7-11-6	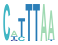	9.4	c-10971	GO:0003677	GO:0007275	-
b-8-120-0	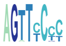	9.36	c-11037	-	-	-
b-7-81-0	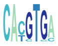	9.36	c-11040	GO:0005509	GO:0007155	-
b-7-0-2	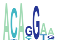	9.34	c-11062	-	-	-
b-8-21-5	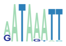	9.34	c-11062	-	-	-
b-7-51-1	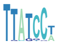	9.34	c-11062	-	-	-
b-7-209-0	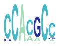	9.2	c-11336	GO:0000166	GO:0023034	-
b-11-59-0	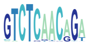	9.18	c-11363	-	-	-
b-8-17-0	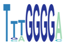	9.17	c-11377	-	-	-
b-7-20-3	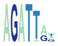	9.16	c-11403	GO:0032553	GO:0023033	-
b-11-56-0	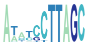	9.11	c-11485	GO:0032553	-	-
b-8-192-3	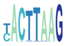	9.07	c-11588	GO:0001071	GO:0048856	-
b-6-116-0	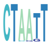	9.05	c-11621	GO:0001071	GO:0010468	GO:0005634
b-7-176-3	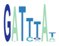	9.05	c-11628	-	-	-
b-8-335-0	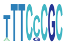	9.04	c-11658	-	GO:0048523	-

ID	Predicted	z	CBP	Function	Process	Component
b-7-24-4	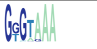	9.03	c-11667	-	GO:0044260	-
b-8-12-5	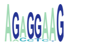	9.03	c-11667	-	GO:0044260	-
b-7-252-0	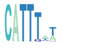	9.03	c-11668	GO:0005509	GO:0008150	-
b-8-28-0	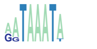	9.03	c-11668	GO:0005509	GO:0008150	-
b-8-216-1	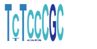	9.02	c-11692	-	-	-
b-11-72-0	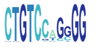	8.89	c-11960	GO:0008270	-	-
b-8-62-9	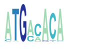	8.82	c-12075	-	-	GO:0005576
b-11-176-0	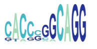	8.82	c-12076	-	-	-
b-7-235-0	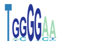	8.77	c-12183	-	GO:0045449	-
b-8-271-0	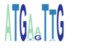	8.7	c-12346	-	-	-
b-11-60-0	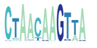	8.64	c-12496	-	GO:0045449	-
b-8-99-5	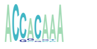	8.56	c-12663	-	-	-
b-7-179-2	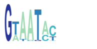	8.53	c-12746	-	GO:0006355	-
b-12-43-0	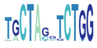	8.5	c-12834	-	-	-
b-7-285-0	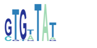	8.5	c-12839	GO:0003677	GO:0009653	GO:0005634
b-7-118-0	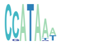	8.44	c-12976	-	GO:0032501	-
b-7-5-9	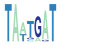	8.41	c-13043	-	GO:0071842	-
b-7-55-5	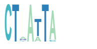	8.39	c-13108	-	-	-
b-8-63-0	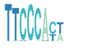	8.35	c-13219	-	GO:0019538	GO:0044459
b-7-121-3	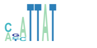	8.29	c-13334	GO:0001071	GO:0044249	-
b-9-85-0	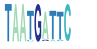	8.28	c-13362	-	GO:0006139	GO:0005886
b-8-212-1	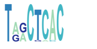	8.28	c-13373	-	GO:0065008	-
b-8-81-0	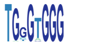	8.26	c-13428	-	GO:0045449	GO:0005634
b-11-13-0	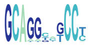	8.26	c-13454	-	-	-
b-8-331-0	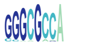	8.18	c-13673	GO:0003674	GO:0032501	-
b-8-173-0	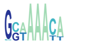	8.17	c-13703	-	GO:0032502	GO:0005575
b-9-119-0	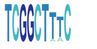	8.15	c-13750	-	GO:0031326	GO:0044459
b-7-55-7	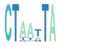	8.13	c-13811	-	-	GO:0005737

ID	Predicted	z	CBP	Function	Process	Component
b-8-283-0	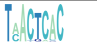	8.11	c-13858	-	GO:0019538	-
b-6-193-0	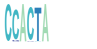	8.11	c-13858	-	GO:0019538	-
b-7-222-0	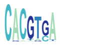	8.02	c-14168	GO:0003677	-	GO:0005634
b-7-215-0	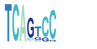	7.99	c-14235	-	GO:0019219	-
b-8-329-0	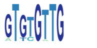	7.99	c-14251	GO:0005102	GO:0009966	GO:0042995
b-6-124-1	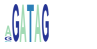	7.99	c-14252	-	-	-
b-11-282-0	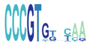	7.98	c-14282	-	-	GO:0005737
b-12-507-0	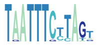	7.98	c-14286	GO:0005102	GO:0065008	-
b-7-68-1	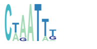	7.96	c-14353	GO:0016788	GO:0009056	-
b-8-305-0	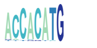	7.86	c-14670	-	-	-
b-8-169-1	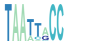	7.8	c-14827	GO:0005509	GO:0045184	GO:0005730
b-10-22-1	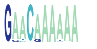	7.79	c-14871	-	-	-
b-12-406-0	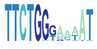	7.79	c-14878	-	-	GO:0005886
b-7-87-1	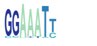	7.77	c-14922	-	-	-
b-8-227-0	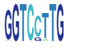	7.75	c-14994	GO:0001071	GO:0006355	GO:0005634
b-6-210-0	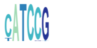	7.73	c-15045	-	-	-
b-8-287-0	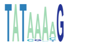	7.7	c-15149	-	-	-
b-8-30-6	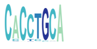	7.65	c-15296	-	GO:0048856	-
b-8-206-0	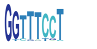	7.55	c-15630	-	GO:0048856	-
b-8-318-0	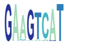	7.53	c-15734	GO:0000988	-	-
b-12-38-0	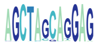	7.5	c-15833	-	GO:0008152	-
b-7-138-0	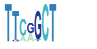	7.39	c-16199	-	-	GO:0005576
b-8-28-12	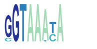	7.36	c-16290	GO:0043565	GO:0006355	-
b-12-259-0	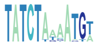	7.28	c-16591	GO:0001071	GO:0048856	GO:0005634
b-7-87-4	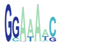	7.28	c-16591	GO:0001071	GO:0048856	GO:0005634
b-7-72-0	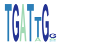	7.25	c-16737	-	-	GO:0005886
b-8-273-0	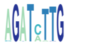	7.23	c-16825	GO:0001071	GO:0010033	GO:0005634
b-7-55-10	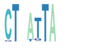	7.22	c-16848	-	-	-

ID	Predicted	z	CBP	Function	Process	Component
b-11-114-0	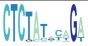	7.2	c-16908	-	GO:0006629	-
b-6-138-1	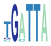	7.19	c-16952	GO:0046982	GO:0044057	GO:0005625
b-11-33-0	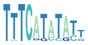	7.19	c-16967	GO:0016788	GO:0006281	-
b-15-59-0	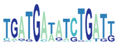	7.18	c-16998	GO:0022836	GO:0015672	GO:0034702
b-11-132-0	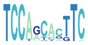	7.18	c-16999	-	-	-
b-8-291-0	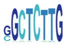	7.17	c-17034	-	GO:0048856	-
b-7-89-3	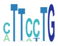	7.14	c-17175	-	-	-
b-7-4-28	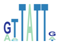	7.11	c-17290	-	-	-
b-12-438-0	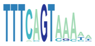	7.11	c-17312	-	-	-
b-11-25-0	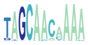	7.07	c-17436	GO:0005215	GO:0071842	-
b-12-108-0	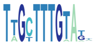	7.05	c-17503	-	GO:0006464	GO:0005737
b-12-175-0	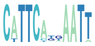	7.05	c-17534	GO:0001071	GO:0009653	-
b-10-88-1	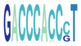	7.03	c-17605	-	-	GO:0043234
b-11-81-0	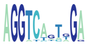	7	c-17739	GO:0003677	GO:0048523	GO:0005634
b-7-132-2	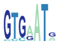	6.9	c-18134	GO:0004871	GO:0032501	GO:0005887
b-7-250-0	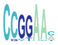	6.84	c-18398	-	GO:0006139	GO:0005634
b-8-5-15	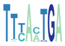	6.78	c-18658	GO:0003682	GO:0032868	GO:0044427
b-7-12-7	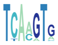	6.7	c-19018	GO:0001071	GO:0006355	-
b-11-15-0	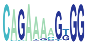	6.7	c-19046	GO:0001071	GO:0006355	-
b-8-151-0	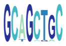	6.63	c-19373	-	-	-
b-11-0-0	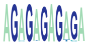	6.62	c-19430	GO:0043565	GO:0001525	GO:0016021
b-8-195-1	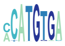	6.58	c-19592	-	GO:0032502	-
b-10-193-0	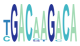	6.58	c-19599	GO:0001071	GO:0009653	GO:0005634
b-8-241-1	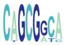	6.57	c-19628	-	-	-
b-8-345-0	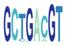	6.54	c-19751	-	-	-
b-10-63-0	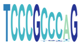	6.53	c-19818	-	GO:0045449	-
b-7-28-9	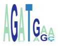	6.44	c-20275	GO:0005525	GO:0007017	GO:0005874
b-8-214-0	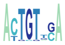	6.38	c-20583	GO:0003677	GO:0048869	-

ID	Predicted	z	CBP	Function	Process	Component
b-12-360-0	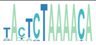	6.35	c-20736	GO:0003677	GO:0006355	GO:0005622
b-12-177-0	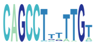	6.34	c-20790	-	GO:0051049	-
b-8-242-0	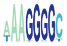	6.34	c-20814	GO:0022857	GO:0006812	GO:0031090
b-8-256-1	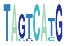	6.32	c-20917	GO:0043565	GO:0048513	GO:0005634
b-8-132-0	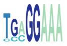	6.31	c-20960	GO:0005254	GO:0048704	GO:0043005
b-7-62-3	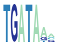	6.27	c-21170	-	-	GO:0005737
b-11-55-0	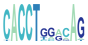	6.25	c-21307	-	-	GO:0044459
b-12-221-0	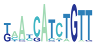	6.24	c-21350	-	-	GO:0044459
b-8-247-0	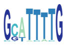	6.15	c-21874	-	-	-
b-11-269-0	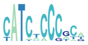	6.11	c-22067	GO:0004871	-	GO:0044425
b-7-4-24	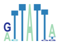	6.1	c-22162	-	GO:0007155	GO:0005886
b-9-75-0	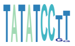	5.98	c-22912	GO:0016740	GO:0045449	GO:0044428
b-8-263-1	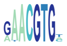	5.93	c-23192	GO:0003924	GO:0043523	GO:0043025
b-8-333-0	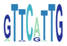	5.91	c-23352	-	GO:0043412	GO:0016020
b-7-98-2	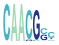	5.86	c-23668	-	-	-
b-11-221-0	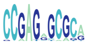	5.84	c-23778	-	-	-
b-7-251-0	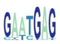	5.76	c-24325	GO:0005488	-	-
b-8-330-0	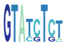	5.57	c-25626	-	GO:0045944	GO:0043234
b-8-12-0	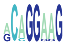	5.47	c-26367	GO:0032553	GO:0035556	-
b-12-415-0	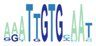	5.36	c-27323	-	GO:0032502	-
b-8-125-0	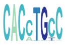	5.24	c-28328	GO:0022892	GO:0006915	GO:0031090
b-12-420-0	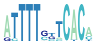	5.21	c-28620	GO:0019899	GO:2000026	GO:0005634
b-8-245-0	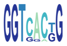	5.2	c-28680	GO:0005509	GO:0009653	-
b-8-187-1	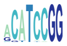	5.2	c-28689	GO:0005267	GO:0006813	GO:0044433
b-11-107-0	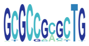	5.14	c-29204	GO:0022892	GO:0007267	GO:0030054
b-10-16-2	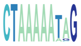	5.11	c-29462	GO:0004725	GO:0006470	GO:0042995
b-11-95-0	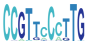	5.04	c-30173	-	GO:0009058	-
b-8-62-3	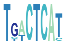	4.93	c-31208	-	-	-

ID	Predicted	z	CBP	Function	Process	Component
b-12-67-0	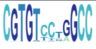	4.85	c-31944	GO:0005509	GO:0048856	GO:0005882
b-12-515-0	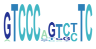	4.85	c-32014	GO:0005509	GO:0032787	GO:0044421
b-8-279-0	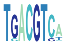	4.77	c-32855	GO:0001071	GO:0016070	-
b-8-2-45	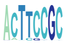	4.76	c-32908	-	-	-
b-9-14-1	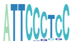	4.76	c-32982	-	GO:0071842	GO:0044459
b-12-211-0	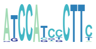	4.75	c-33048	-	-	-
b-8-202-0	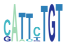	4.65	c-34099	-	GO:0065008	GO:0044459
b-9-52-0	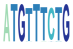	4.6	c-34641	GO:0001071	GO:0032502	-
b-12-64-0	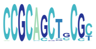	4.58	c-34857	-	-	-
b-12-53-0	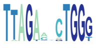	4.44	c-36544	GO:0001071	GO:0009888	GO:0044427
b-12-392-0	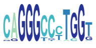	4.4	c-37137	GO:0001883	-	-
b-7-133-0	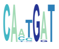	4.36	c-37566	-	-	-
b-8-159-0	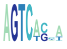	4.36	c-37633	-	-	-
b-8-344-0	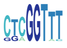	4.34	c-37855	-	-	-
b-10-36-0	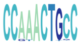	4.3	c-38300	GO:0005515	GO:0002376	-
b-8-253-0	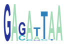	4.29	c-38508	-	-	-
b-8-70-0	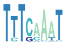	4.19	c-39733	-	-	-
b-15-240-0	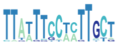	4.05	c-41768	-	-	-
b-12-183-0	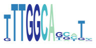	4.04	c-41905	-	-	-
b-8-275-0	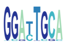	4.03	c-41960	-	-	-
b-12-222-1	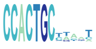	3.94	c-43359	-	GO:0010604	-
b-12-399-1	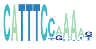	3.83	c-44943	GO:0043565	GO:0048856	GO:0005634
b-12-181-0	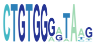	3.83	c-44951	-	GO:0071842	GO:0005886
b-11-264-0	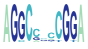	3.79	c-45646	-	-	-
b-6-211-0	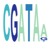	3.74	c-46322	GO:0017076	GO:0048856	-
b-11-4-1	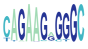	3.5	c-50493	-	-	-
b-12-405-0	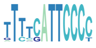	3.46	c-51102	-	-	-
b-8-143-0	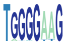	3.39	c-52391	-	-	-

ID	Predicted	z	CBP	Function	Process	Component
b-12-55-0	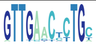	3.33	c-53515	-	-	-
b-15-246-0	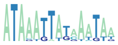	3.31	c-53912	-	-	-
b-8-325-1	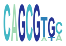	3.15	c-56953	-	-	-
b-8-352-0	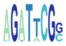	3.09	c-58124	-	-	-

S2.5 CBP gallery

We selected a few CBPs for display in this gallery according to the following criteria:

- The CBPs applies to more than 10 genes.
- SIG is greater than 3.0.
- At least one of our *ab initio* TFBM predictions are included in the CBP.

The remaining 150000+ visualizations can be downloaded from the provided repository: http://computational-biology.mpi-muenster.mpg.de/publications/TFBM/.

In case the CBP has significant ontologies, they are listed in each following row. We explained in section S1.5.1 how to interpret the visualizations.

Table S9: CBP Gallery. In case the CBP has significant ontologies, they are listed in each following row.

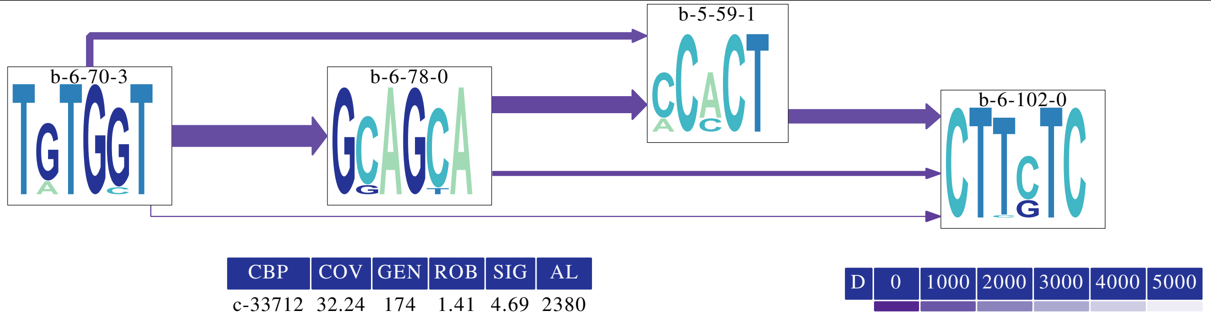

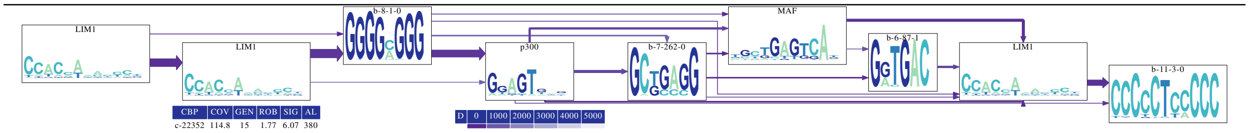

Nucleic acid binding transcription factor activity, anatomical structure morphogenesis, intracellular membrane-bounded organelle

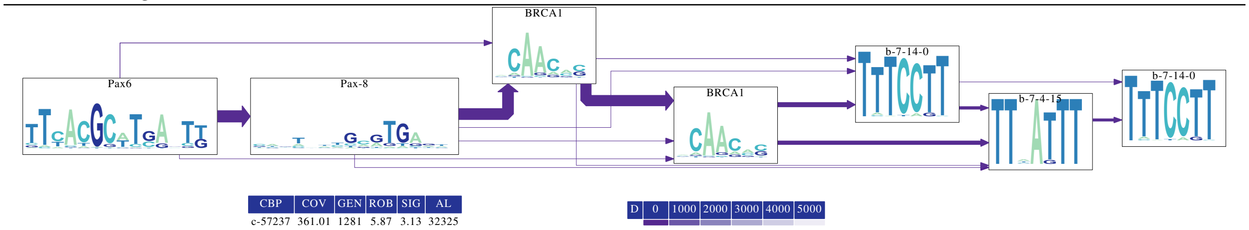

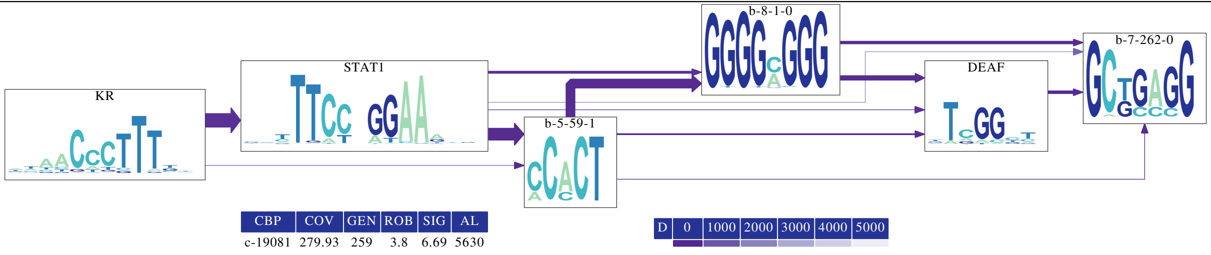

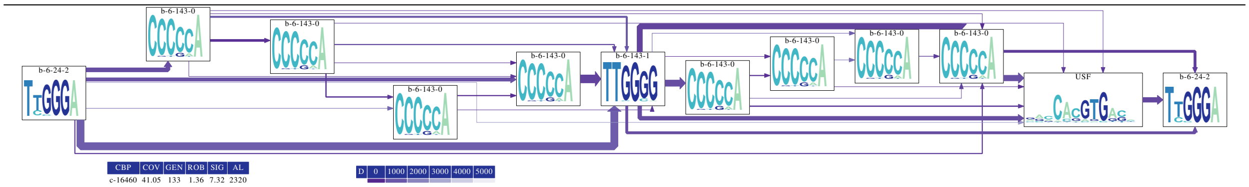

Positive regulation of cellular process

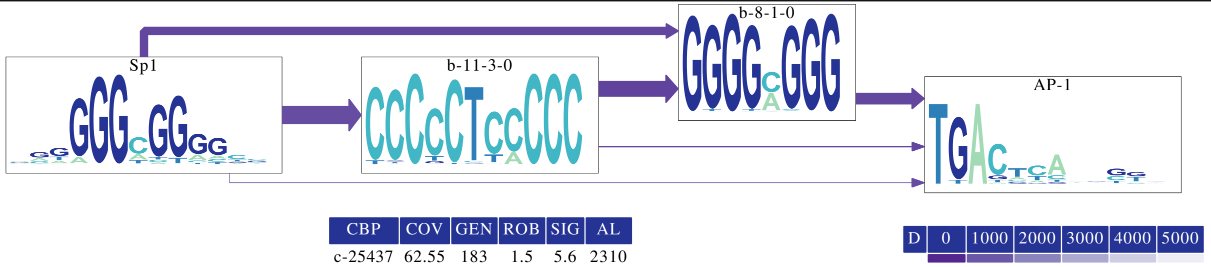

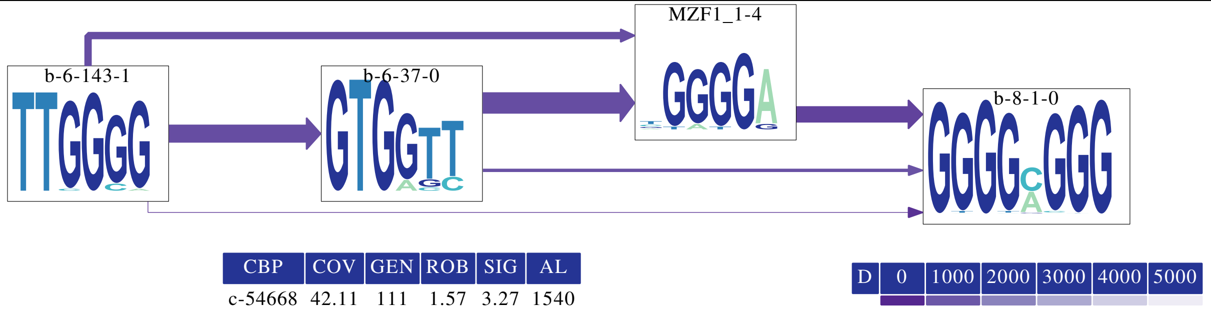

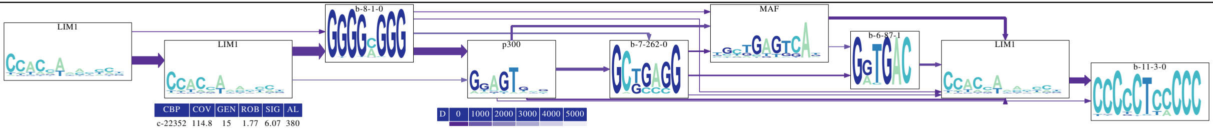

Nucleic acid binding transcription factor activity, anatomical structure morphogenesis, intracellular membrane-bounded organelle

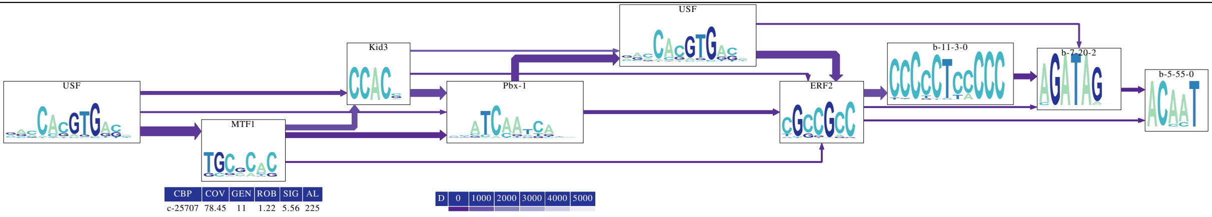

Nucleic acid binding transcription factor activity, pattern specification process, nucleus

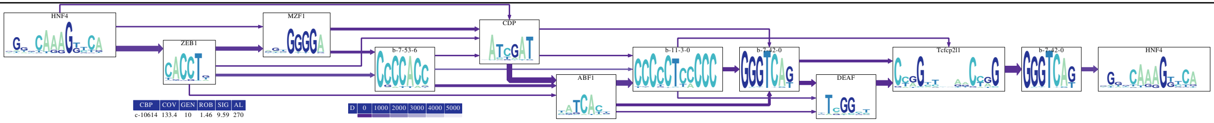

Nucleic acid binding transcription factor activity, regulation of transcription, nucleus

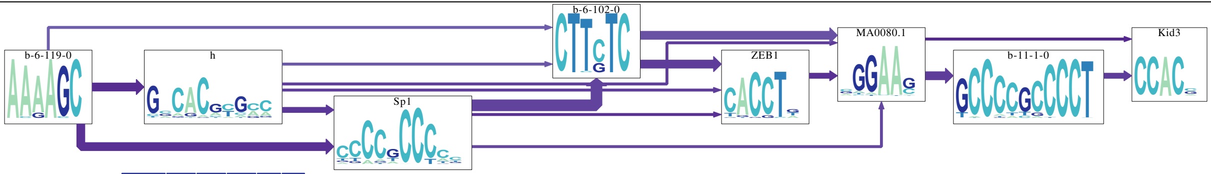

CBP	COV	GEN	ROB	SIG	AL
c-11160	77.8	10	1.31	9.3	170

D	0	1000	2000	3000	4000	5000

Sequence-specific DNA binding, organ development

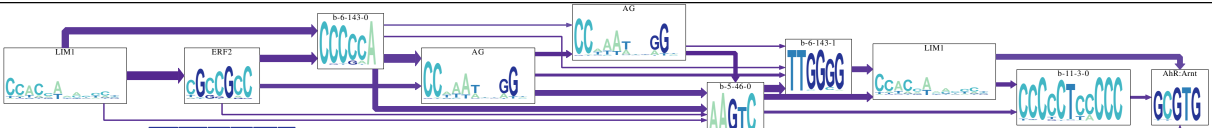

CBP	COV	GEN	ROB	SIG	AL
c-8738	100.82	17	1.53	10.85	390

D	0	1000	2000	3000	4000	5000

Nucleic acid binding transcription factor activity, developmental process, nucleus

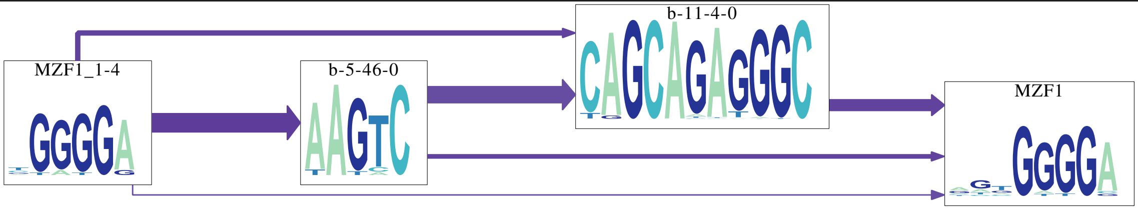

CBP	COV	GEN	ROB	SIG	AL
c-35805	42.86	98	1.53	4.5	1320

D	0	1000	2000	3000	4000	5000

Purine nucleotide binding

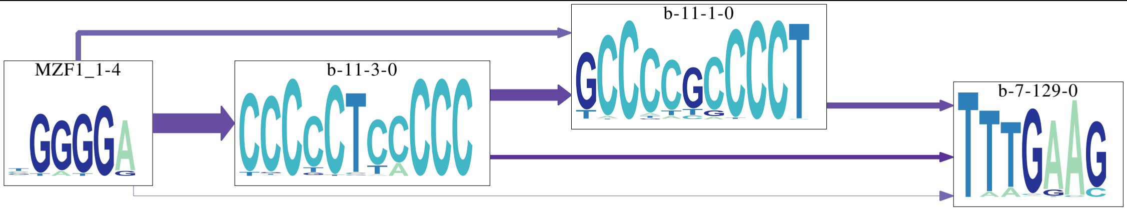

CBP	COV	GEN	ROB	SIG	AL
c-51614	46.68	57	1.39	3.44	700

D	0	1000	2000	3000	4000	5000

Table S10: Set of data files provided in our catalog. There are several types of data sources. The type "files" implies a set of files. The type "file" refers to only one file. The type "dir" is a directory with files. Each individual file is an entry in the dataset.

Type	Name	Description
dir	Motif-Predictions	Includes the list of all predicted motifs. Each motif filename has the form "b-$w-$n-$s" where w is the length of the motif, n is the motif group and s is the identification within the given motif group.
files	motif-locations-$w.csv	Genomic locations were motifs from Jaspar, Transfac and our predictions bind. These files were used for CBP generation.
files	predictions-summary-$w.csv	The main component of each prediction group is recorded in each line of this file.
files	predictions-full-$w.csv	Each prediction group is displayed. Entries of the group are stored in each line of this file.
file	CBP-Visualization.zip	Every entry is of the form "c-$id.eps", where id is the CBP identification number.
file	cbps-summary.csv	Summary of CBPs found (motifs and genes).

S3 Description of the supporting datasets

A number of datasets have been created. They can be downloaded from the following website:
`http://computational-biology.mpi-muenster.mpg.de/publications/TFBM/`.

Whenever there is a file for each "DNA word" length w we include "$w", in the filename. There are several types of data sources. The type "files" implies a set of files. The type "file" is just one file. The type "dir" is a directory with files. Table S10 is an overview of the generated datasets. The detailed format of each entry is explained in this section. All the files ending in .csv are tab delimited. The section title always contains the folder name where a list of files is located, separated by forward slash "/".

S3.1 Directory: Motif-Predictions

Each entry of the "Motif-Predictions" directory is a Fasta file. Each "bpred" entry is a binding site sequence of the predicted motif. We have included also a PDF version of the motif in this directory.

S3.2 Files: Locations/motif-locations-$w.csv

Each entry represents a motif match in the genome. The motifs used are those in Jaspar and Transfac alongside our predictions. The following columns are stored in the file:

- Matching window: The sequence where the TF is binding.
- CHR Position: Chromosome position.
- CHR id: Chromosome / species identification string
- Reserved.
- Normalized conservation: The average number of species that share each of the bases of the window.

- Local conservation z-score: The conservation of this window with respect to other locations. NaN if the window exists only in one genome location.
- Global conservation z-score: The number of standard deviations that the conservation departs from the mean conservation of all windows in B_w.
- TF binding energy.
- TF binding energy z-score.
- TF name (our predictions start with "b-", Jaspar's motifs start with "MA-". The other predictions, that have a $ symbol in their identifier, are from the Transfac database).
- NCBI gene name.
- Standard gene name.
- Gene direction.
- Position inside the promoter. The value 0 is just before the beginning of the transcription.

S3.3 Files: Predictions-Summary/predictions-summary-$w.csv

Each line in this file contains the highest ranking element of the motif prediction groups. The first column contains the row id. The second column holds the motif prediction identifier. This identifier is of the form: "b-$w-$n-$s", where w is the length of the motif, n is the motif group, and s is the identification within the given motif group. The third column contains one or more Pearson correlation results for the matching motifs in the fourth column.

S3.4 Files: Predictions-Full/predictions-full-$w.csv

The format of this file is similar to "predictions-summary-$w.csv", except that all the entries of the groups are displayed here.

S3.5 File: CBP-Visualization.zip

This compressed file contains PDF files with all the CBPs found. Every entry is of the form "c-$id.pdf", where id is the CBP identification number.

S3.6 File: CBP/cbps-summary.csv

The summary of each CBP is included in each line of this dataset. The following fields are included in each column:

- CBP ID: CBP identification number
- Motif counts: Number of motifs included in the rule.
- Score: Sum of the alignment scores against the base sequence.
- Significance: The z-score of the actual GEN in the distribution of GEN that would be generated if the promoters are randomly shuffled (section S1.5.2).
- Generality: Number of genes in the CBP.
- Coverage: Number of bases where motifs are binding

- Robustness: Number of times an individual motif is duplicated in the CBP.
- Motif names: One or more columns are the motif names of the CBP
- “|” Separation token. This character divides the previous list from the next list.
- Gene names: Genes where this CBP applies.

References

1. Fujita PA, Rhead B, Zweig AS, Hinrichs AS, Karolchik D, Cline MS, Goldman M, Barber GP, Clawson H, Coelho A, Diekhans M, Dreszer TR, Giardine BM, Harte RA, Hillman-Jackson J, Hsu F, Kirkup V, Kuhn RM, Learned K, Li CH, Meyer LR, Pohl A, Raney BJ, Rosenbloom KR, Smith KE, Haussler D, Kent WJ: **The UCSC genome browser database: update 2011**. *Nucleic Acids Research* 2010, **31**:1–7.

2. Bryne JC, Valen E, Tang MHE, Marstrand T, Winther O, da Piedade I, Krogh A, Lenhard B, Sandelin A: **JASPAR, the open access database of transcription factor binding profiles: new content and tools in the 2008 update**. *Nucleic Acids Research* 2008, **36**(suppl 1):D102–D106.

3. Matys V, Kel-Margoulis OV, Fricke E, Liebich I, Land S, Barre-Dirrie A, Reuter I, Chekmenev D, Krull M, Hornischer K, Voss N, Stegmaier P, Lewicki-Potapov B, Saxel H, Kel AE, Wingender E: **TRANSFAC and its module TRANSCompel: transcriptional gene regulation in eukaryotes**. *Nucleic Acids Research* 2006, **34**(suppl 1):D108–D110.

4. Zezula P, Amato G, Dohnal V, Batko M: *Similarity Search: The Metric Space Approach*. Secaucus, NJ, USA: Springer-Verlag 2005.

5. Samet H: *Foundations of Multidimensional and Metric Data Structures*. San Francisco, CA, USA: Morgan Kaufmann Publishers Inc. 2005.

6. Hjaltason GR, Samet H: **Index-driven similarity search in metric spaces**. *ACM Trans. Database Syst.* 2003, **28**(4):517–580.

7. Chavez E, Navarro G, Baeza-Yates R, Marroquin JL: **Searching in metric spaces**. *ACM Comput. Surv.* 2001, **33**(3):273–321.

8. Müller-Molina AJ, Shinohara T: **Efficient similarity search by reducing I/O with compressed sketches**. In *SISAP*, IEEE 2009:30–38.

9. Müller-Molina AJ: **OBSearch: a high performance similarity search engine for Java**. In *Proceedings of the 2009 Second International Workshop on Similarity Search and Applications*, SISAP '09, Washington, DC, USA: IEEE Computer Society 2009:143–145.

10. Deza MM, Deza E: *Encyclopedia of Distances*. Berlin Heidelberg: Springer 2009.

11. Xie X, Lu J, Kulbokas EJ, Golub TR, Mootha V, Lindblad-Toh K, Lander ES, Kellis M: **Systematic discovery of regulatory motifs in human promoters and 3' UTRs by comparison of several mammals**. *Nat.* 2005, **434**(7031):338–345.

12. Stormo GD: **DNA binding sites: representation and discovery**. *Bioinformatics* 2000, **16**:16–23.

13. `http://commons.apache.org/math/`.

14. Esuli A: **Use of permutation prefixes for efficient and scalable approximate similarity search**. Amsterdam, Netherlands 2011, [http://www.sciencedirect.com/science/article/pii/S0306457310001019].

15. Sadit E, Chávez E, Navarro G: **Succinct Nearest Neighbor Search**. In *Proc. 4th International Workshop on Similarity Search and Applications (SISAP)*, ACM Press 2011.

16. Amato G, Savino P: **Approximate Similarity Search in Metric Spaces Using Inverted Files**. In *Proceedings of the 3rd international conference on Scalable information systems*, InfoScale '08, ICST, Brussels, Belgium, Belgium: ICST (Institute for Computer Sciences, Social-Informatics and Telecommunications Engineering) 2008:28:1–28:10, [http://portal.acm.org/citation.cfm?id=1459693.1459731].

17. McLean CY, Reno PL, Pollen AA, Bassan AI, Capellini TD, Guenther C, Indjeian VB, Lim X, Menke DB, Schaar BT, Wenger AM, Bejerano G, Kingsley DM: **Human-specific loss of regulatory DNA and the evolution of human-specific traits**. *Nature* 2011, **471**:216–219.

18. Tung AKH, Zhang R, Koudas N, Ooi BC: **Similarity search: a matching based approach**. In *Proceedings of the 32nd international conference on Very large data bases*, VLDB '06, VLDB Endowment 2006:631–642.

19. Aggarwal CC, Yu PS: **The IGrid index: reversing the dimensionality curse for similarity indexing in high dimensional space**. In *Proceedings of the sixth ACM SIGKDD international conference on Knowledge discovery and data mining*, KDD '00, New York, NY, USA: ACM 2000:119–129.

20. Schölkopf B, Smola AJ, Williamson RC, Bartlett PL: **New support vector algorithms**. *Neural Comput.* 2000, **12**:1207–1245, [http://portal.acm.org/citation.cfm?id=1139689.1139691].

21. Berg OG, von Hippel PH: **Selection of DNA binding sites by regulatory proteins. Statistical-mechanical theory and application to operators and promoters**. *Journal of Molecular Biology* 1987, **193**(4):723–750.

22. Sarkar D, Siddiquee KAZ, Araúzo-Bravo MJ, Oba T, Shimizu K: **Effect of *cra* gene knockout together with *edd* and *iclR* genes knockout on the metabolism in *Escherichia coli***. *Archives of Microbiology* 2008, **190**:559–571, [http://dx.doi.org/10.1007/s00203-008-0406-2]. [10.1007/s00203-008-0406-2].

23. Gusfield D: *Algorithms on Strings, Trees, and Sequences: Computer Science and Computational Biology*. New York, NY, USA: Cambridge University Press 1997.

24. Smith TF, Waterman MS: **Identification of common molecular subsequences**. *Journal of Molecular Biology* 1981, **147**:195 – 197, [http://www.sciencedirect.com/science/article/pii/0022283681900875].

25. Ellson J, Gansner E, Koutsofios L, North S, Woodhull G, Description S, Technologies L: **Graphviz open source graph drawing tools**. In *Lecture Notes in Computer Science*, Springer-Verlag 2001:483–484.

26. Crooks GE, Hon G, Chandonia JM, Brenner SE: **WebLogo: a sequence logo generator**. *Genome Research* 2004, :1188–1190.

27. Ellson J, Gansner E, Koutsofios E, North S, Woodhull G: **Graphviz and dynagraph – static and dynamic graph drawing Tools**. In *Graph Drawing Software*. Edited by Junger M, Mutzel P, Springer-Verlag 2003:127–148.

28. *http://fallabs.com/tokyocabinet/*.

29. *http://jaligner.sourceforge.net*.

30. *http://acs.lbl.gov/software/colt/*.

31. *http://commons.apache.org/*.

32. *http://xstream.codehaus.org/*.

33. *http://dsiutils.dsi.unimi.it*.

34. *http://fastutil.dsi.unimi.it*.

35. *http://java.freehep.org*.

36. *http://code.google.com/p/google-gson/*.

37. *http://code.google.com/p/guava-libraries/*.

38. *http://www.oracle.com/technetwork/database/berkeleydb/overview/index.html*.
